# High-throughput screening of a chalcone library identifies a naked-eye “traffic light” chemosensor for the discrimination of Cu^2+^ and Ni^2+^ in aqueous media

**DOI:** 10.1039/d6ra05695d

**Published:** 2026-08-24

**Authors:** Alexander Ciupa

**Affiliations:** a Materials Innovation Factory, University of Liverpool 51 Oxford Street Liverpool L7 3NY UK ciupa@liverpool.ac.uk

## Abstract

High-throughput screening (HTS) of a 32-membered chalcone library identified C28 as a dual colorimetric and fluorometric chemosensor for Zn^2+^, Cd^2+^, Cu^2+^ and Ni^2+^ in MeCN. Critically, C28 differentiates Cu^2+^ from Ni^2+^ in aqueous media *via* a naked-eye “traffic light” response, with limits of detection of 0.032 µM and 0.198 µM, both below the recommended maximum daily intake levels. Solid-state validation using a dipstick format confirms suitability for portable, instrument-free deployment, with particular relevance to resource-limited or off-grid settings where rapid water quality assessment is required but currently absent. A systematic structure–activity relationship (SAR) study from over 384 datapoints identified the structural requirements for optimum sensor activity unlocking a second-generation library with enhanced performance.

## Abstract 2

High-throughput screening (HTS) of a 32-membered chalcone library identified C28 as a dual colorimetric and fluorometric chemosensor for Zn^2+^, Cd^2+^, Cu^2+^ and Ni^2+^ in MeCN.

## Graphical abstract

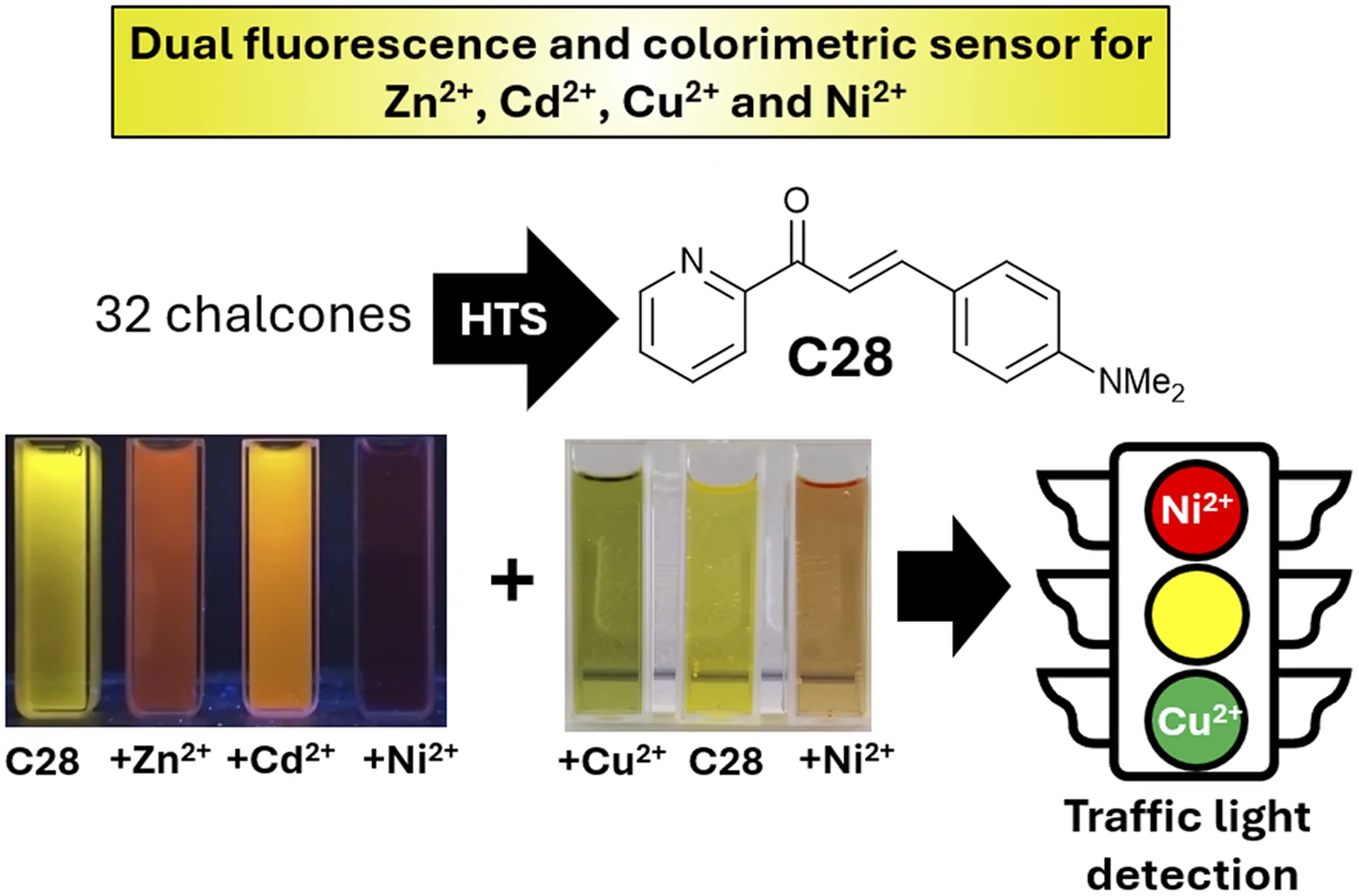

## Introduction

**Fig. 1:**
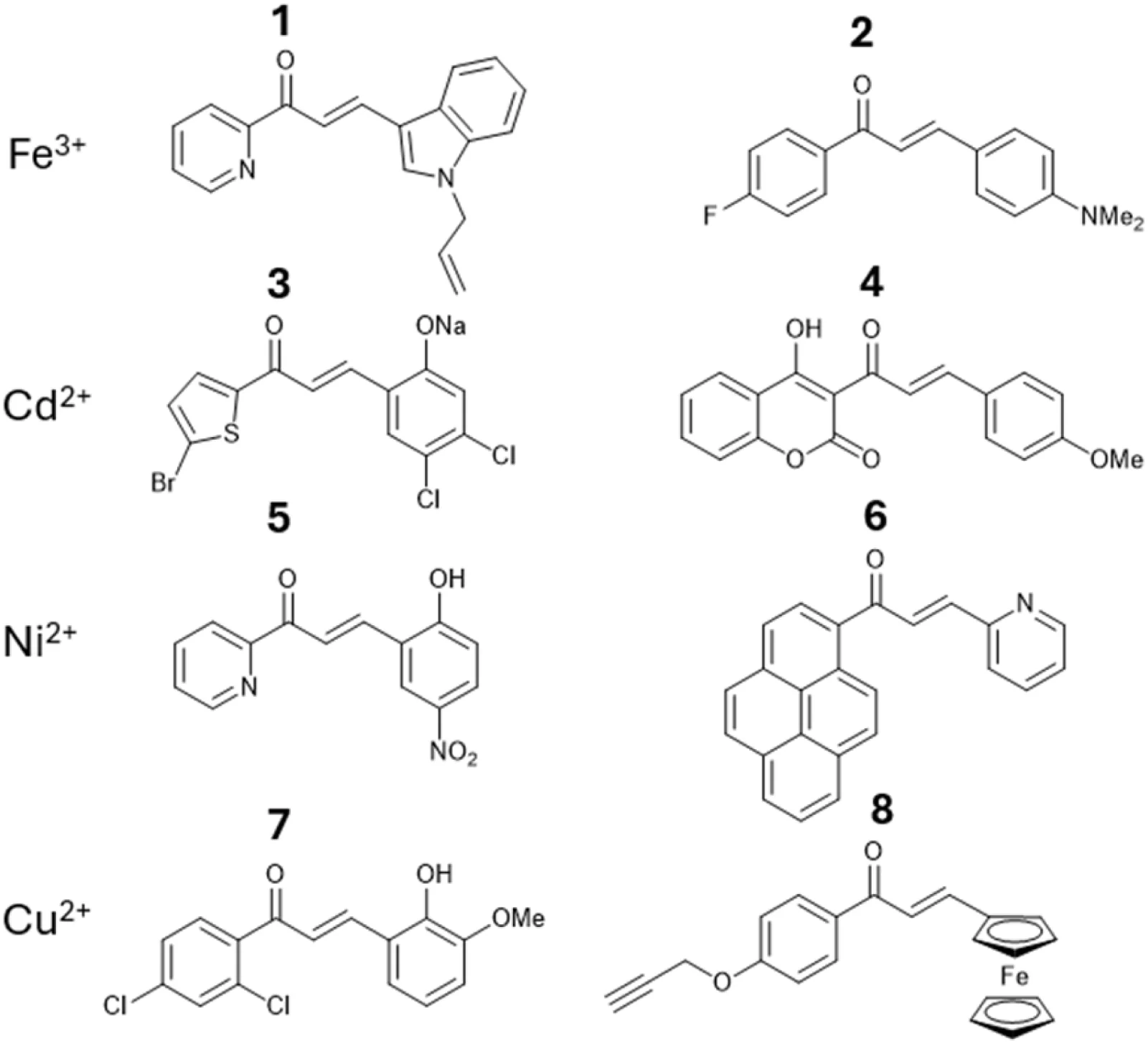
Chalcone fluorescent sensors for toxic metals.

Iron, zinc and copper are the three most abundant transition metals in the human body,^1–3^ involved in a wide range of biological functions including oxygen transport *via* haemoglobin,^4^ enzyme catalysis^5^ and immune function^6^ respectively. The dysregulation and excessive intake of these metals is implicated in a range of diseases: iron overload (haemochromatosis),^7^ and Alzheimer's and Parkinson's diseases.^8^ Regular monitoring of these metals in drinking water and the food chain is essential, with the European Union (EU) having a daily intake guideline of 3.5 µM for iron,^9^ the World Health Organisation (WHO) having a daily intake set at 46 µM for zinc, and there being an EU limit of 31 µM for copper.^10^ Nickel is a trace element that shares intestinal absorption pathways with iron with excessive exposure linked to nausea and vomiting;^11^ therefore an EU drinking water guideline of 0.341 µM (ref. 12) is established. Traditional methods to monitor these metals involve inductively coupled plasma (ICP) spectrometry,^13^ which requires sample collection and transport to centralised laboratories—a time-consuming and expensive process. The development of rapid, point-of-care (POC) diagnostics using low-cost colorimetric and fluorescent sensors is a rapidly emerging area of research.^14^ Multi-analyte sensors are particularly attractive enabling rapid detection and quantification of numerous toxic metals using a single low-cost device. Recent examples include the multi-analyte detection of Cd^2+^, Co^2+^, Ni^2+^ and Fe^3+^ in water,^15^ a dual probe for rapid environmental monitoring of Hg^2+^ and As^3+^ in water,^16^ and fluorescence detection of Cr^3+^, Hg^2+^, Ni^2+^ and Pb^2+^.^17^ Such POC devices are of particular value in rural and developing-world settings, where heavy metal pollution disproportionately impacts low income communities with limited access to traditional screening methods.^18^ The United Nations (UN) estimates that 2 billion people worldwide will lack safely managed drinking water by 2030,^19^ underscoring the urgent need for low-cost, instrument-free POC sensors capable of screening water quality. Chalcones^20^ represent a promising solution with a broad range of sensors reported,^21^ while also serving as precursors to pyrazolines^22^ and pyrazole^23^ colorimetric and fluorescent based sensors. Chalcones 1 and 2 have been reported as fluorescent sensors for Fe^3+^ in aqueous environments (Fig. 1).^24,25^ Chalcones 3 and 4 can detect Cd^2+^,^26,27^ while chalcones 5 and 6 are useful for the detection of nickel (Fig. 1).^28,29^ Chalcones 7 and 8 are effective sensors for Cu^2+^ (Fig. 1).^30,31^

**Fig. 2:**
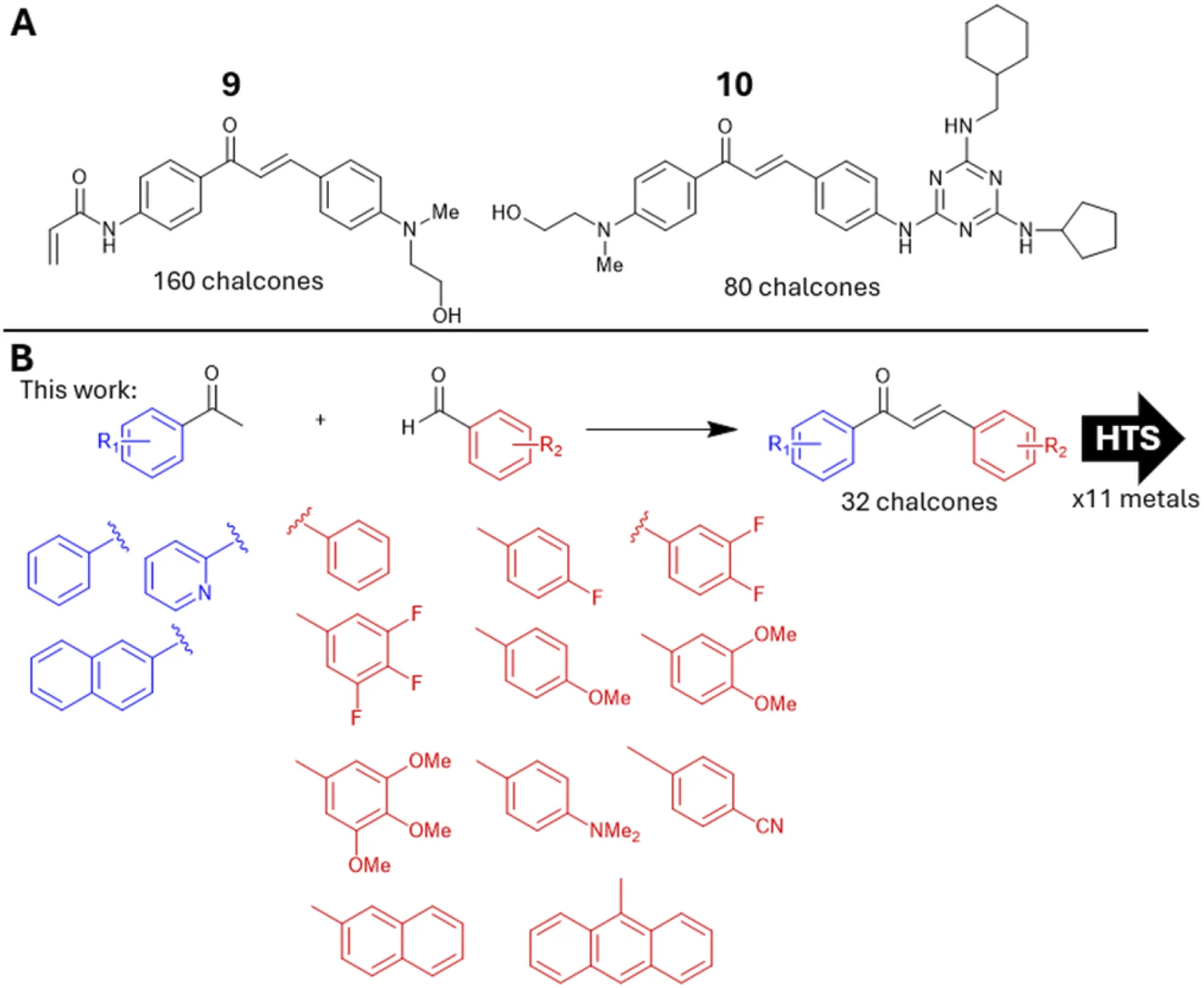
Previous chalcone fluorescent chalcone libraries (panel A) and this work (panel B).

The modularity of the chalcone scaffold, typically accessed *via* a single-step Claisen–Schmidt condensation^32^ from commercially available starting materials, makes it ideal for diversity-oriented library generation. This is demonstrated by chalcone 9, a useful sensor for mouse embryonic stem cell (mESC) staining derived from a 160-membered chalcone library (Fig. 2A).^33^ Similarly, chalcone 10 was identified as a cell membrane permeable biological probe for the cytosol identified by screening a 80-membered chalcone library (Fig. 2A).^34^ These examples highlight the advantages of combining a highly modular scaffold with rapid screening to identify novel sensors. We previously reported a highly efficient synthesis of a 27-membered chalcone library using a design of experiments (DoE) approach, providing gram-scale access to a diverse range of chalcones directly *via* recrystallization.^35^ High-throughput screening (HTS) of the pyrazoline library derived from this library enabled rapid identification of useful fluorescent properties. This study applied a similar HTS approach to a library of 32 chalcones, with the discovery of chalcone C28 with promising dual colorimetric and fluorescent properties capable of detecting and distinguishing Zn^2+^, Cd^2+^, Ni^2+^ in MeCN. Crucially, C28 retains a visually distinct colour change in aqueous environments, enabling naked-eye detection and differentiation of Cu^2+^ and Ni^2+^ using a ‘traffic light’ approach. This rapid, multi-analyte sensor has detection limits below recommended safety guidelines, making it ideal for monitoring water quality in rural areas lacking mains electricity or complex laboratory apparatus.

## Results and discussion

Chalcones C1–C27 were adopted from a previous study^35^ with chalcones C28–C32 specifically synthesized and characterized for this study to explore the influence of highly electron donating and withdrawing substituents (SI 1).

**Fig. 3:**
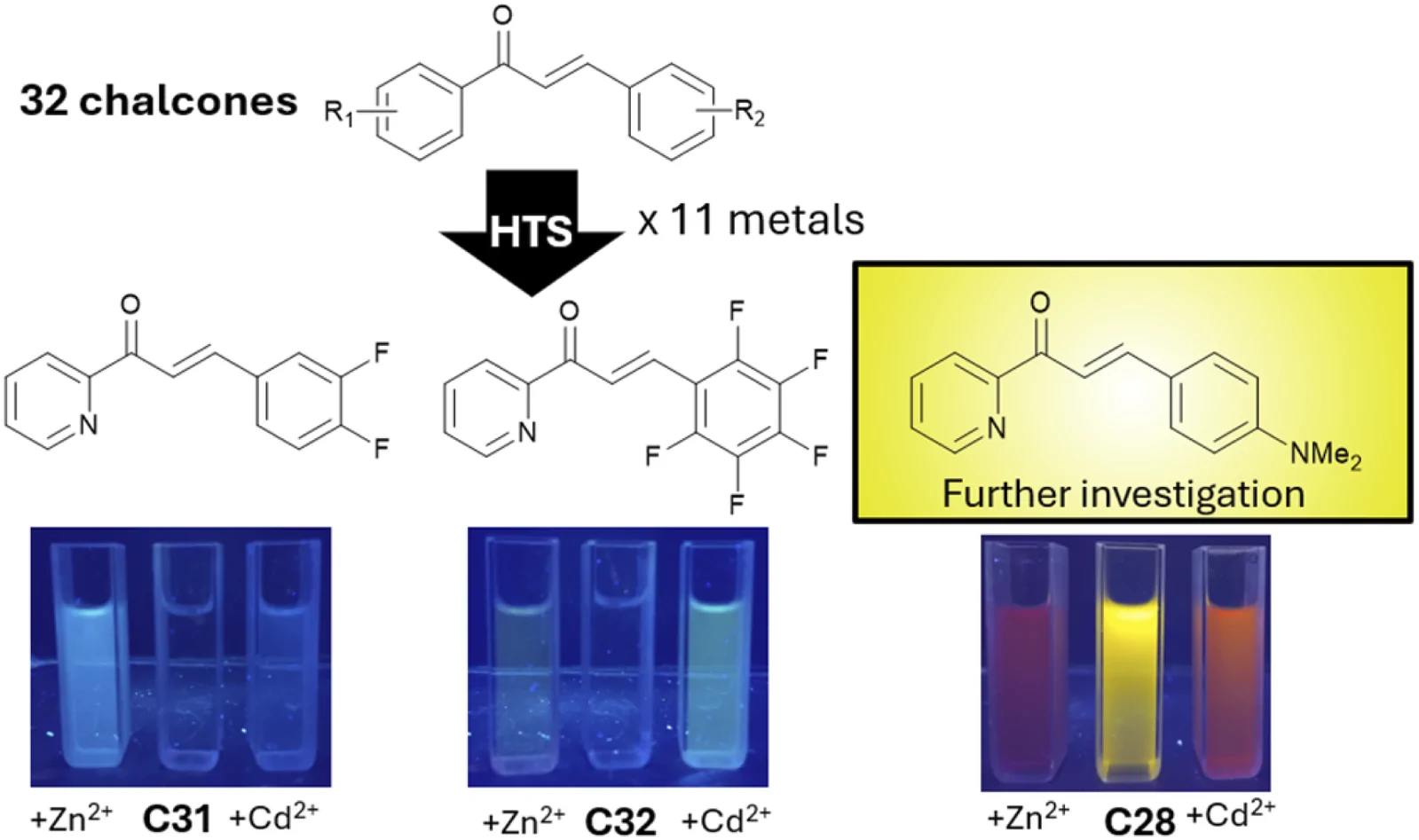
Three hits from the chalcone library with C28 selected for further investigation.

**Fig. 4:**
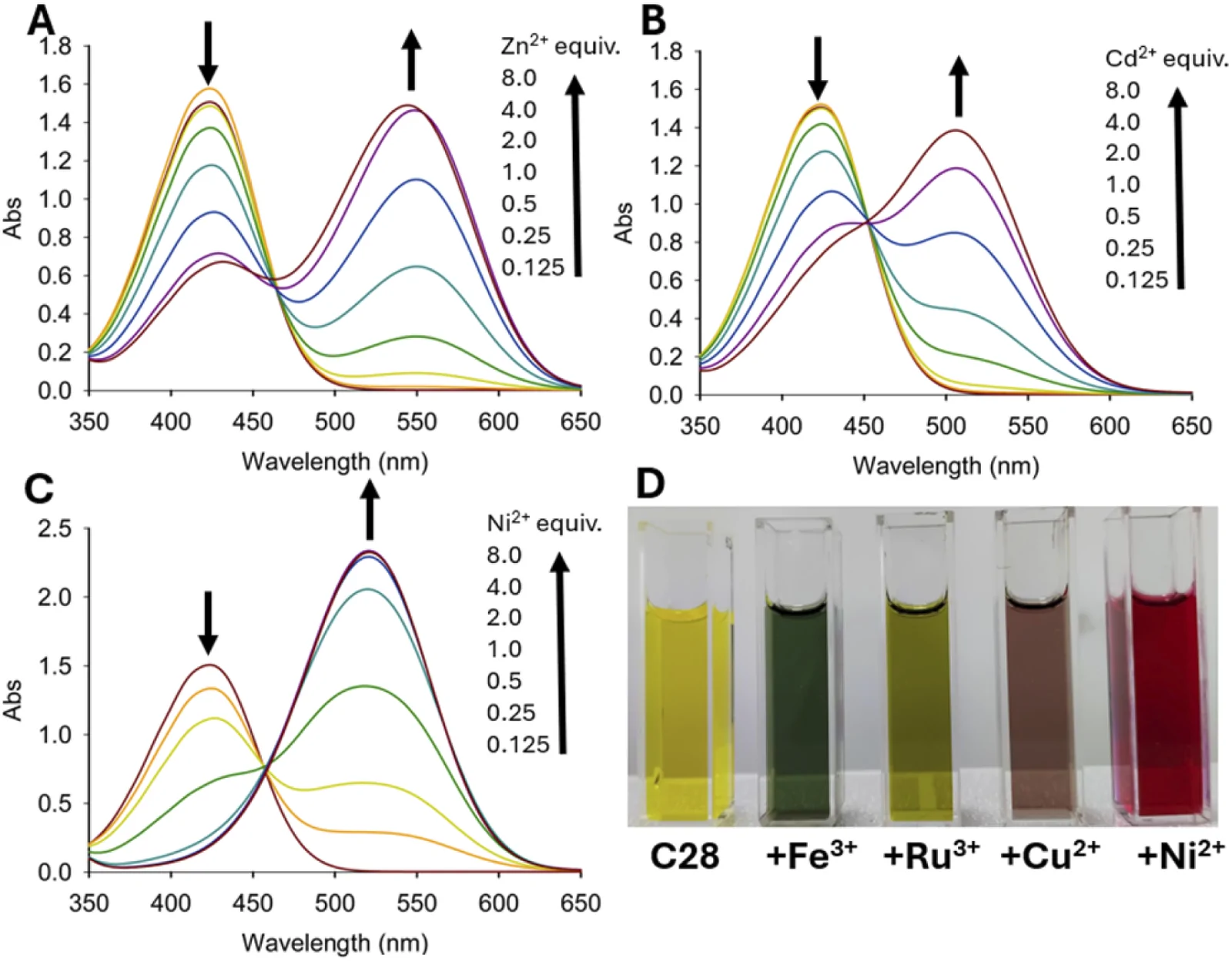
UV/Vis analysis of C28 (50 µM) in MeCN in the presence of increasing Zn^2+^ (panel A), Cd^2+^ (panel B) and Ni^2+^ (panel C). Colorimetric changes on additional of various metals (panel D).

The resulting 32-member library was screened in a 96-well format across eleven metals using a dual UV/Vis and fluorescence plate reader (SI 2). Three chalcones were identified as displaying useful properties, difluorinated C31 displayed a “turn-on” response in the presence of Zn^2+^ only whereas pentafluorinated C32 displayed a Cd^2+^ “turn-on” response (Fig. 3). Chalcone C28 containing the highly electron donating dimethylamino group displayed a distinct Stokes shift of >170 nm with yellow fluorescence emission (*λ*_em_) at 575 nm. The *λ*_em_ is red shifted to 618 nm and 590 nm in the presence of Zn^2+^ and Cd^2+^ respectively (Fig. 3). Such large Stokes shifts are particularly attractive in fluorescent sensor design^36^ and therefore C28 was selected for further investigation. A UV/Vis study confirmed the formation of new absorbance bands in the presence of increasing amounts of Zn^2+^, Cd^2+^ and Ni^2+^ (Fig. 4A–C) and the visible colour change with addition of Fe^3+^, Ru^3+^, Cu^2+^ and Ni^2+^ (Fig. 4D). While C28 has been reported for the treatment of Alzheimer's disease,^37^ its Cu^2+^ complex reported,^38^ and its use as a laser dye examined,^39^ this is the first extensive investigation into the multi-analyte sensing properties for the detection of toxic metals.

**Fig. 5:**
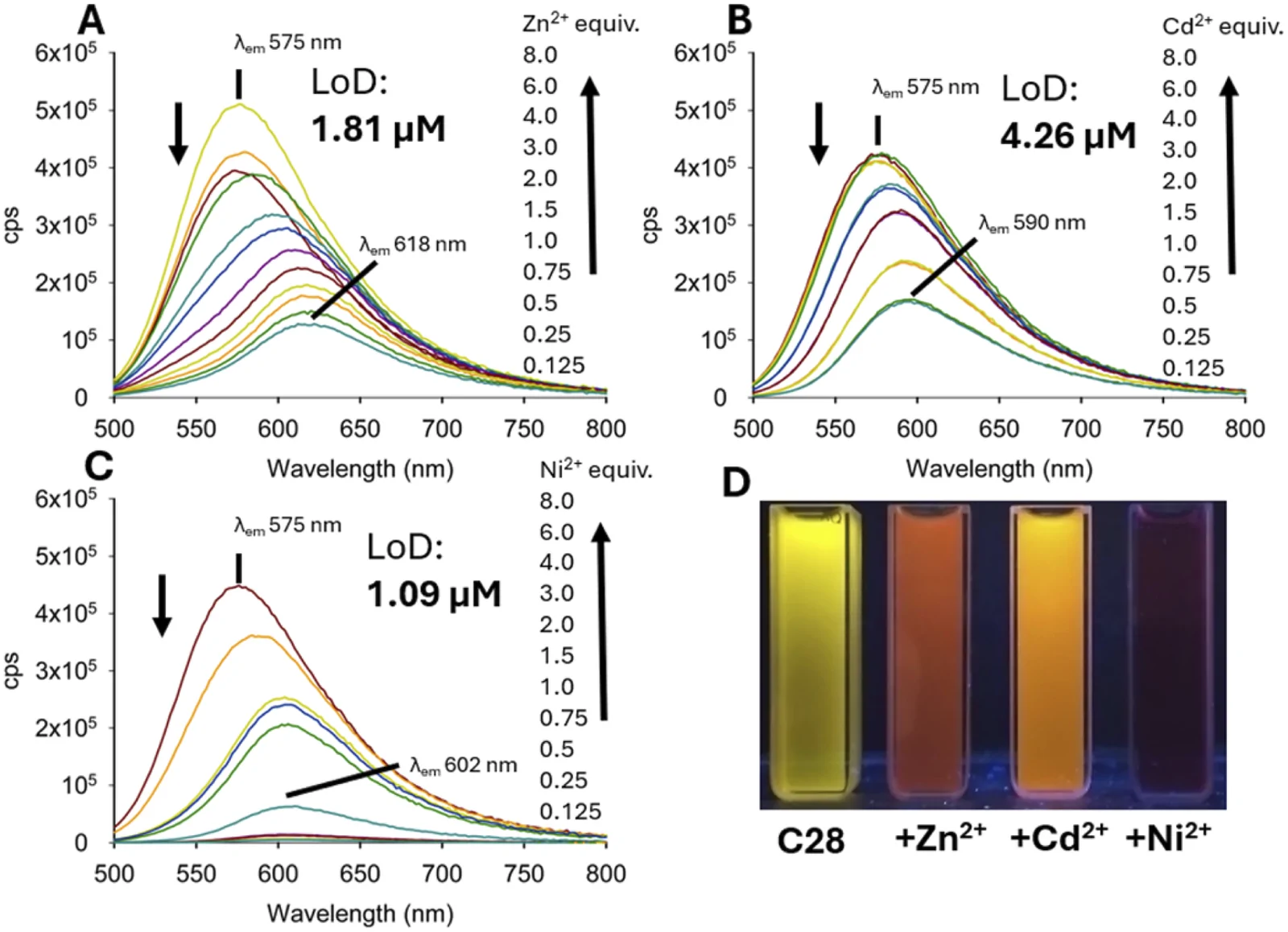
Fluorescence analysis of C28 (50 µM) in MeCN in the presence of increasing amounts of Zn^2+^ (panel A), Cd^2+^ (panel B) and Ni^2+^ (panel C). Visible fluorescence emission of C28 with various metals under *λ*_ex_ 365 nm (100 W) lamp (panel D).

To confirm the potential of C28 as both a colorimetric and fluorescent sensor, a full fluorescence study was performed in the present of increasing amount of Zn^2+^, Cd^2+^ and Ni^2+^ (Fig. 5A–C). The red shift observed in the initial fluorescence study was confirmed with the formation of an emission band at 618 nm with Zn^2+^ and visible red fluorescence under a *λ*_ex_ 365 nm lamp (Fig. 5D). The additional of Cd^2+^ resulted in a new fluorescence band at 590 nm and orange fluorescence suggesting C28 could distinguish between these two group 12 metals. Few sensors have been reported which can differentiate between two different group 12 metals.^40^ The addition of Ni^2+^ resulted in a “turn-off” response, possibly due to the paramagnetic nature of Ni^2+^ quenching the fluorescence response. The limit of detection (LoD) for Zn^2+^, Cd^2+^ and Ni^2+^ in MeCN were calculated as 1.81 µM, 4.26 µM and 1.09 µM respectively (see SI 5).

**Fig. 6:**
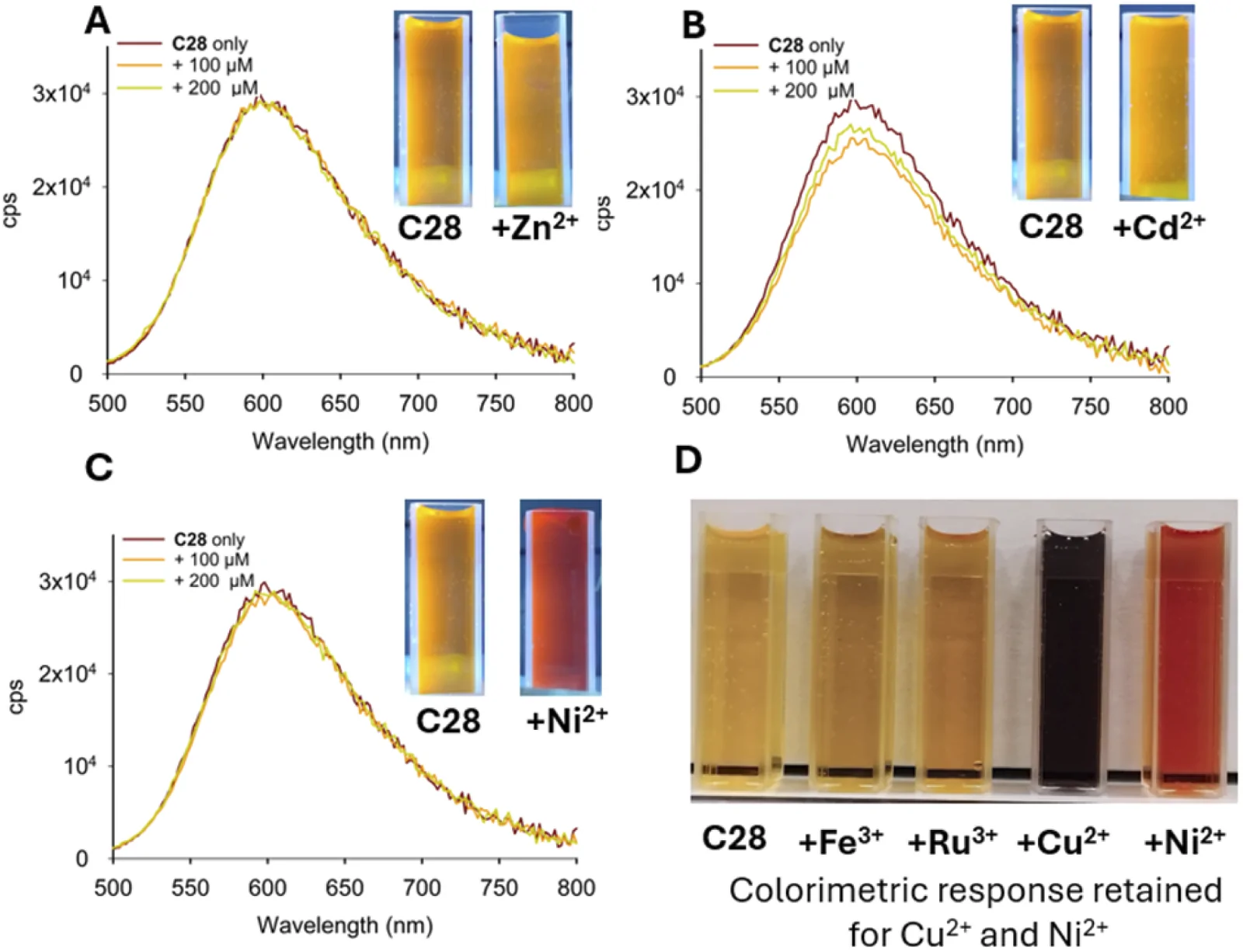
Fluorescence analysis of C28 (50 µM) in 1 : 1 MeCN : H_2_O with 100 µM and 200 µM Zn^2+^ (panel A), Cd^2+^ (panel B) and Ni^2+^ (panel C). Colorimetric changes on additional of various metals retained for Cu^2+^ and Ni^2+^ in 1 : 1 MeCN : H_2_O (panel D).

**Fig. 7:**
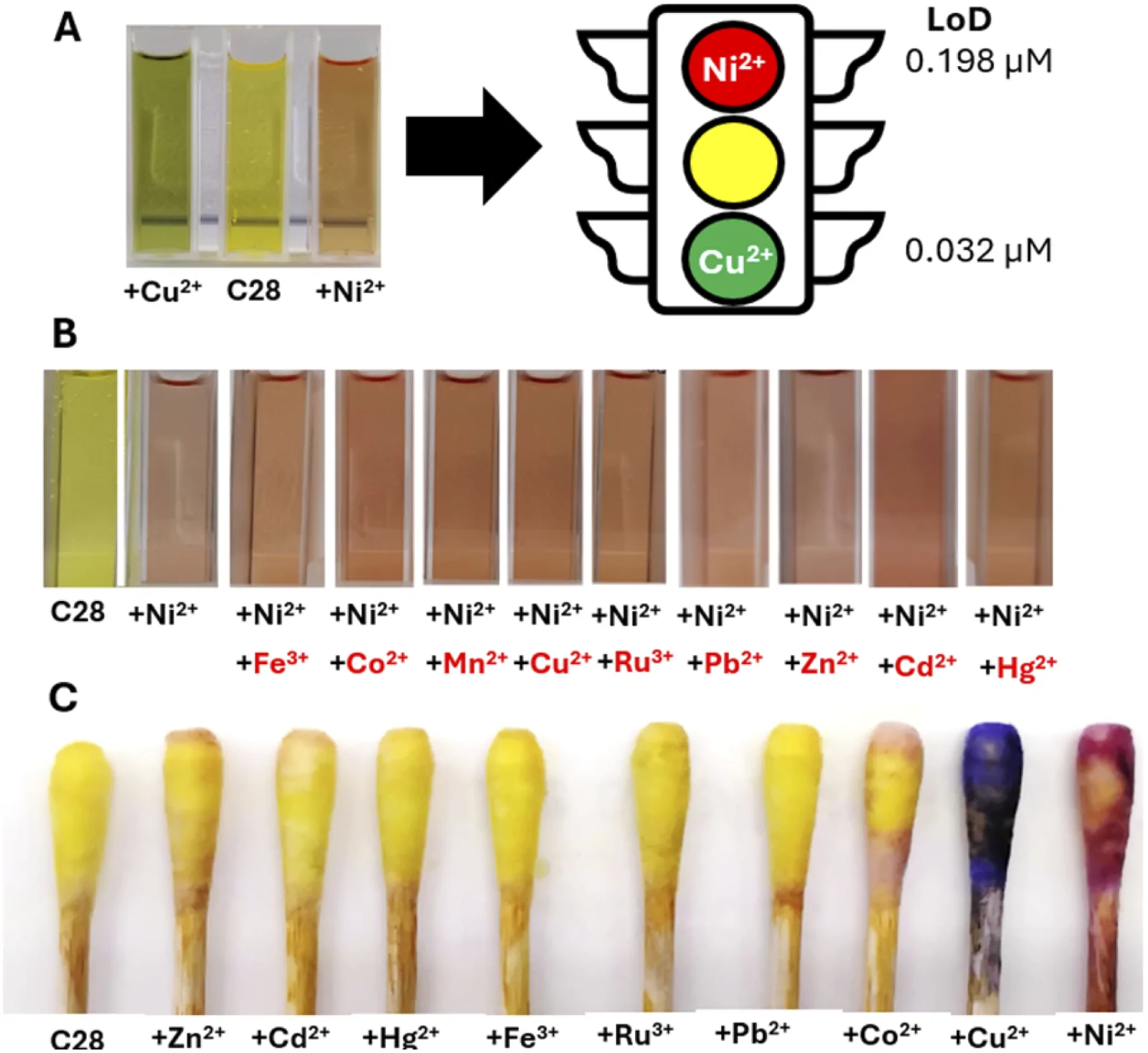
Traffic light style detection of 20 µM Cu^2+^ (green) and 20 µM Ni^2+^ (red) in 1 : 1 MeCN : H_2_O (panel A) and competition assay for Ni^2+^ in the presence of indicated competing ion (panel B). The solid-state detection of 100 µM Cu^2+^ and Ni^2+^ on dipsticks coated with C28 and dried in air for 2 minutes (panel C).

**Fig. 8:**
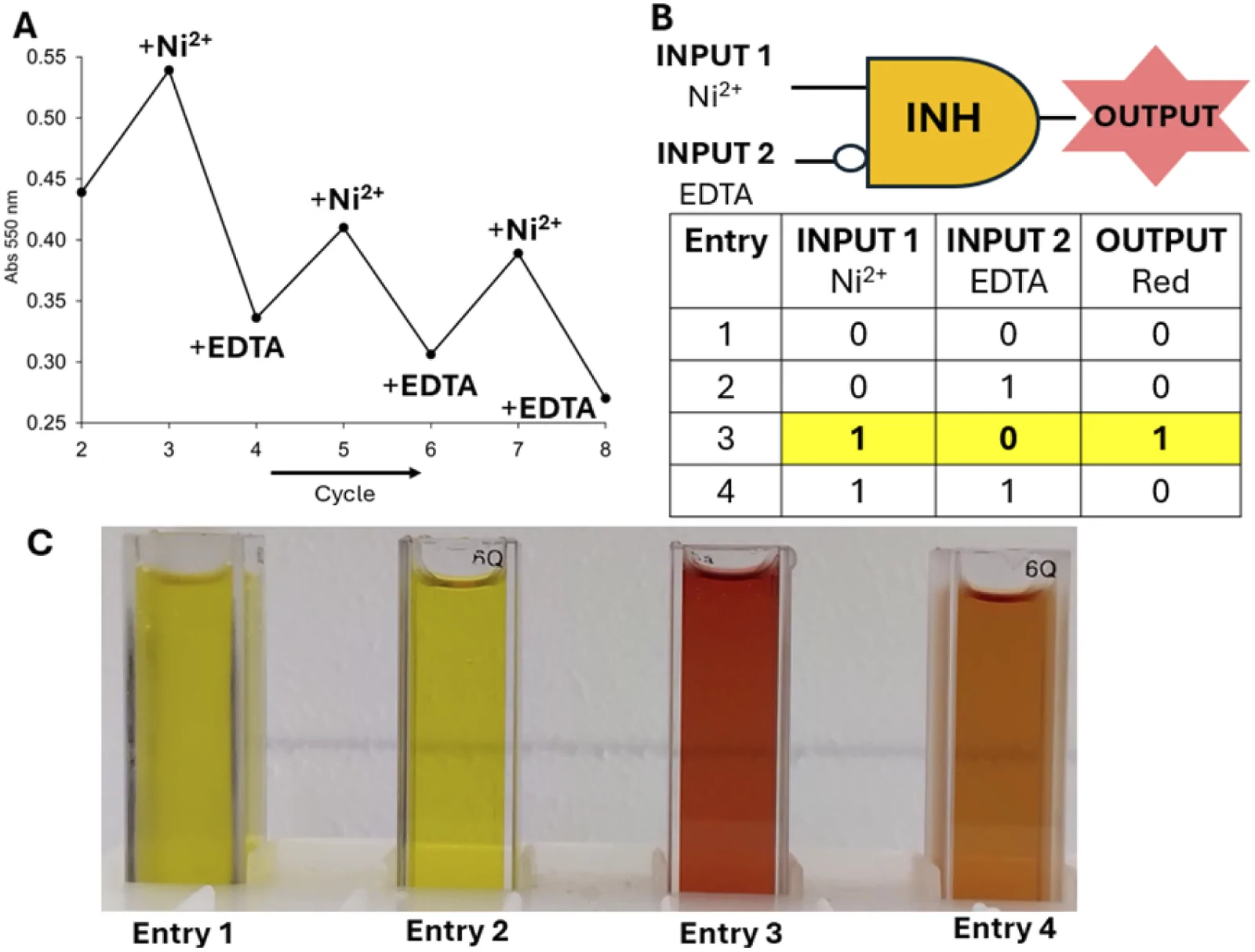
Reversibility study of C28 (50 µM) in 1 : 1 MeCN : H_2_O in the presence of multiple cycles of 1 equiv. Ni^2+^ and EDTA (panel A). C28 displayed INHIBIT logic gate functionality, truth table (panel B) and visible detection (panel C).

Inspired by these initial results, we repeated the analysis in 1 : 1 MeCN : H_2_O solution as fluorescent response in aqueous environments is essential for real-world applications. The addition of water reduced the fluorescence response of C28 itself by over 10-fold and disrupted the previously observed response with Zn^2+^, Cd^2+^ and Ni^2+^ (Fig. 6A–C) Interestingly, the colorimetric response to Cu^2+^ and Ni^2+^ was retained suggesting C28 could be useful for detecting and differentiating these two analytes in aqueous environments by the naked eye (Fig. 6D). A “traffic-light” style response was observed in which C28 itself displayed a yellow colour which shifted to either green with Cu^2+^ or red with Ni^2+^ (Fig. 7A). A competition assay confirmed that C28 was particularly strongly bound to Ni^2+^ and retained its “red light” signal in the presence of a range of different competing ions (Fig. 7B). Application of C28 to cotton wool dipsticks, followed by immersion in various aqueous metal solutions, confirmed that solid-state detection is possible (Fig. 7C). Specifically, a distinct colour change to red occurred with 100 µM Ni^2+^, while a dark blue colour developed with Cu^2+^ after drying in air for two minutes. A similar response was observed with 50 µM Cu^2+^ and Ni^2+^ (SI) suggesting a quick and solvent free method for qualitative analysis of toxic metals. The application of both laboratory based *via* widely available UV/Vis spectrometers and solid-state application *via* dipsticks are available. Alongside the use of the naked eye as the detector smartphones have rapidly emerged as accessible, low-cost alternatives to conventional spectrophotometric instrumentation.^41^ By exploiting a smartphone's built-in camera to capture the colour and intensity of a sample, image analysis (typically *via* the RGB colour model), combined with a calibration curve, allows the concentration of an analyte to be determined. The portability, minimal equipment cost, and availability of smartphones make this approach particularly valuable for researchers in resource-limited settings. The technique has been applied across an increasingly broad range of fields, including heavy metals, herbicides, pesticides, antibiotics, biochemical and clinical indicators, natural compounds, and bacteria/viruses.^42^ Recent examples include a smartphone app for the simultaneous colorimetric detection of arsenic and mercury,^43^ gold nanoparticle-based sensing of Fe^3+^,^44^ and tetracycline detection *via* smartphone digital imaging.^45^C28 would be well suited to such an approach. A reversibility study confirmed the binding of C28 to Ni^2+^ was reversible on the addition of 1.0 equiv. EDTA and the colorimetric response could be regenerated multiple times. This suggested C28 is a reusable sensor and may have application as a molecular logic gate.^46^ The INHIBIT truth table was applicable to this sensor (Fig. 8B and C) and is analogous to chalcone 7 (Fig. 1) reported by Singh and Chandra^30^ which displayed a INHIBIT gate functionality with Cu^2+^ and EDTA as inputs 1 and 2 respectively.

**Fig. 9:**
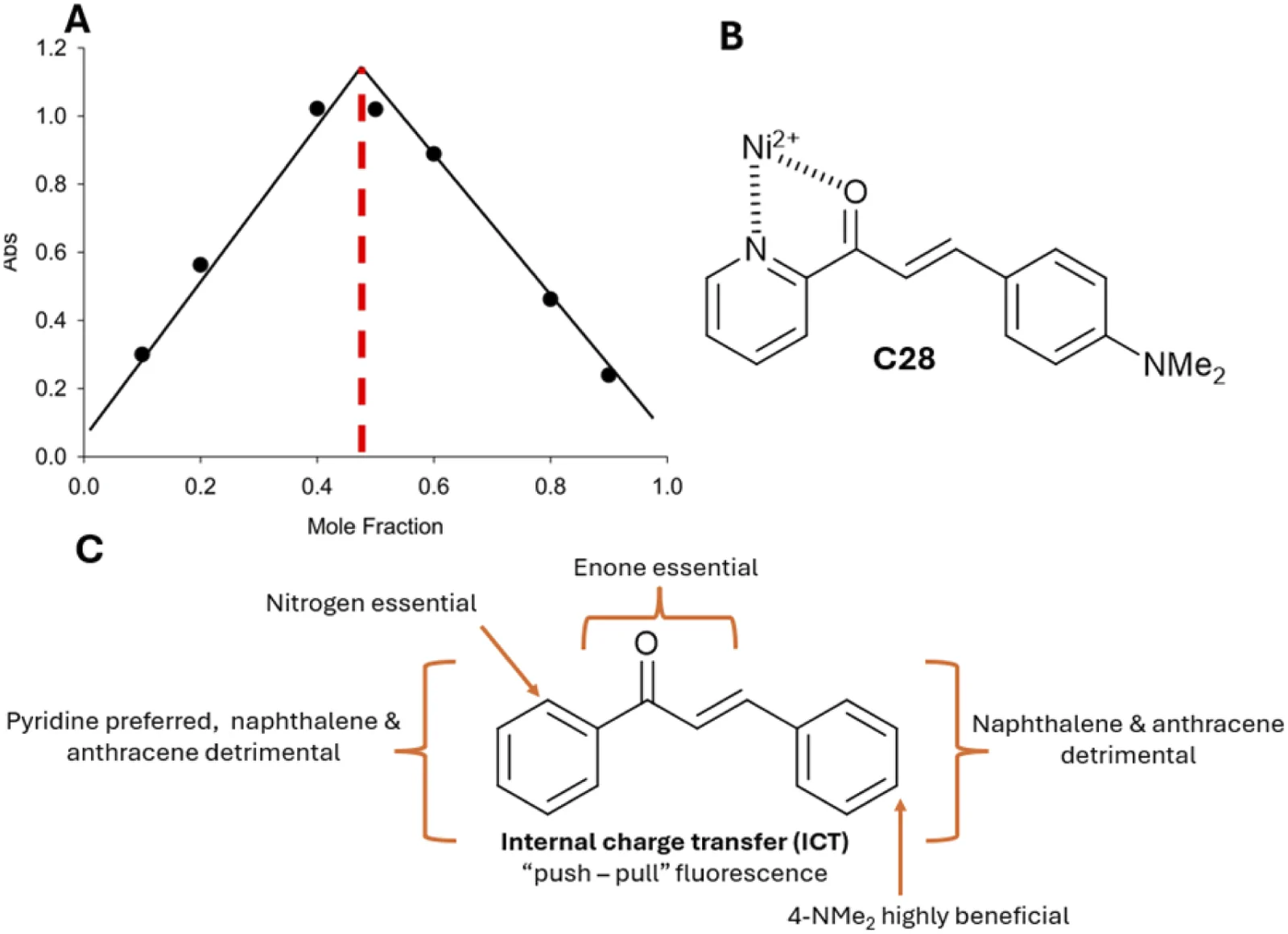
Job plot for C28 with Ni^2+^ with 1 : 1 binding ratio (panel A) with proposed binding site (panel B). Structural–activity relationship (SAR) derived from HTS of the 32-membered chalcone library (panel C).

Singh *et al.* also reported a chalcone based INHIBIT molecular gate with Co^2+^ and EDTA inputs.^47^ While chalcones which chelate Ni^2+^ have been reported previously,^28,29,48–50^ this is the first example of a chalcone based INHIBIT molecular logic gate using Ni^2+^ with naked eye detection. A recent review by Ai e*t al.*^51^ highlights the significant advantages molecular logic gates provide in heavy metal analysis in water and their potential to replace traditional based methods. Chalcone C28 is a promising example worthy of further development. Job plot analysis determined that the binding ratio of C28 to Ni^2+^ and Cu^2+^ was 1 : 1 (Fig. 9A for Ni^2+^ see SI for Cu^2+^). The proposed binding mode involved coordination *via* both the pyridine nitrogen and enone oxygen (Fig. 9B). This is evidenced by the fact chalcone C29, lacking a pyridine did not display a change in fluorescence on addition of metal ion (SI 1). Furthermore, a detailed study by Fayed and Gaber^52^ confirmed a similar 1 : 1 binding mode for C28 with Cu^2+^, Zn^2+^ and Cd^2+^. A multitude of analysis including elemental, thermal, and molecular orbital calculations confirm C28 is a bidentate ligand coordinating to the metal specifically *via* the oxygen and nitrogen atoms on the enone and pyridyl unit.^52^

**Fig. 10:**
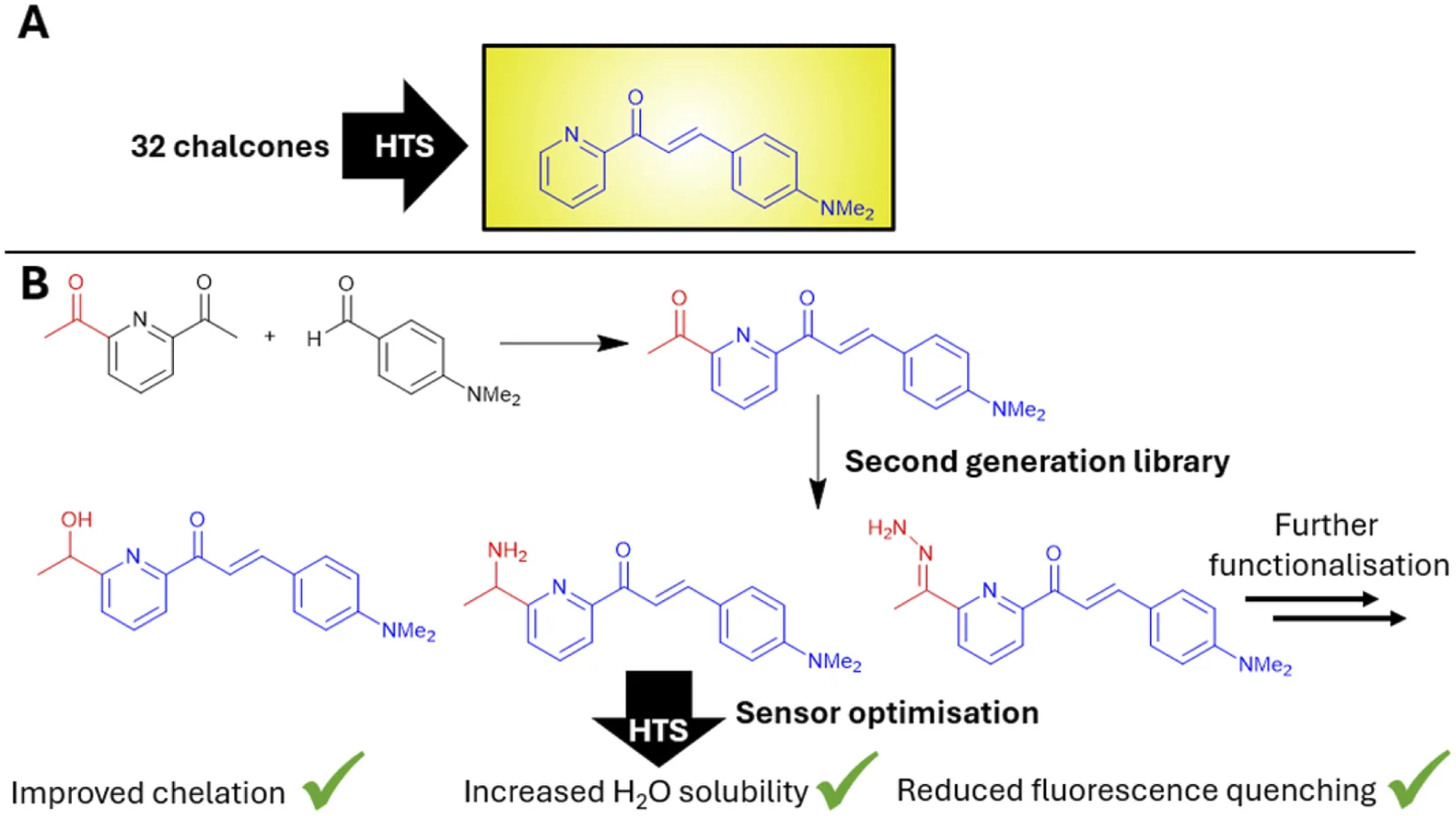
Sensor identification and validation (panel A) and proposed second generation library derived from commercially available 2,6-diacetylpyridine for improved properties (panel B).

The Job plot and HTS study from this 32-member HTS supports this conclusion. Further analysis of the HTS results allowed a preliminary qualitative analysis structural–activity relationship (SAR) to be determined to predict the optimal functional groups for chalcone sensor sensors (Fig. 9C). Chalcones typically display fluorescence *via* internal charge transfer (ICT)^53^*via* a push–pull mechanism^54^ therefore the presence of both electron donating and withdrawing groups is essential. The enone provides the vital bridge for these dynamics and is essential for fluorescence response.^55^ Using this SAR, the findings from this study and the previous works,^38,39,47^ a second library is proposed incorporating the core of C28 (Fig. 10A) with an additional acetyl functional group (Fig. 10B). This acetyl group serves as a versatile chemical handle, readily convertible into a broad range of functional groups to further optimise sensing performance. As additional sensors are developed, the SAR can be further developed to increase the confidence in future sensor design. Furthermore, the acetyl group provides a direct route for covalent immobilisation onto solid supports, enabling the development of lateral flow devices for rapid, low-cost and instrument-free water quality analysis. This work is ongoing and will be reported in due course.

## Conclusion

A 32-membered chalcone library was screened for sensing properties using a high-throughput screening (HTS) approach, leading to the identification of three promising leads: C28, C31, and C32 (Fig. 3). Due to its exceptionally large Stokes shift, C28 was selected for further evaluation and confirmed to display dual colorimetric and fluorogenic sensing capabilities toward a range of metal ions (Zn^2+^, Cd^2+^, Ni^2+^ and Cu^2+^) in MeCN. Although the addition of water quenched the fluorescence response to Zn^2+^, Cd^2+^, Ni^2+^, colorimetric detection of Cu^2+^ and Ni^2+^ was successfully retained in a 1 : 1 MeCN : H_2_O mixture. Binding studies confirmed a reversible 1 : 1 stoichiometry for both Cu^2+^ and Ni^2+^ facilitating a distinct “traffic-light” naked-eye readout. Furthermore, fabricating C28-based cotton wool dipsticks provided a rapid, user-friendly method to detect these ions in under two minutes. This low-cost approach is highly attractive for decentralized water quality analysis without dependence on complex laboratory infrastructure. Evaluation of the 32-member library established a clear structure–activity relationship (SAR), identifying optimum structural features to guide future design. Backed by these insights, a second-generation library is proposed incorporating additional chelation motifs onto the highly modular chalcone scaffold, with the aim of improving aqueous solubility, reducing fluorescence quenching and enhancing metal ion affinity. This future work will bring this platform closer to delivering the simple, cost-effective point-of-care devices urgently needed for global water quality monitoring.

## Author contributions

Alexander Ciupa designed, synthesized, characterised, performed all spectroscopy studies and authored the manuscript.

## Conflicts of interest

There are no conflicts to declare.

## Supplementary Material

RA-016-D6RA05695D-s001

## Data Availability

The data supporting this article have been included as part of the supplementary information (SI). Supplementary information is available. See DOI: https://doi.org/10.1039/d6ra05695d.
