# Supplementary material for: High-throughput screening of a chalcone library identifies a naked-eye “traffic light” chemosensor for the discrimination of Cu2+ and Ni2+ in aqueous media
Source: RSC Adv. 2026 Aug 24;16(42):50144–9. doi: 10.1039/d6ra05695d (PMC13502242; doi:10.1039/d6ra05695d)
Supplement: Supplementary file 1 [file RA-016-D6RA05695D-s001.pdf]

Supplementary Information (SI) for RSC Advances.
This journal is © The Royal Society of Chemistry 2026

Electronic Supplementary Information (ESI)

High-throughput screening of a chalcone library identifies a naked-eye "traffic light" chemosensor for the discrimination of Cu^{2+} and Ni^{2+} in aqueous media

Alexander Ciupa*

General Experimental

Chemicals, solvents and reagents were purchased from commercial sources and used without further purification. PE refers to petroleum ether, bp 40-60 °C. Spectroscopy was performed with CHROMASOLV® gradient grade acetonitrile for HPLC, ≥99.9%, from Sigma-Aldrich.

The metal complexes used in this study were: LiCl, NaCl, KCl, $CaCl_2$, $MgCl_2$, $CuCl_2$, $NiCl_2$, $ZnCl_2$, $CdCl_2$, $RuCl_3$, $CoCl_2$, $MnCl_2$, $PbCl_2$, $ZnCl_2$ and HgI_2.

NMR spectra were obtained on a Bruker Avance III (400 MHz) spectrometer and processed via TopSpin® software. The chemical shifts are recorded in parts per million (ppm) with reference to tetramethylsilane. The coupling constants J are quoted to the nearest 0.5 Hz and are not corrected.

High resolution Mass spectroscopy was performed on Bruker Quadrupole Time-of-Flight (qToF) mass spectrometer.

UV/Vis spectroscopy was performed on an Agilent Cary5000 in quartz cuvettes with a 1 cm pathlength using HPLC grade MeCN, 250-500 nm range with 0.2 sec dwell time. Detector switchover occurred at 350 nm.

FTIR spectroscopy was performed on a Bruker VERTEX 70 spectrometer.

Fluorescence spectroscopy was performed on an Edinburgh Instruments FLS1000 with a xenon excitation source, 2 nm bandwidths for both excitation and emission monochromator, scan speed of 1 nm and dwell time of 0.2 sec. Fluorescence quartz cuvettes with a 1 cm pathlength were used throughout with HPLC grade MeCN.

A 100 Watt 365 nm Analytikjena High intensity UV lamp was used to image the sensors in cuvettes with 5.0 equivalent indicated metal, sensor concentration was 20 μM, solvent was MeCN.

All figures were plotted using SigmaPlot® 14.5 software.

Chalcone Library (ESI S1)

Chalcone **C1-C27**

The following chalcones were used from a previous study (*Org. Biomol. Chem.*, 2026, **24**, 873) with purity confirmed by LC-MS prior to analysis (See ESI 3 for LC-MS data).

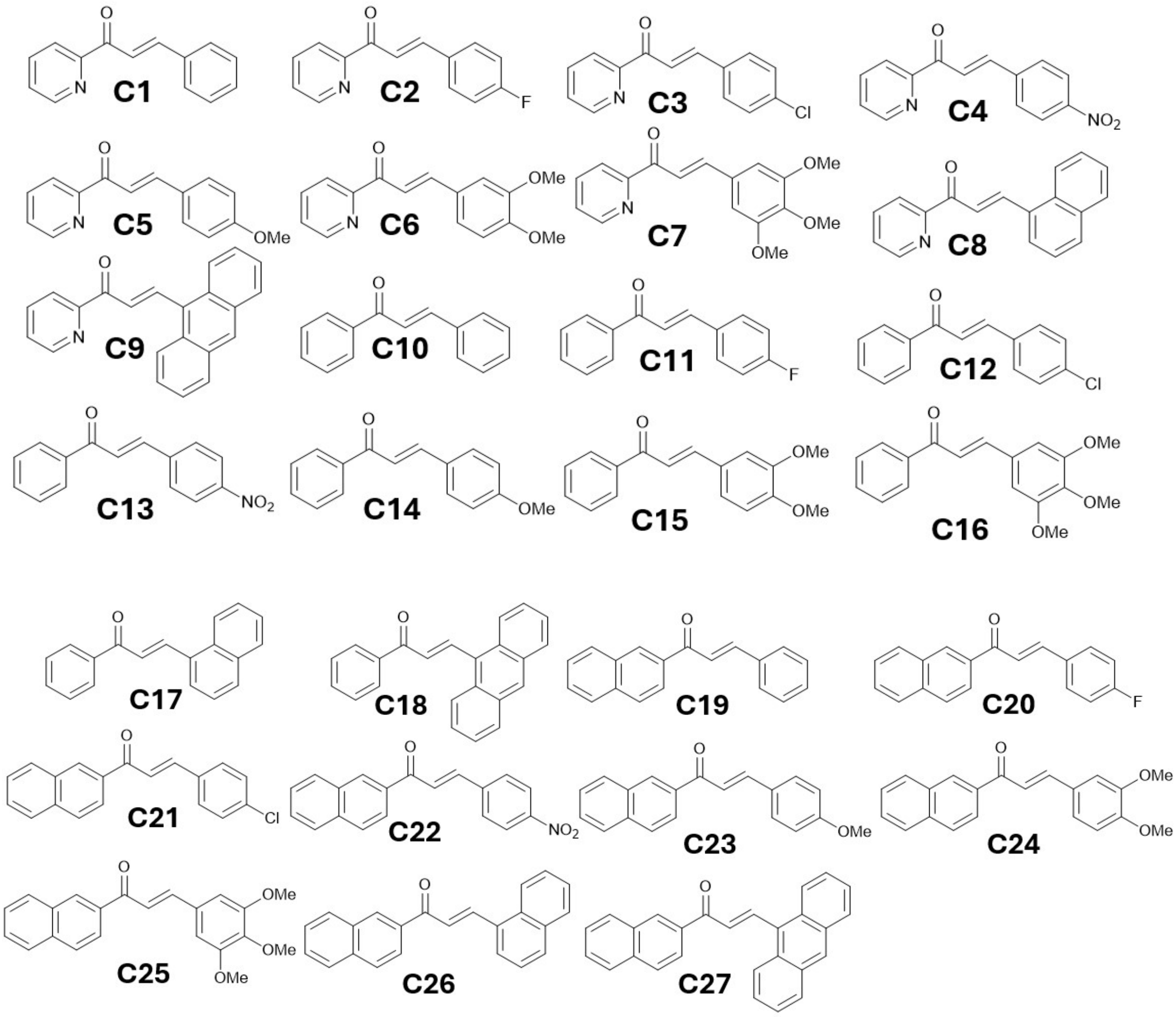

Chalcone **C28-C32**

The following chalcones were synthesised specifically for this study using the DoE method applied to C1-C27 (*Org. Biomol. Chem.*, 2026, **24**, 873)

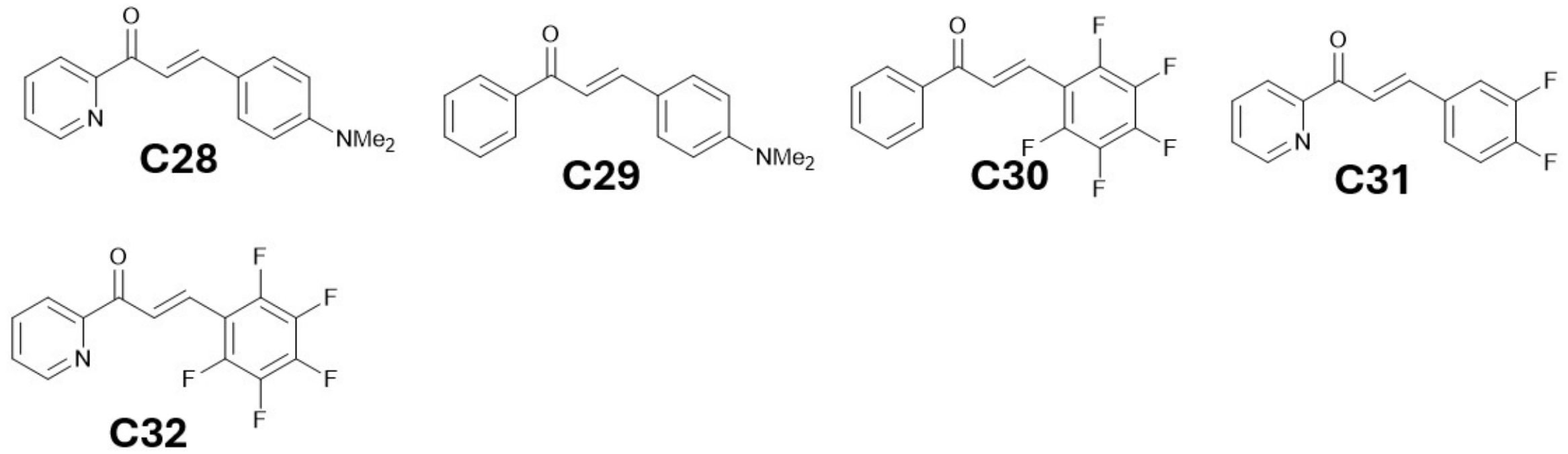

C28

Yield (30%)

^{1}H NMR δ_H (400 MHz; $CDCl_3$) 3.07 (6 H, s, CH_3), 6.72 (2 H, d, *J* = 8.8 Hz, CH), 7.48 (1 H, m, CH), 7.67 (1 H, d, *J* = 8.8 Hz, CH), 7.89 (1 H, t, *J* = 7.8 Hz, CH), 7.96 (1H, d, *J* = 15.6 Hz, CH=CH), 8.10 (1 H, d, *J* = 15.6 Hz, CH=CH), 8.21 (1 H, d, *J* = 7.6 Hz, CH) and 8.75 (1 H, d, *J* = 4.0 Hz, CH);

^{13}C NMR δ_c (100 MHz; $CDCl_3$) 40.1, 111.8, 115.5, 122.8, 123.1, 126.4, 130.9, 136.9, 146.1, 148.7, 152.1, 155.1 and 189.2;

HRMS m/z Found 253.3028 ($M+H^+$). $C_{16}H_{17}N_2O$ requires 253.3250.

Above in agreement with previous data from *Chem. Biodiversity*, 2021, **18**, 2100341.

C29

Yield (35%)

^{1}H NMR δ_H (400 MHz; $CDCl_3$) 3.07 (6 H, s, CH_3), 6.73, (2 H, d, *J* = 9.2 Hz, CH), 7.36 (1 H, d, *J* = 15.6 Hz, CH=CH), 7.49-7.59 (5 H, m, CH), 7.82 (1 H, d, *J* = 15.6 Hz, CH=CH), 8.03 (2 H, d, *J* = 9.2 Hz, CH);

^{13}C NMR δ_c (100 MHz; $CDCl_3$) 40.2, 111.9, 117.0, 122.7, 128.3, 128.5, 130.4, 132.1, 139.1, 145.9, 152.1 and 190.7;

HRMS m/z Found 252.1221 ($M+H^+$). $C_{17}H_{18}N_2O$ requires 252.1388.

Above in agreement with previous data from *New J. Chem*., 2022, **46**, 15392 and *Dyes Pigm*., 2025, **239**,112773.

C30

Yield (17%)

^{1}H NMR δ_H (400 MHz; $CDCl_3$) 7.53-7.56 (2 H, m, CH), 7.62-7.63 (1 H, m, CH), 7.82 (2 H, m, CH), 8.04 (2 H, m, CH);

^{13}C NMR δ_c (100 MHz; $CDCl_3$) 127.5, 128.0, 128.1, 128.6, 128.8, 128.9, 133.3, 133.4, 133.5, 137.6 and 189.7;

HRMS m/z Found 299.0532 ($M+H^+$). $C_{15}H_{18}F_5O$ requires 299.0496.

C31

Yield (71%)

^{1}H NMR δ_H (400 MHz; $CDCl_3$) 7.20-7.25 (1 H, m, CH), 7.51-7.52 (1 H, m, CH), 7.52-7.53 (1 H, m, CH), 7.54-7.58 (1 H, m, CH), 7.83 (1 H, d, *J* = 16.0 Hz, CH=CH), 7.88-7.91 (1 H, m, CH), 8.19-8.22 (1 H, m, CH), 8.25 (1 H, d, *J* = 16.0 Hz, CH=CH), 8.75-8.77 (1 H, m, CH);

^{13}C NMR δ_c (100 MHz; $CDCl_3$) 116.7, 116.9, 117.7, 117.9, 121.8, 121.9, 123.0, 132.5, 137.1, 142.2, 148.9, 153.9 and 189.1;

HRMS m/z Found 246.0832 (M+H$^+$). $C_{14}H_{10}FNO$ requires 246.0730.

C32

Yield (47%)

^{1}H NMR δ_H (400 MHz; $CDCl_3$) 7.53-7.56 (1 H, m, CH), 7.85-7.94 (2 H, m, CH=CH and CH), 8.20-8.22 (1 H, m, CH), 8.57 (1 H, d, *J* = 16.4 Hz, CH), 8.77-8.79 (1 H, m, CH);

^{13}C NMR δ_c (100 MHz; $CDCl_3$) 123.0, 123.1, 127.2, 127.3, 127.4, 127.5, 128.6, 137.0, 137.1, 149.1, 153.5 and 189.2;

HRMS m/z Found 300.0654 (M+H$^+$). $C_{14}H_7F_5NO$ requires 300.0448.

NMR Spectra (ESI S2)

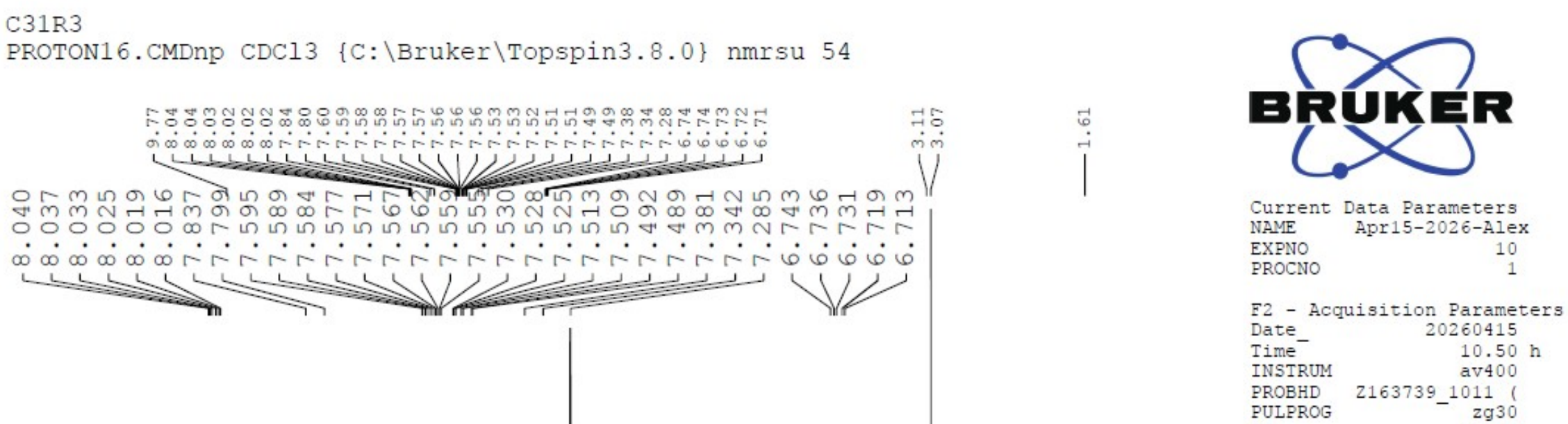

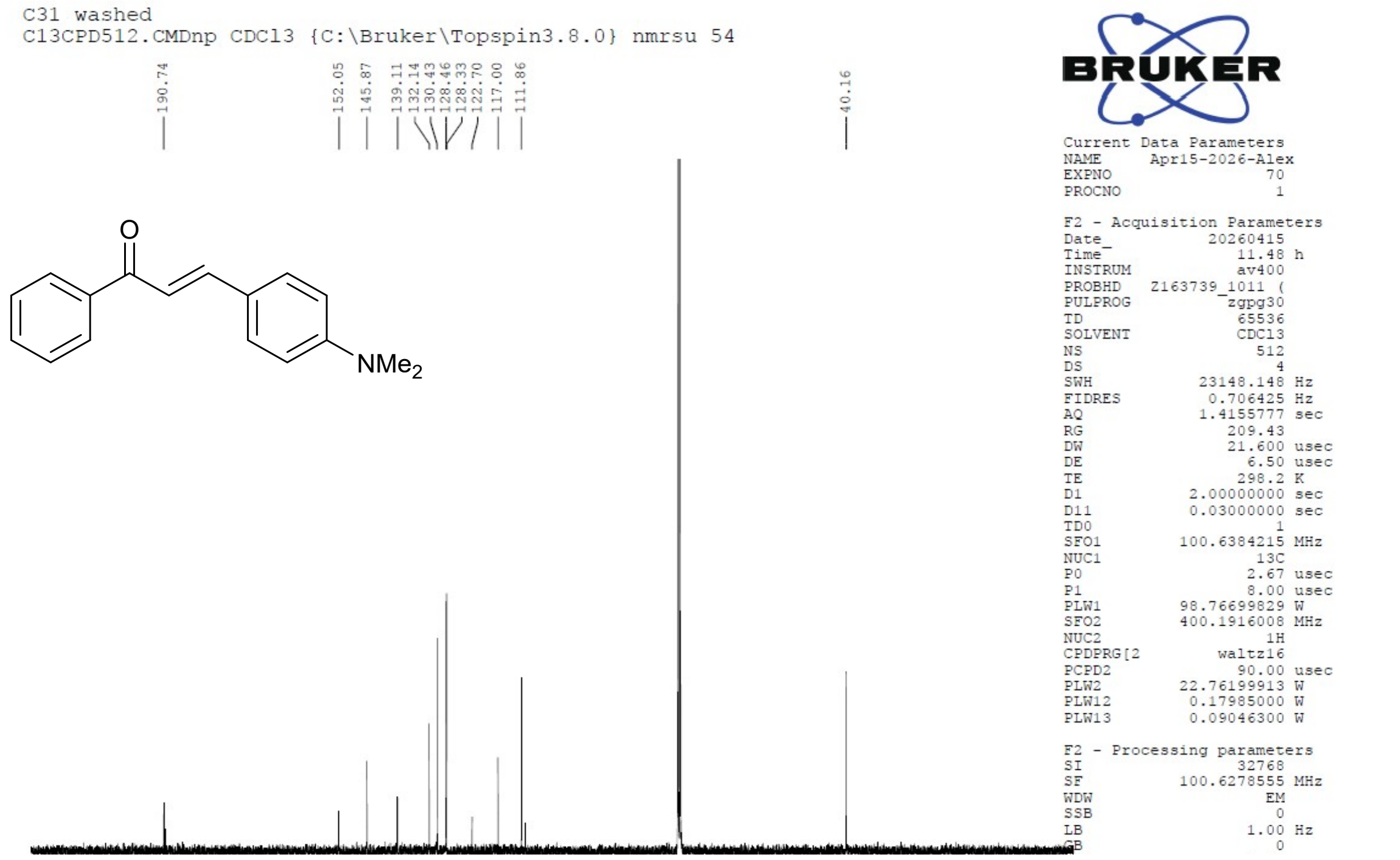
C31 washed
C13CPD512.CMDnp CDCl3 {C:\Bruker\Topspin3.8.0} nmrsu 54
190.74
152.05
145.87
139.11
132.14
130.43
128.46
128.33
122.70
117.00
111.86
40.16
O
NMe2
BRUKER
Current Data Parameters
NAME Apr15-2026-Alex
EXPNO 70
PROCNO 1
F2 - Acquisition Parameters
Date_ 20260415
Time 11.48 h
INSTRUM av400
PROBHD Z163739_1011 (
PULPROG zgpg30
TD 65536
SOLVENT CDCl3
NS 512
DS 4
SWH 23148.148 Hz
FIDRES 0.706425 Hz
AQ 1.4155777 sec
RG 209.43
DW 21.600 usec
DE 6.50 usec
TE 298.2 K
D1 2.00000000 sec
D11 0.03000000 sec
TD0 1
SFO1 100.6384215 MHz
NUC1 13C
P0 2.67 usec
P1 8.00 usec
PLW1 98.76699829 W
SFO2 400.1916008 MHz
NUC2 1H
CPDPRG[2 waltz16
PCPD2 90.00 usec
PLW2 22.76199913 W
PLW12 0.17985000 W
PLW13 0.09046300 W
F2 - Processing parameters
SI 32768
SF 100.6278555 MHz
WDW EM
SSB 0
LB 1.00 Hz
GB 0

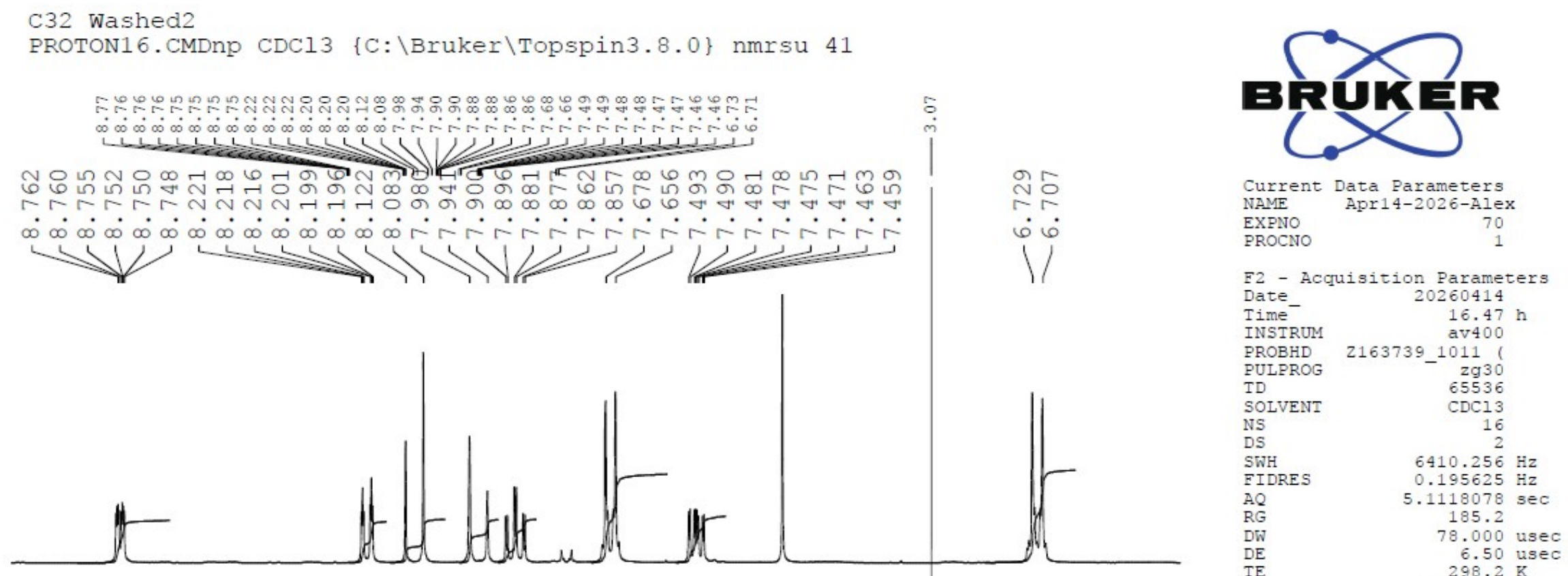
C32 Washed2
PROTON16.CMDnp CDCl3 {C:\Bruker\Topspin3.8.0} nmrsu 41
8.762
8.760
8.755
8.752
8.750
8.748
8.221
8.218
8.216
8.201
8.199
8.196
8.122
8.083
7.980
7.941
7.900
7.896
7.881
7.877
7.862
7.857
7.678
7.656
7.493
7.490
7.481
7.478
7.475
7.471
7.463
7.459
3.07
6.729
6.707
BRUKER
Current Data Parameters
NAME Apr14-2026-Alex
EXPNO 70
PROCNO 1
F2 - Acquisition Parameters
Date_ 20260414
Time 16.47 h
INSTRUM av400
PROBHD Z163739_1011 (
PULPROG zg30
TD 65536
SOLVENT CDCl3
NS 16
DS 2
SWH 6410.256 Hz
FIDRES 0.195625 Hz
AQ 5.1118078 sec
RG 185.2
DW 78.000 usec
DE 6.50 usec
TE 298.2 K

C32 Washed2
C13CPD512.CMDnp CDCl3 {C:\Bruker\Topspin3.8.0} nmrsu 41

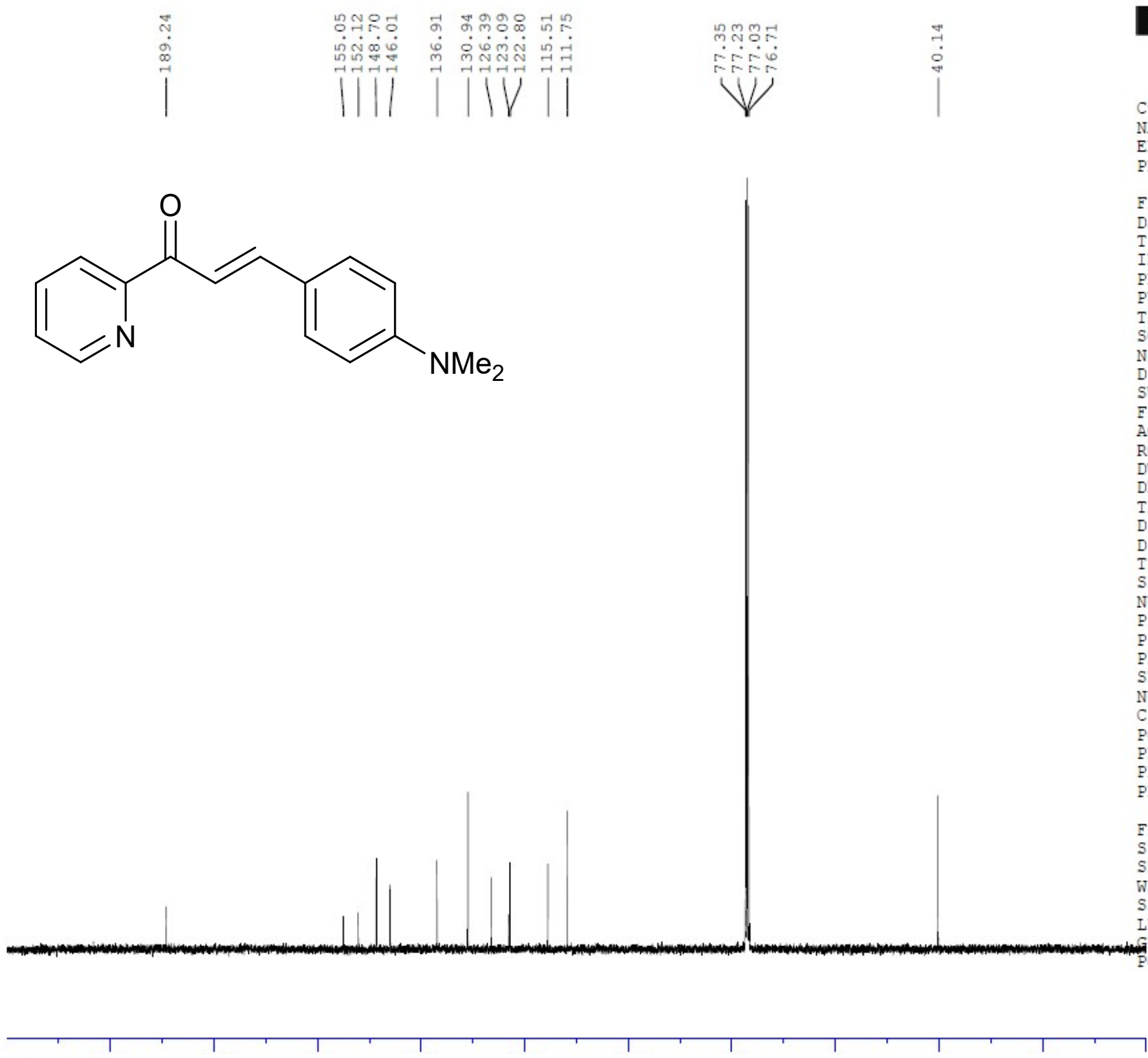

Current Data Parameters
NAME Apr14-2026-Alex
EXPNO 71
PROCNO 1

F2 - Acquisition Parameters
Date_ 20260414
Time 19.19 h
INSTRUM av400
PROBHD Z163739_1011 (
PULPROG zgpg30
TD 65536
SOLVENT CDCl3
NS 512
DS 4
SWH 23148.148 Hz
FIDRES 0.706425 Hz
AQ 1.4155777 sec
RG 209.43
DW 21.600 usec
DE 6.50 usec
TE 298.2 K
D1 2.00000000 sec
D11 0.03000000 sec
TD0 1
SFO1 100.6384215 MHz
NUC1 13C
P0 2.67 usec
P1 8.00 usec
PLW1 98.76699829 W
SFO2 400.1916008 MHz
NUC2 1H
CPDPRG[2 waltz16
PCPD2 90.00 usec
PLW2 22.76199913 W
PLW12 0.17985000 W
PLW13 0.09046300 W

F2 - Processing parameters
SI 32768
SF 100.6278555 MHz
WDW EM
SSB 0
LB 1.00 Hz
GB 0
PC 1.40

AC:37C
PROTON16.CMDnp CDCl3 {C:\Bruker\Topspin3.8.0} nmrsu 31

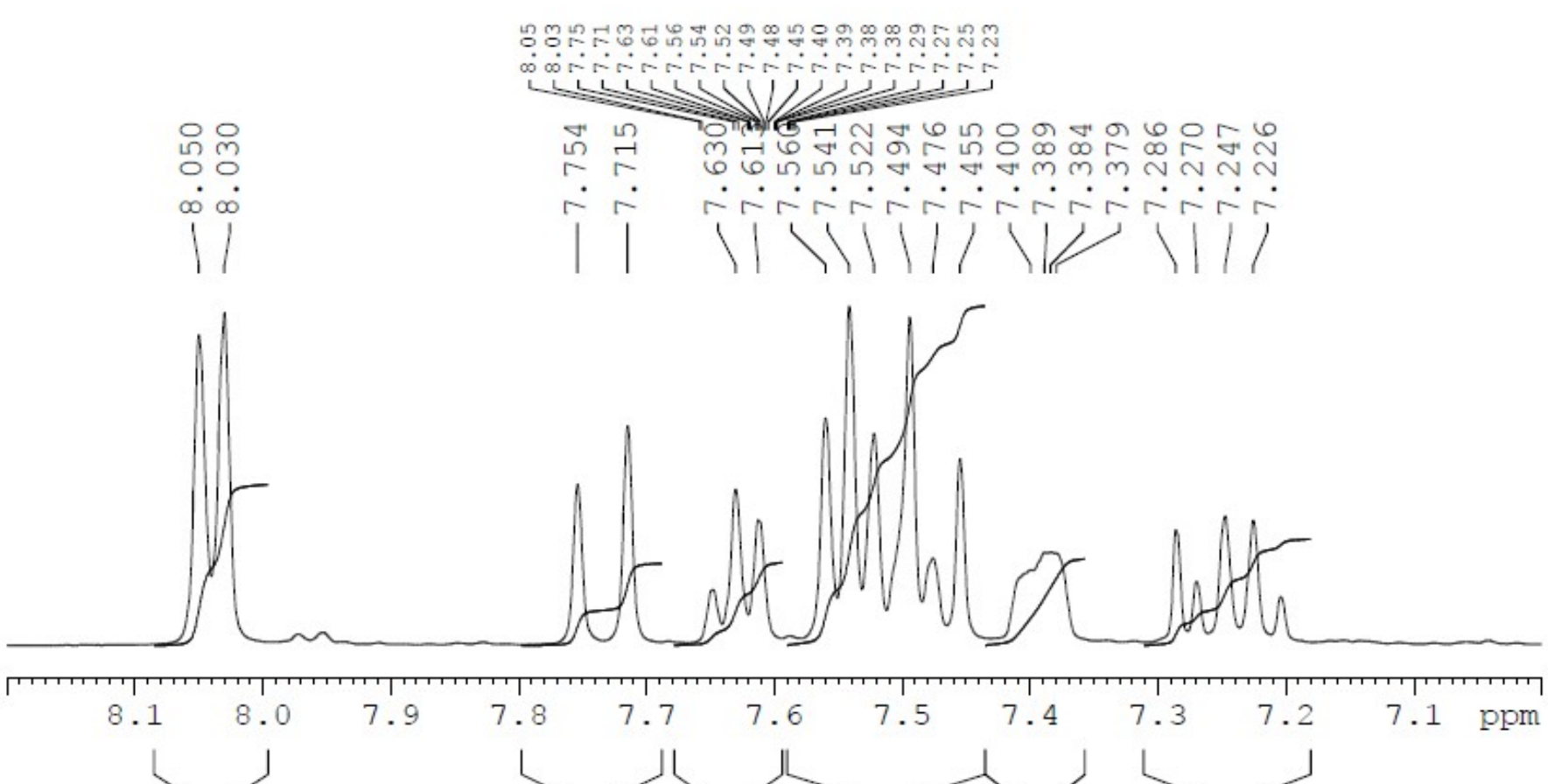

Current Data Parameters
NAME May08-2026-Alex
EXPNO 10
PROCNO 1

F2 - Acquisition Parameters
Date_ 20260508
Time 11.11 h
INSTRUM av400
PROBHD Z163739_1011 (
PULPROG zg30
TD 65536
SOLVENT CDCl3
NS 16
DS 2
SWH 6410.256 Hz
FIDRES 0.195625 Hz
AQ 5.1118078 sec
RG 104.12
DW 78.000 usec
DE 6.50 usec
TE 298.2 K
D1 1.00000000 sec
TD0 1
SFO1 400.1924011 MHz
NUC1 1H
P0 2.67 usec

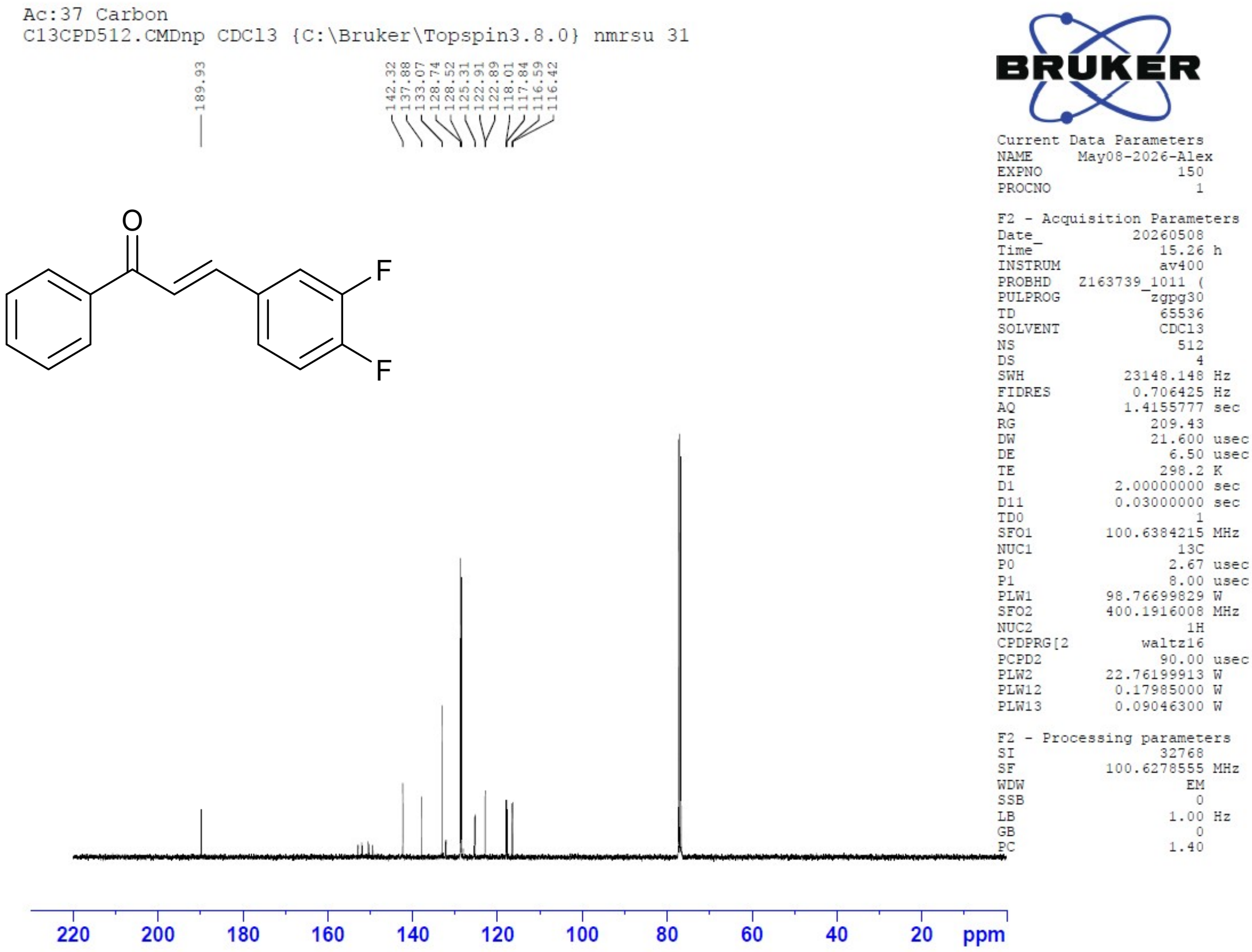
Ac:37 Carbon
C13CPD512.CMDnp CDCl3 {C:\Bruker\Topspin3.8.0} nmrsu 31
189.93
142.32
137.88
133.07
128.74
128.52
125.31
122.91
122.89
118.01
117.84
116.59
116.42
O
F
F
BRUKER
Current Data Parameters
NAME May08-2026-Alex
EXPNO 150
PROCNO 1
F2 - Acquisition Parameters
Date_ 20260508
Time 15.26 h
INSTRUM av400
PROBHD Z163739_1011 (
PULPROG zgpg30
TD 65536
SOLVENT CDCl3
NS 512
DS 4
SWH 23148.148 Hz
FIDRES 0.706425 Hz
AQ 1.4155777 sec
RG 209.43
DW 21.600 usec
DE 6.50 usec
TE 298.2 K
D1 2.00000000 sec
D11 0.03000000 sec
TD0 1
SFO1 100.6384215 MHz
NUC1 13C
P0 2.67 usec
P1 8.00 usec
PLW1 98.76699829 W
SFO2 400.1916008 MHz
NUC2 1H
CPDPRG[2 waltz16
PCPD2 90.00 usec
PLW2 22.76199913 W
PLW12 0.17985000 W
PLW13 0.09046300 W
F2 - Processing parameters
SI 32768
SF 100.6278555 MHz
WDW EM
SSB 0
LB 1.00 Hz
GB 0
PC 1.40
220 200 180 160 140 120 100 80 60 40 20 ppm

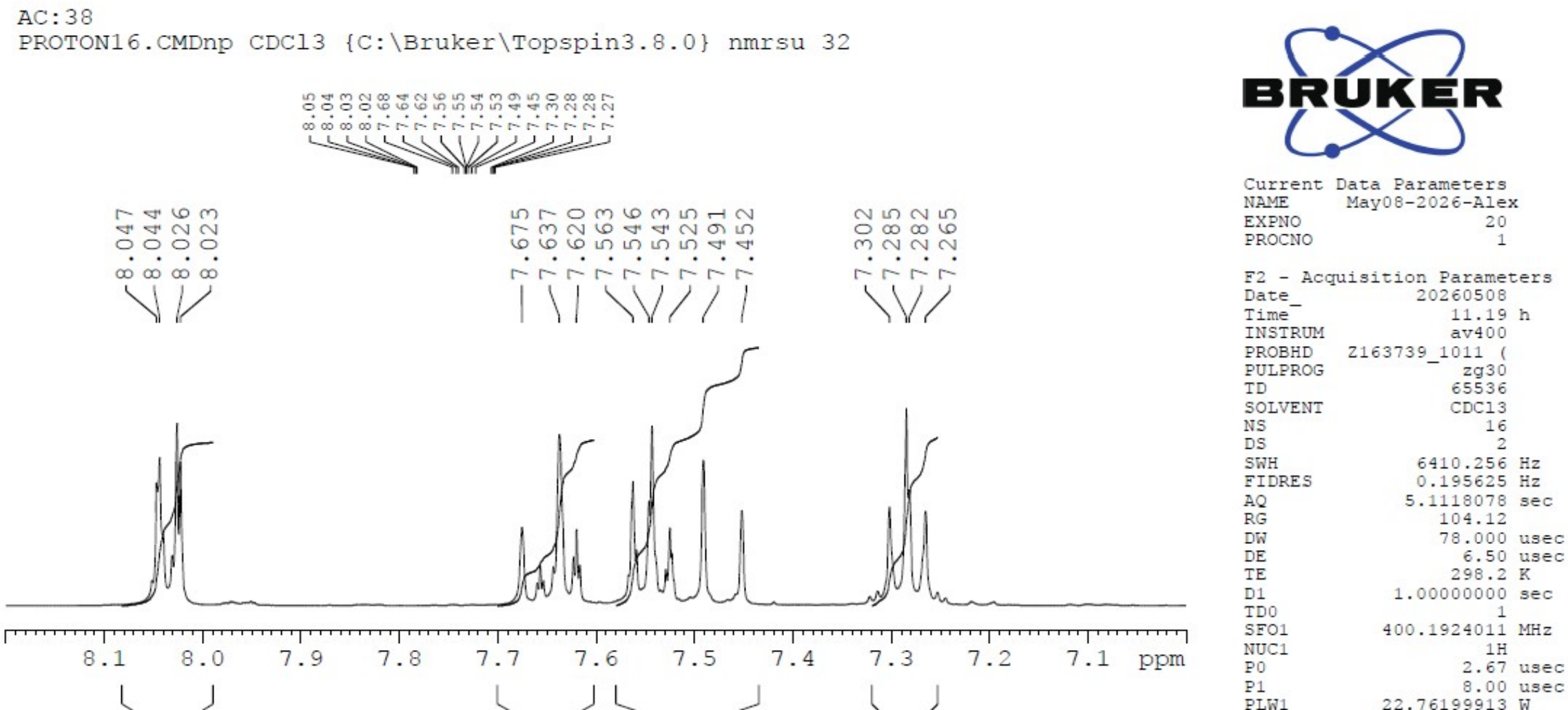
AC:38
PROTON16.CMDnp CDCl3 {C:\Bruker\Topspin3.8.0} nmrsu 32
8.05
8.04
8.03
8.02
7.68
7.64
7.62
7.56
7.55
7.54
7.53
7.49
7.45
7.30
7.28
7.28
7.27
8.047
8.044
8.026
8.023
7.675
7.637
7.620
7.563
7.546
7.543
7.525
7.491
7.452
7.302
7.285
7.282
7.265
BRUKER
Current Data Parameters
NAME May08-2026-Alex
EXPNO 20
PROCNO 1
F2 - Acquisition Parameters
Date_ 20260508
Time 11.19 h
INSTRUM av400
PROBHD Z163739_1011 (
PULPROG zg30
TD 65536
SOLVENT CDCl3
NS 16
DS 2
SWH 6410.256 Hz
FIDRES 0.195625 Hz
AQ 5.1118078 sec
RG 104.12
DW 78.000 usec
DE 6.50 usec
TE 298.2 K
D1 1.00000000 sec
TD0 1
SFO1 400.1924011 MHz
NUC1 1H
P0 2.67 usec
P1 8.00 usec
PLW1 22.76199913 W
8.1 8.0 7.9 7.8 7.7 7.6 7.5 7.4 7.3 7.2 7.1 ppm

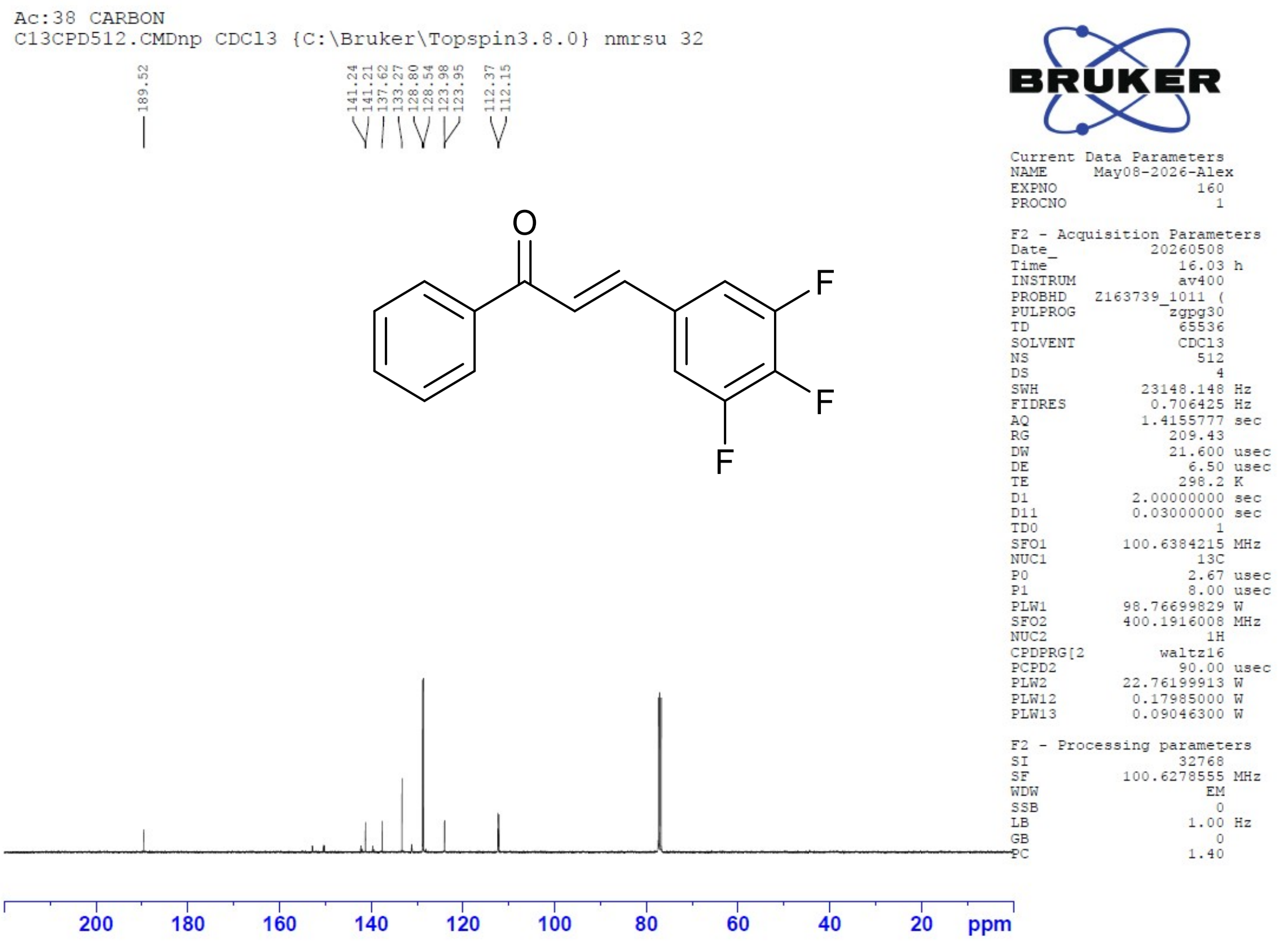
Ac:38 CARBON
C13CPD512.CMDnp CDCl3 {C:\Bruker\Topspin3.8.0} nmrsu 32
189.52
141.24
141.21
137.62
133.27
128.80
128.54
123.98
123.95
112.37
112.15
O
F
F
F
BRUKER
Current Data Parameters
NAME May08-2026-Alex
EXPNO 160
PROCNO 1
F2 - Acquisition Parameters
Date_ 20260508
Time 16.03 h
INSTRUM av400
PROBHD Z163739_1011 (
PULPROG zgpg30
TD 65536
SOLVENT CDCl3
NS 512
DS 4
SWH 23148.148 Hz
FIDRES 0.706425 Hz
AQ 1.4155777 sec
RG 209.43
DW 21.600 usec
DE 6.50 usec
TE 298.2 K
D1 2.00000000 sec
D11 0.03000000 sec
TD0 1
SFO1 100.6384215 MHz
NUC1 13C
P0 2.67 usec
P1 8.00 usec
PLW1 98.76699829 W
SFO2 400.1916008 MHz
NUC2 1H
CPDPRG[2 waltz16
PCPD2 90.00 usec
PLW2 22.76199913 W
PLW12 0.17985000 W
PLW13 0.09046300 W
F2 - Processing parameters
SI 32768
SF 100.6278555 MHz
WDW EM
SSB 0
LB 1.00 Hz
GB 0
PC 1.40
200
180
160
140
120
100
80
60
40
20
ppm

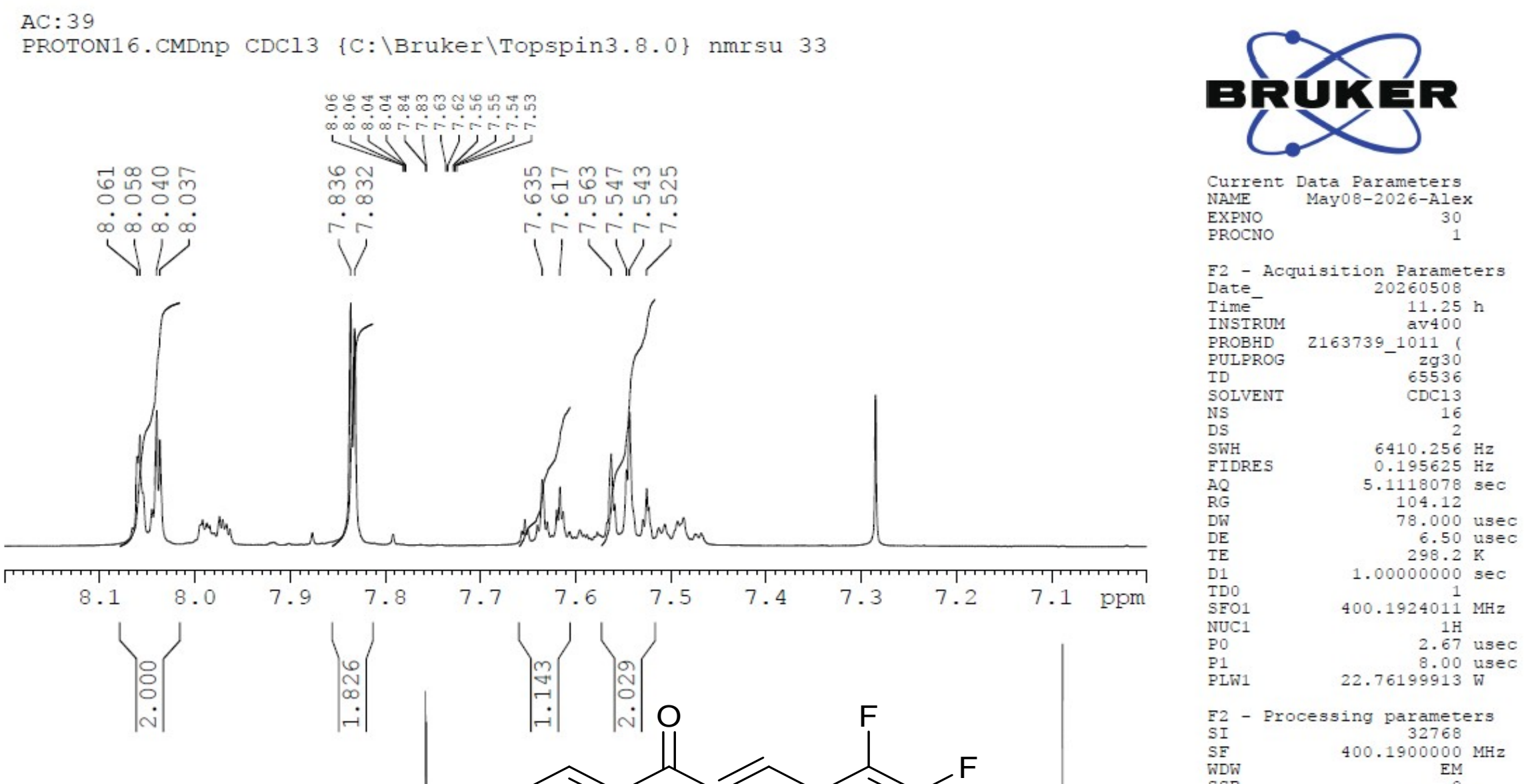
AC:39
PROTON16.CMDnp CDCl3 {C:\Bruker\Topspin3.8.0} nmrsu 33
8.06
8.06
8.04
8.04
7.84
7.83
7.63
7.62
7.56
7.55
7.54
7.53
8.061
8.058
8.040
8.037
7.836
7.832
7.635
7.617
7.563
7.547
7.543
7.525
8.1
8.0
7.9
7.8
7.7
7.6
7.5
7.4
7.3
7.2
7.1
ppm
2.000
1.826
1.143
2.029
O
F
F
BRUKER
Current Data Parameters
NAME May08-2026-Alex
EXPNO 30
PROCNO 1
F2 - Acquisition Parameters
Date_ 20260508
Time 11.25 h
INSTRUM av400
PROBHD Z163739_1011 (
PULPROG zg30
TD 65536
SOLVENT CDCl3
NS 16
DS 2
SWH 6410.256 Hz
FIDRES 0.195625 Hz
AQ 5.1118078 sec
RG 104.12
DW 78.000 usec
DE 6.50 usec
TE 298.2 K
D1 1.00000000 sec
TD0 1
SFO1 400.1924011 MHz
NUC1 1H
P0 2.67 usec
P1 8.00 usec
PLW1 22.76199913 W
F2 - Processing parameters
SI 32768
SF 400.1900000 MHz
WDW EM

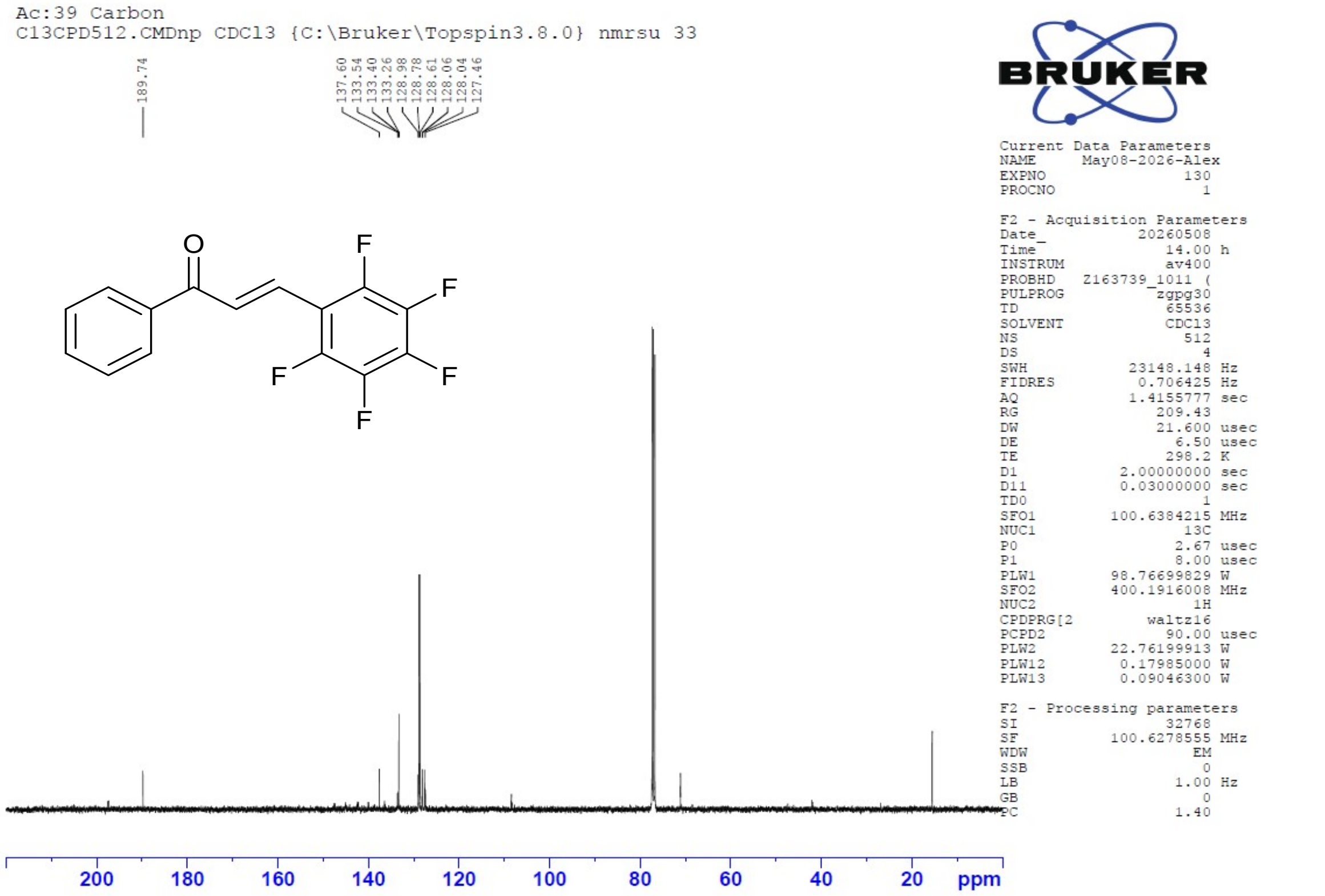
Ac:39 Carbon
C13CPD512.CMDnp CDCl3 {C:\Bruker\Topspin3.8.0} nmrsu 33
189.74
137.60
133.54
133.40
133.26
128.98
128.78
128.61
128.06
128.04
127.46
O
F
F
F
F
F
BRUKER
Current Data Parameters
NAME May08-2026-Alex
EXPNO 130
PROCNO 1
F2 - Acquisition Parameters
Date_ 20260508
Time 14.00 h
INSTRUM av400
PROBHD Z163739_1011 (
PULPROG zgpg30
TD 65536
SOLVENT CDCl3
NS 512
DS 4
SWH 23148.148 Hz
FIDRES 0.706425 Hz
AQ 1.4155777 sec
RG 209.43
DW 21.600 usec
DE 6.50 usec
TE 298.2 K
D1 2.00000000 sec
D11 0.03000000 sec
TD0 1
SFO1 100.6384215 MHz
NUC1 13C
P0 2.67 usec
P1 8.00 usec
PLW1 98.76699829 W
SFO2 400.1916008 MHz
NUC2 1H
CPDPRG[2 waltz16
PCPD2 90.00 usec
PLW2 22.76199913 W
PLW12 0.17985000 W
PLW13 0.09046300 W
F2 - Processing parameters
SI 32768
SF 100.6278555 MHz
WDW EM
SSB 0
LB 1.00 Hz
GB 0
PC 1.40
200
180
160
140
120
100
80
60
40
20
ppm

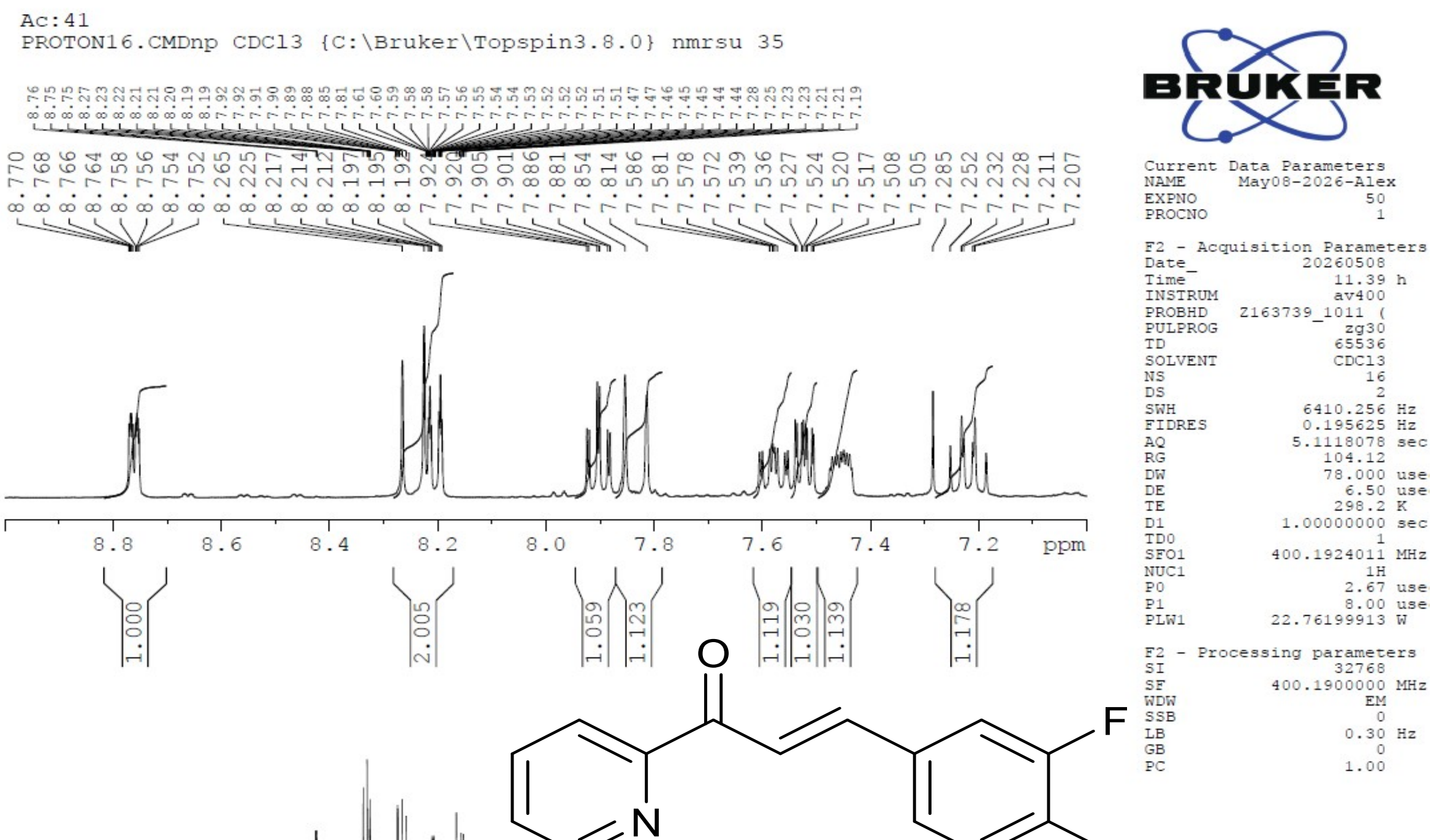
Ac:41
PROTON16.CMDnp CDCl3 {C:\Bruker\Topspin3.8.0} nmrsu 35
8.770
8.768
8.766
8.764
8.758
8.756
8.754
8.752
8.265
8.225
8.217
8.214
8.212
8.197
8.195
8.193
7.922
7.920
7.905
7.901
7.886
7.881
7.854
7.814
7.586
7.581
7.578
7.572
7.539
7.536
7.527
7.524
7.520
7.517
7.508
7.505
7.285
7.252
7.232
7.228
7.211
7.207
8.8
8.6
8.4
8.2
8.0
7.8
7.6
7.4
7.2
ppm
1.000
2.005
1.059
1.123
1.119
1.030
1.139
1.178
O
N
F
BRUKER
Current Data Parameters
NAME May08-2026-Alex
EXPNO 50
PROCNO 1
F2 - Acquisition Parameters
Date_ 20260508
Time 11.39 h
INSTRUM av400
PROBHD Z163739_1011 (
PULPROG zg30
TD 65536
SOLVENT CDCl3
NS 16
DS 2
SWH 6410.256 Hz
FIDRES 0.195625 Hz
AQ 5.1118078 sec
RG 104.12
DW 78.000 use
DE 6.50 use
TE 298.2 K
D1 1.00000000 sec
TD0 1
SFO1 400.1924011 MHz
NUC1 1H
P0 2.67 use
P1 8.00 use
PLW1 22.76199913 W
F2 - Processing parameters
SI 32768
SF 400.1900000 MHz
WDW EM
SSB 0
LB 0.30 Hz
GB 0
PC 1.00

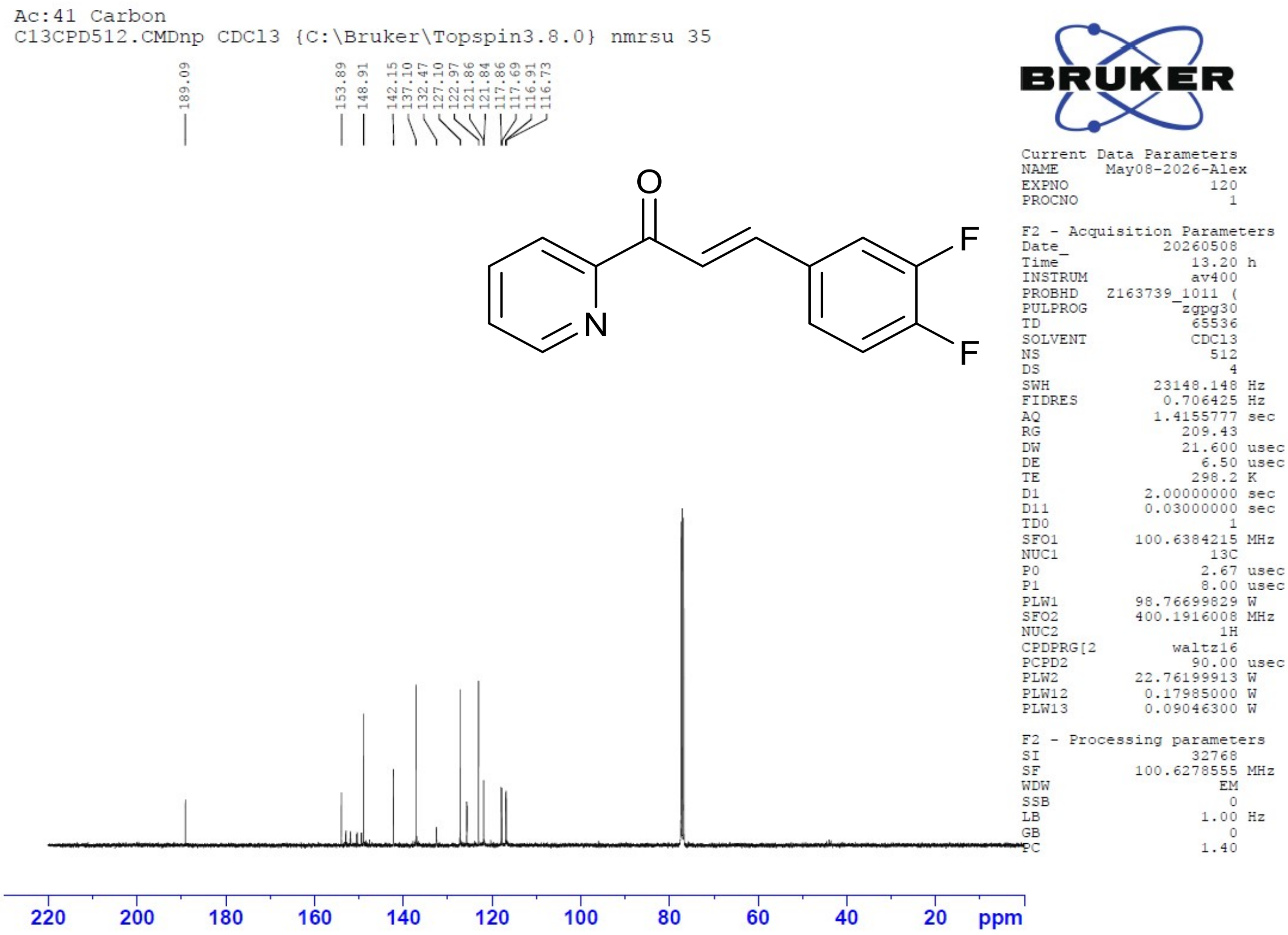
Ac:41 Carbon
C13CPD512.CMDnp CDCl3 {C:\Bruker\Topspin3.8.0} nmrsu 35
189.09
153.89
148.91
142.15
137.10
132.47
127.10
122.97
121.86
121.84
117.86
117.69
116.91
116.73
O
N
F
F
BRUKER
Current Data Parameters
NAME May08-2026-Alex
EXPNO 120
PROCNO 1
F2 - Acquisition Parameters
Date_ 20260508
Time 13.20 h
INSTRUM av400
PROBHD Z163739_1011 (
PULPROG zgpg30
TD 65536
SOLVENT CDCl3
NS 512
DS 4
SWH 23148.148 Hz
FIDRES 0.706425 Hz
AQ 1.4155777 sec
RG 209.43
DW 21.600 usec
DE 6.50 usec
TE 298.2 K
D1 2.00000000 sec
D11 0.03000000 sec
TD0 1
SFO1 100.6384215 MHz
NUC1 13C
P0 2.67 usec
P1 8.00 usec
PLW1 98.76699829 W
SFO2 400.1916008 MHz
NUC2 1H
CPDPRG[2 waltz16
PCPD2 90.00 usec
PLW2 22.76199913 W
PLW12 0.17985000 W
PLW13 0.09046300 W
F2 - Processing parameters
SI 32768
SF 100.6278555 MHz
WDW EM
SSB 0
LB 1.00 Hz
GB 0
PC 1.40
220 200 180 160 140 120 100 80 60 40 20 ppm

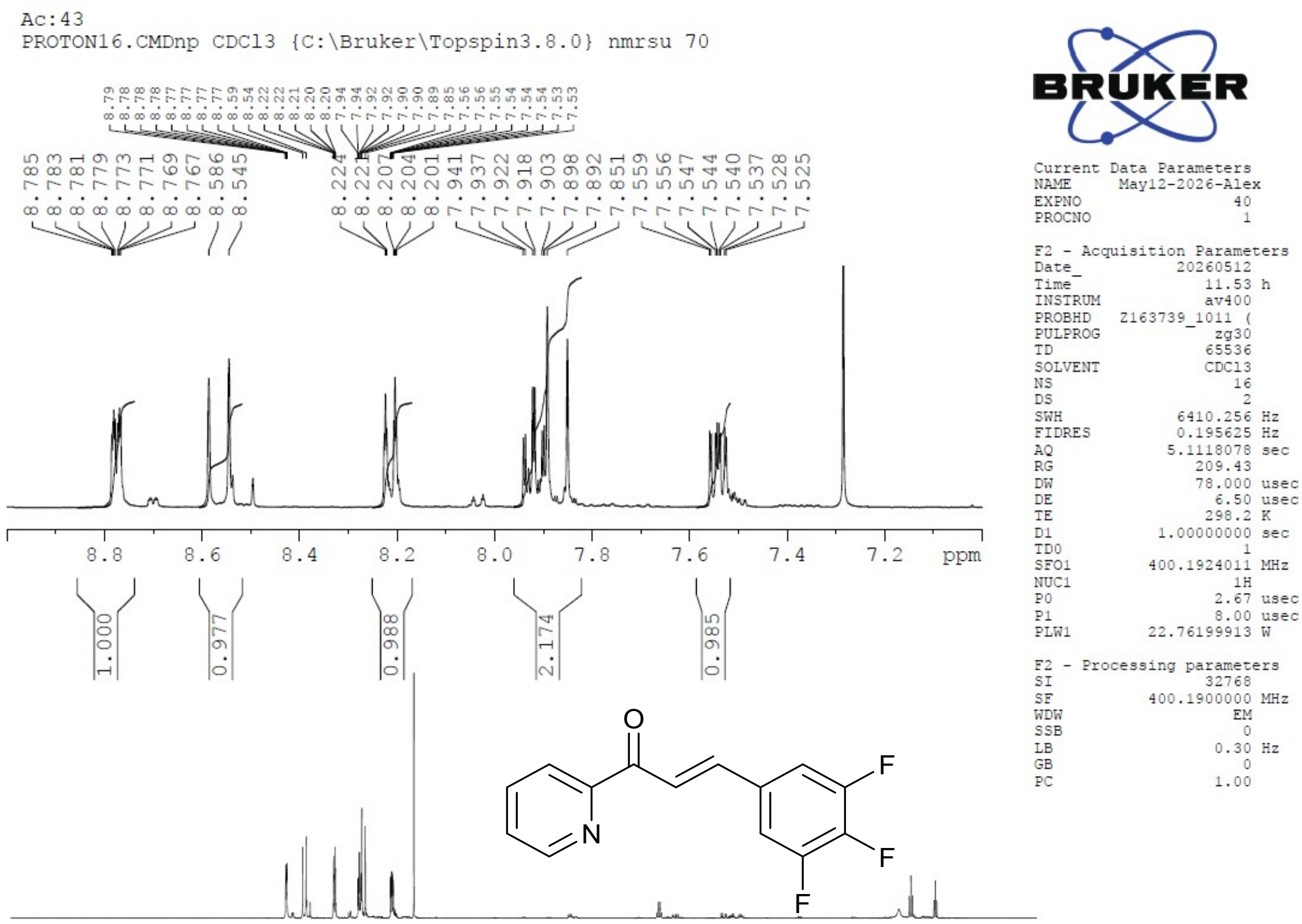
Ac:43
PROTON16.CMDnp CDCl3 {C:\Bruker\Topspin3.8.0} nmrsu 70
8.785
8.783
8.781
8.779
8.773
8.771
8.769
8.767
8.586
8.545
8.224
8.222
8.207
8.204
8.201
7.941
7.937
7.922
7.918
7.903
7.898
7.892
7.851
7.559
7.556
7.547
7.544
7.540
7.537
7.528
7.525
8.8 8.6 8.4 8.2 8.0 7.8 7.6 7.4 7.2 ppm
1.000
0.977
0.988
2.174
0.985
BRUKER
Current Data Parameters
NAME May12-2026-Alex
EXPNO 40
PROCNO 1
F2 - Acquisition Parameters
Date_ 20260512
Time 11.53 h
INSTRUM av400
PROBHD Z163739_1011 (
PULPROG zg30
TD 65536
SOLVENT CDCl3
NS 16
DS 2
SWH 6410.256 Hz
FIDRES 0.195625 Hz
AQ 5.1118078 sec
RG 209.43
DW 78.000 usec
DE 6.50 usec
TE 298.2 K
D1 1.00000000 sec
TD0 1
SFO1 400.1924011 MHz
NUC1 1H
P0 2.67 usec
P1 8.00 usec
PLW1 22.76199913 W
F2 - Processing parameters
SI 32768
SF 400.1900000 MHz
WDW EM
SSB 0
LB 0.30 Hz
GB 0
PC 1.00
O
N
F
F
F

Ac:43
C13CPD512.CMDnp CDCl3 {C:\Bruker\Topspin3.8.0} nmrsu 70

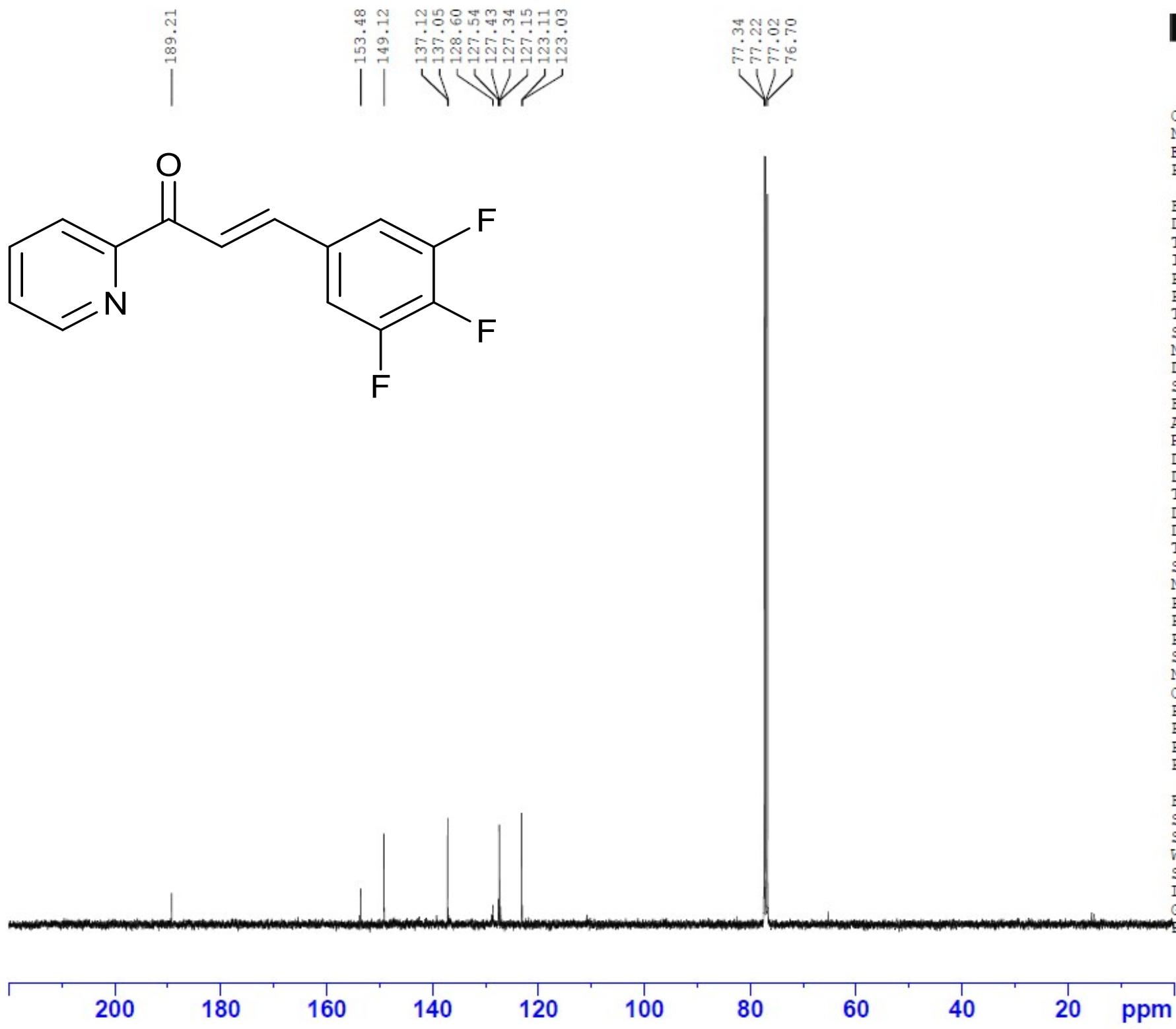

BRUKER

Current Data Parameters
NAME May12-2026-Alex
EXPNO 50
PROCNO 1

F2 - Acquisition Parameters
Date_ 20260512
Time 13.03 h
INSTRUM av400
PROBHD Z163739_1011 (
PULPROG zgpg30
TD 65536
SOLVENT CDCl3
NS 512
DS 4
SWH 23148.148 Hz
FIDRES 0.706425 Hz
AQ 1.4155777 sec
RG 209.43
DW 21.600 usec
DE 6.50 usec
TE 298.2 K
D1 2.00000000 sec
D11 0.03000000 sec
TD0 1
SFO1 100.6384215 MHz
NUC1 13C
P0 2.67 usec
P1 8.00 usec
PLW1 98.76699829 W
SFO2 400.1916008 MHz
NUC2 1H
CPDPRG[2 waltz16
PCPD2 90.00 usec
PLW2 22.76199913 W
PLW12 0.17985000 W
PLW13 0.09046300 W

F2 - Processing parameters
SI 32768
SF 100.6278555 MHz
WDW EM
SSB 0
LB 1.00 Hz
GB 0
PC 1.40

AC:45
PROTON16.CMDnp CDCl3 {C:\Bruker\Topspin3.8.0} nmrsu 53

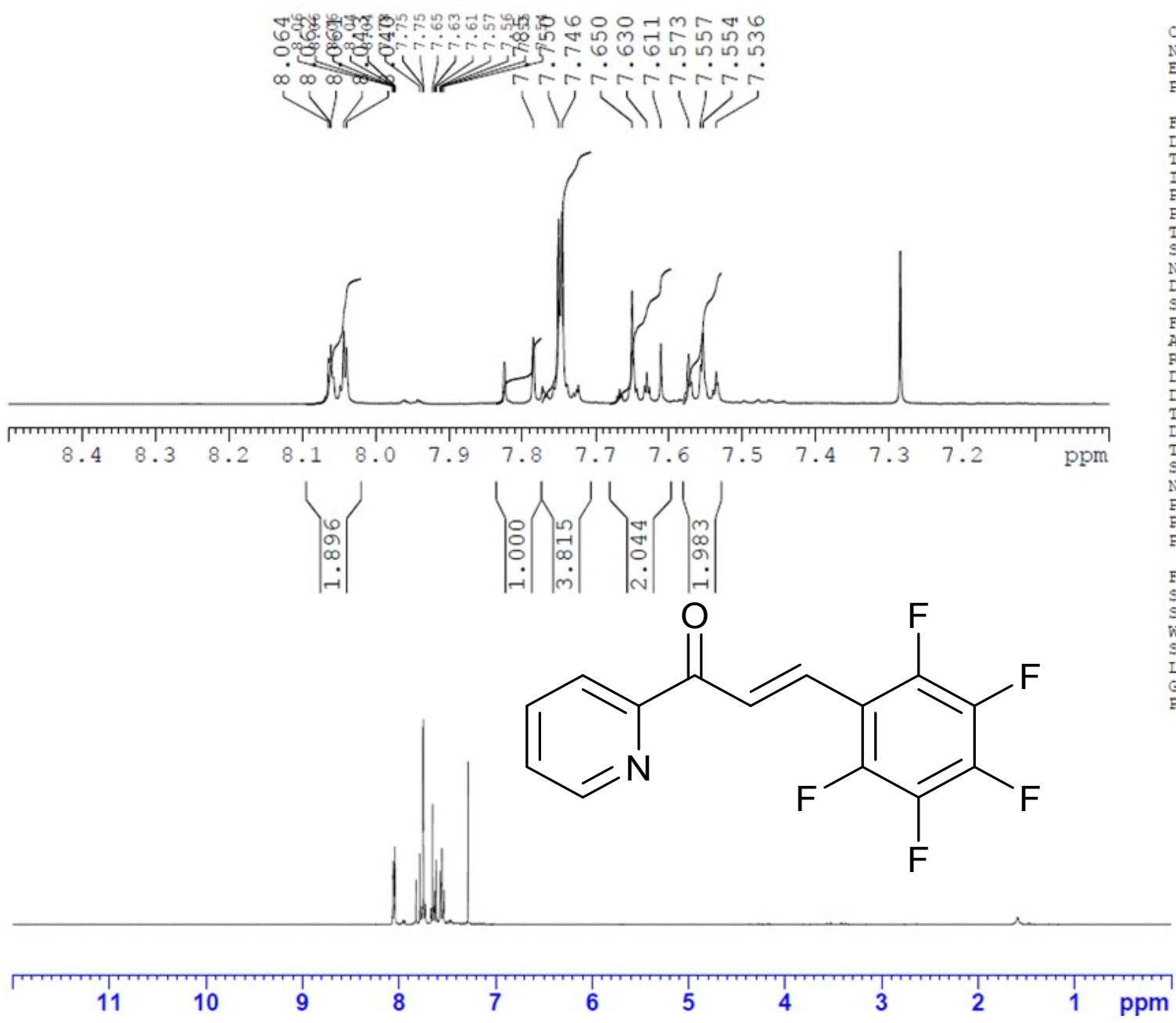

BRUKER

Current Data Parameters
NAME May11-2026-Alex
EXPNO 10
PROCNO 1

F2 - Acquisition Parameters
Date_ 20260511
Time 9.01 h
INSTRUM av400
PROBHD Z163739_1011 (
PULPROG zg30
TD 65536
SOLVENT CDCl3
NS 16
DS 2
SWH 6410.256 Hz
FIDRES 0.195625 Hz
AQ 5.1118078 sec
RG 209.43
DW 78.000 usec
DE 6.50 usec
TE 298.2 K
D1 1.00000000 sec
TD0 1
SFO1 400.1924011 MHz
NUC1 1H
P0 2.67 usec
P1 8.00 usec
PLW1 22.76199913 W

F2 - Processing parameters
SI 32768
SF 400.1900000 MHz
WDW EM
SSB 0
LB 0.30 Hz
GB 0
PC 1.00

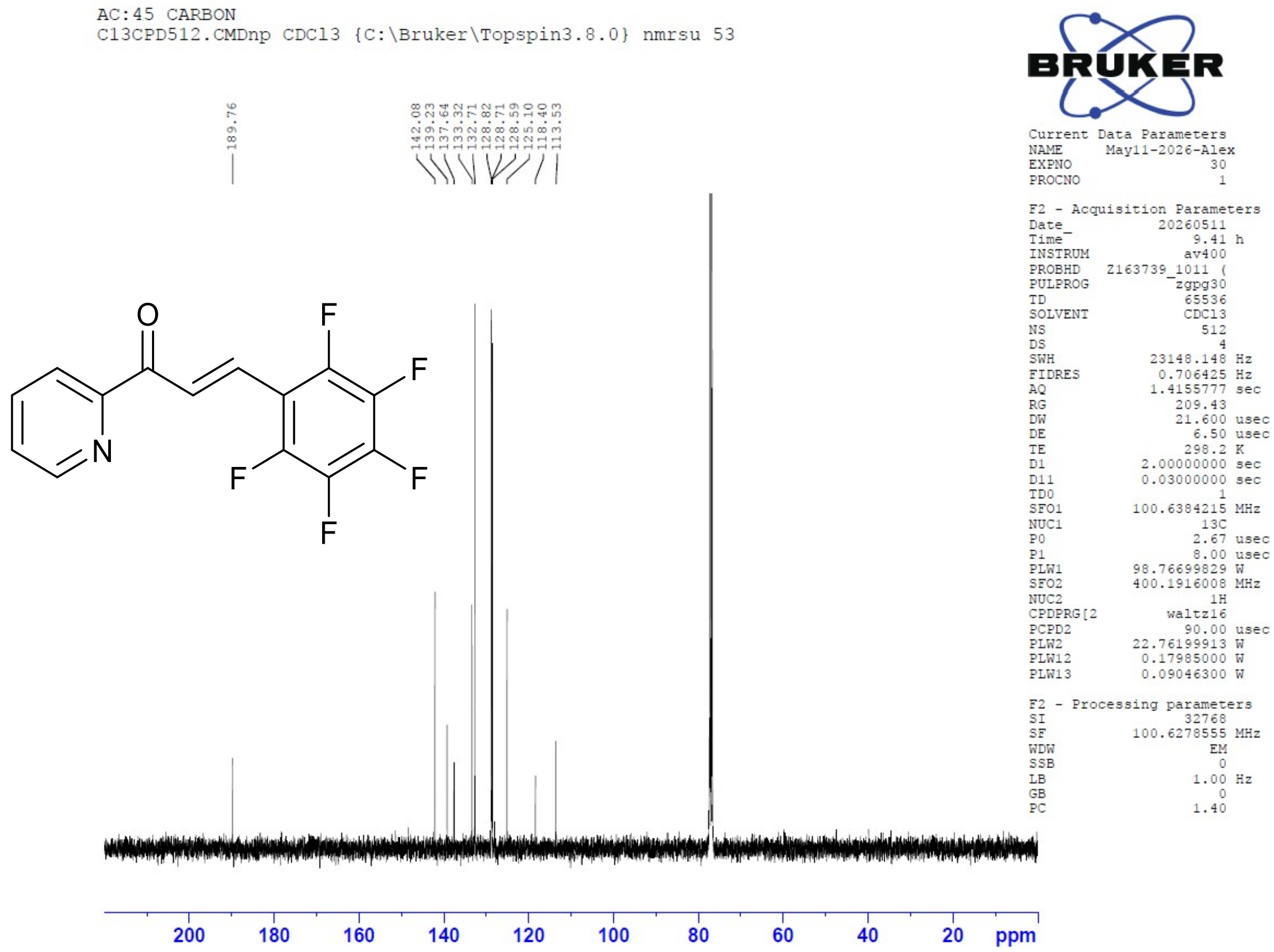

LC-MS to confirm Chalcone library purity (ESI S3)

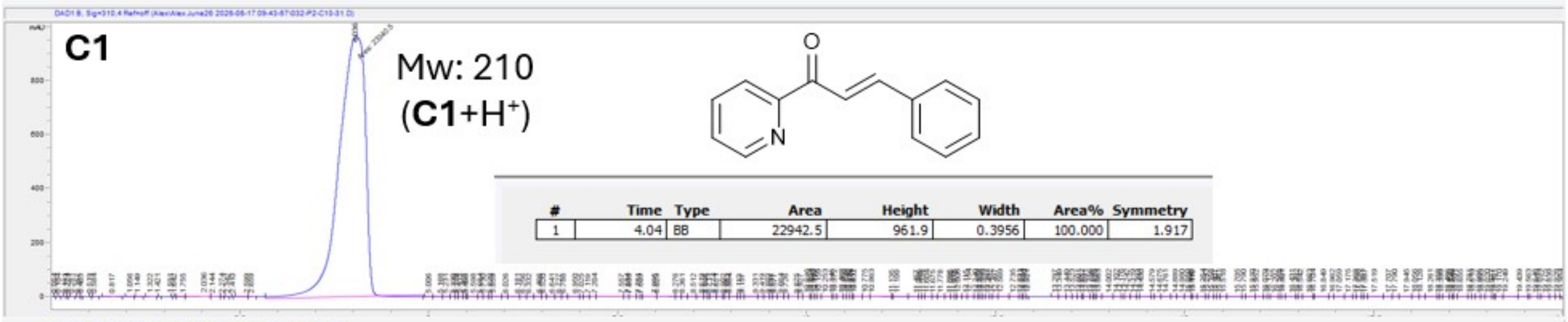

#	Time	Type	Area	Height	Width	Area%	Symmetry
1	4.04	BB	22942.5	961.9	0.3956	100.000	1.917

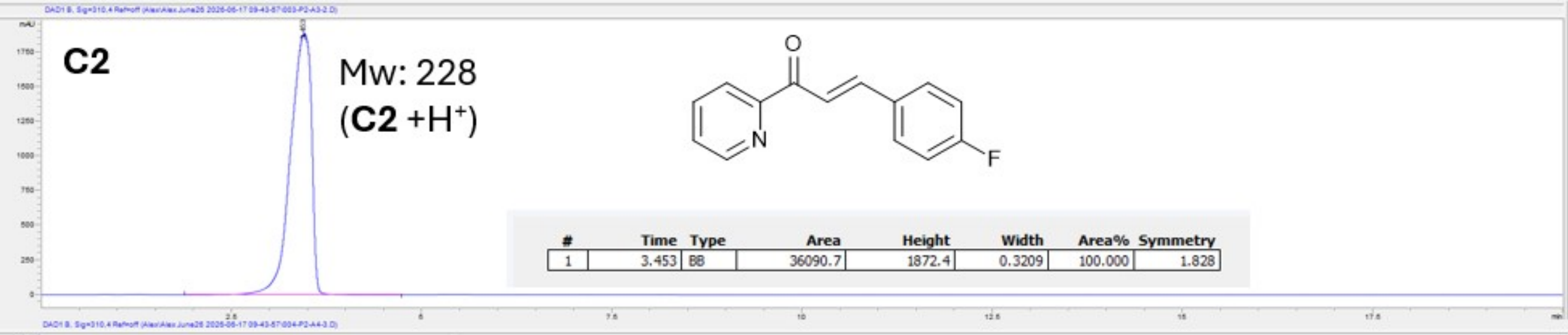

#	Time	Type	Area	Height	Width	Area%	Symmetry
1	3.453	BB	36090.7	1872.4	0.3209	100.000	1.828

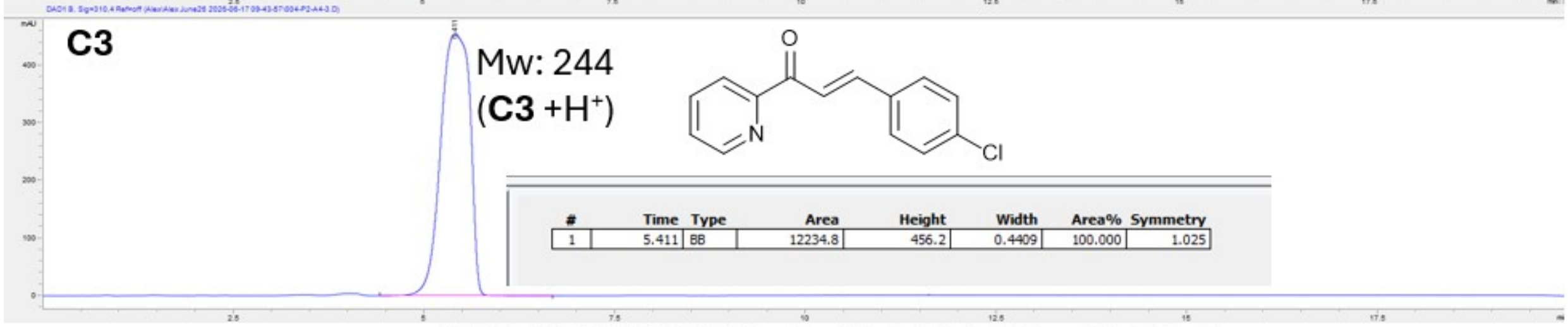

#	Time	Type	Area	Height	Width	Area%	Symmetry
1	5.411	BB	12234.8	456.2	0.4409	100.000	1.025

50% MeCN 50% H_2O (0.1% formic acid), 1mL/min, 310nm, 25uL injection ,

Poroshell C18 4um 4.6 x 100mm column

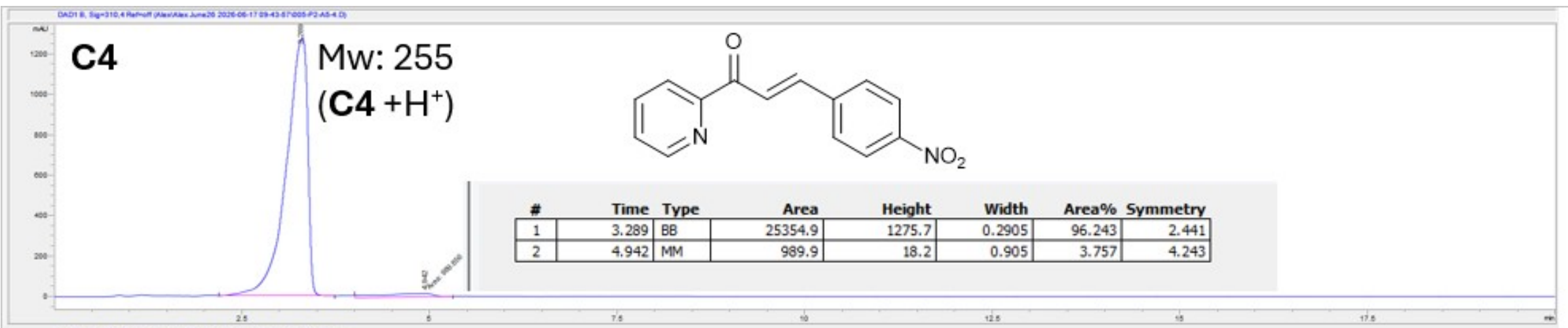

#	Time	Type	Area	Height	Width	Area%	Symmetry
1	3.289	BB	25354.9	1275.7	0.2905	96.243	2.441
2	4.942	MM	989.9	18.2	0.905	3.757	4.243

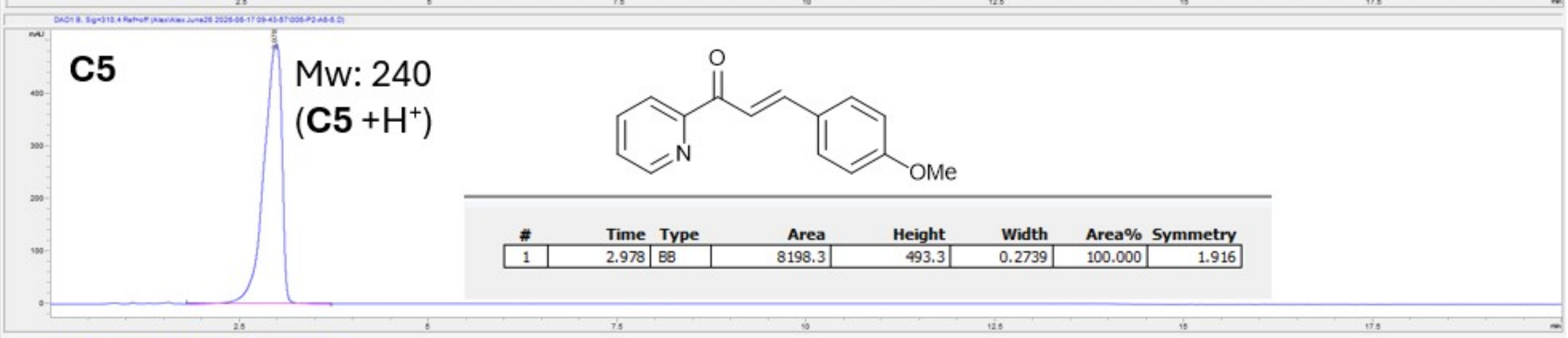

#	Time	Type	Area	Height	Width	Area%	Symmetry
1	2.978	BB	8198.3	493.3	0.2739	100.000	1.916

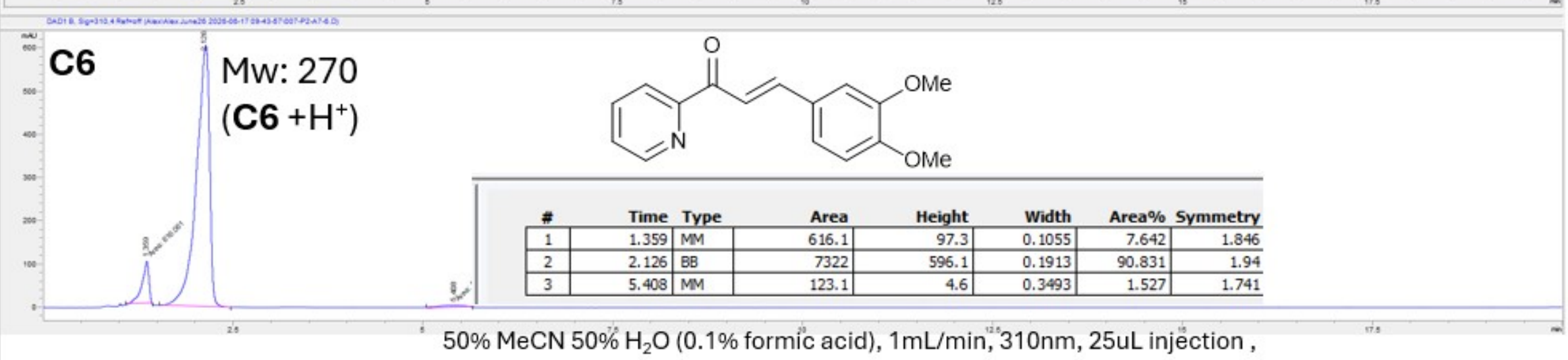

#	Time	Type	Area	Height	Width	Area%	Symmetry
1	1.359	MM	616.1	97.3	0.1055	7.642	1.846
2	2.126	BB	7322	596.1	0.1913	90.831	1.94
3	5.408	MM	123.1	4.6	0.3493	1.527	1.741

50% MeCN 50% H_2O (0.1% formic acid), 1mL/min, 310nm, 25uL injection ,

Poroshell C18 4um 4.6 x 100mm column

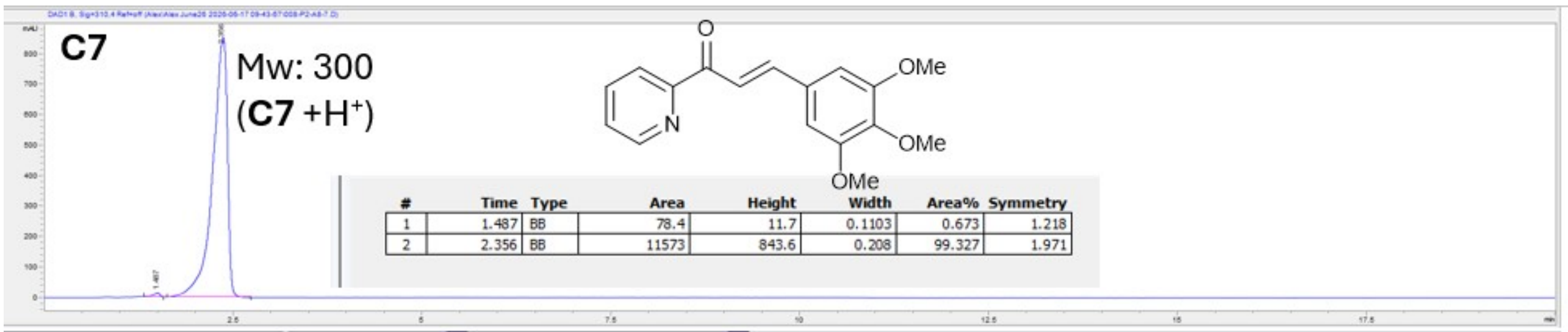

#	Time	Type	Area	Height	Width	Area%	Symmetry
1	1.487	BB	78.4	11.7	0.1103	0.673	1.218
2	2.356	BB	11573	843.6	0.208	99.327	1.971

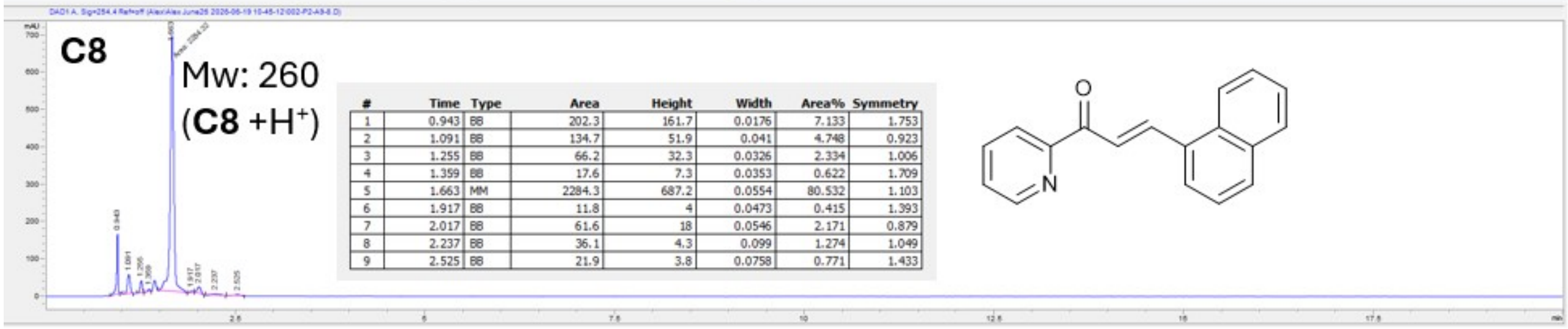

#	Time	Type	Area	Height	Width	Area%	Symmetry
1	0.943	BB	202.3	161.7	0.0176	7.133	1.753
2	1.091	BB	134.7	51.9	0.041	4.748	0.923
3	1.255	BB	66.2	32.3	0.0326	2.334	1.006
4	1.359	BB	17.6	7.3	0.0353	0.622	1.709
5	1.663	MM	2284.3	687.2	0.0554	80.532	1.103
6	1.917	BB	11.8	4	0.0473	0.415	1.393
7	2.017	BB	61.6	18	0.0546	2.171	0.879
8	2.237	BB	36.1	4.3	0.099	1.274	1.049
9	2.525	BB	21.9	3.8	0.0758	0.771	1.433

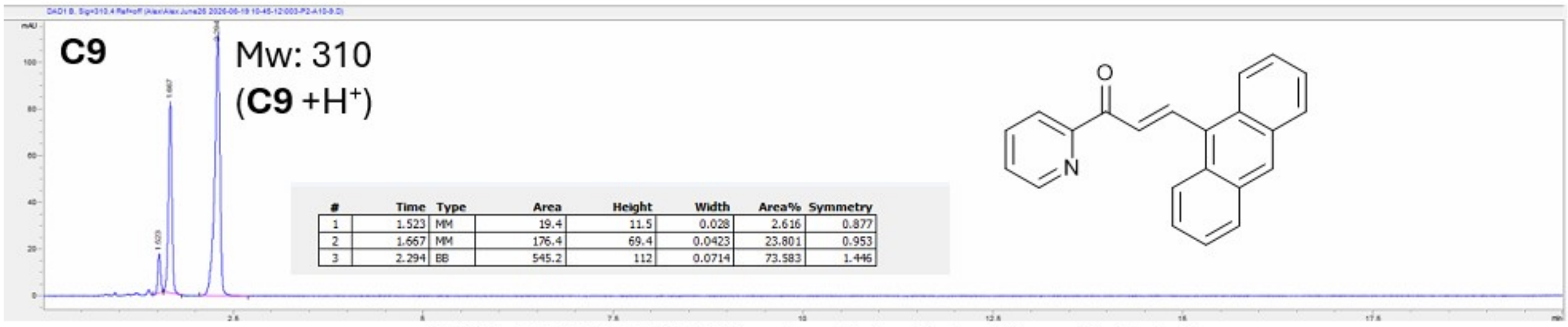

#	Time	Type	Area	Height	Width	Area%	Symmetry
1	1.523	MM	19.4	11.5	0.028	2.616	0.877
2	1.667	MM	176.4	69.4	0.0423	23.801	0.953
3	2.294	BB	545.2	112	0.0714	73.583	1.446

50% MeCN 50% H_2O (0.1% formic acid), 1mL/min, 310nm, 25uL injection ,

Poroshell C18 4um 4.6 x 100mm column

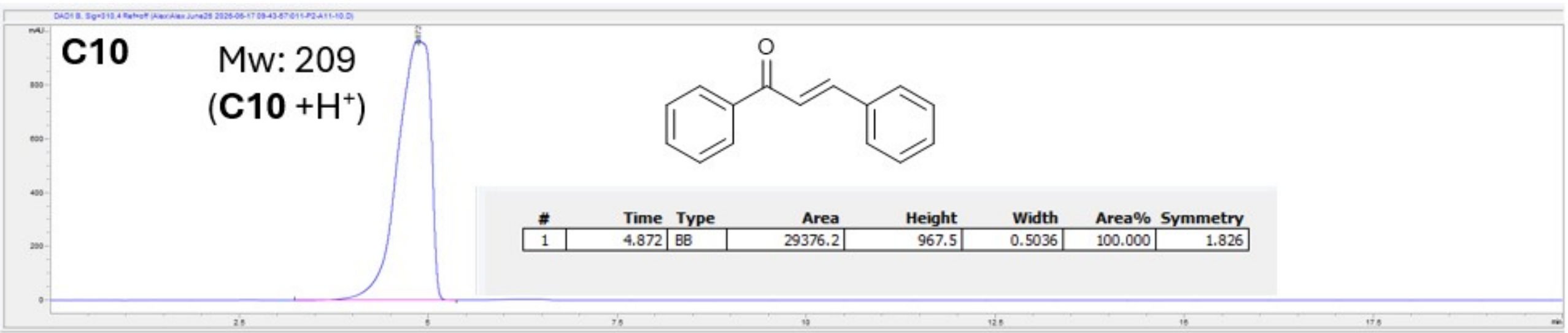

#	Time	Type	Area	Height	Width	Area%	Symmetry
1	4.872	BB	29376.2	967.5	0.5036	100.000	1.826

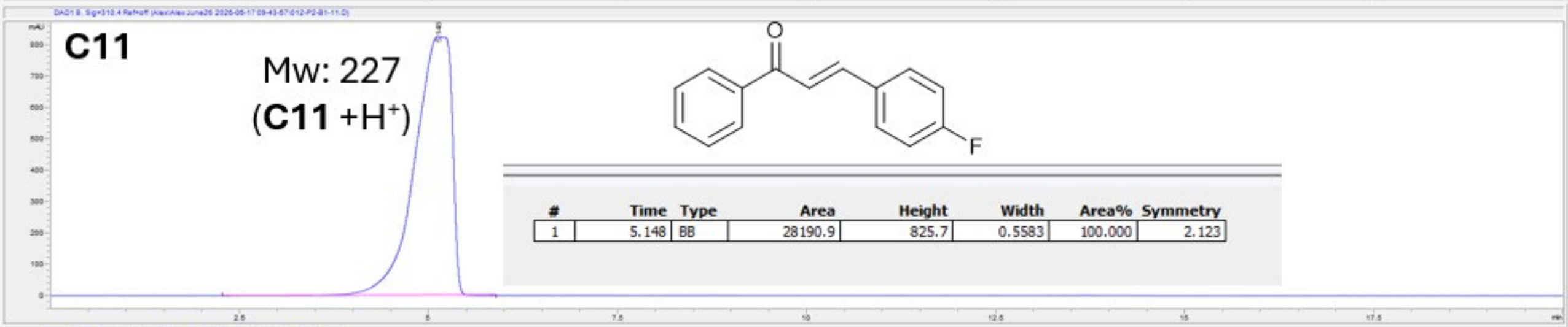

#	Time	Type	Area	Height	Width	Area%	Symmetry
1	5.148	BB	28190.9	825.7	0.5583	100.000	2.123

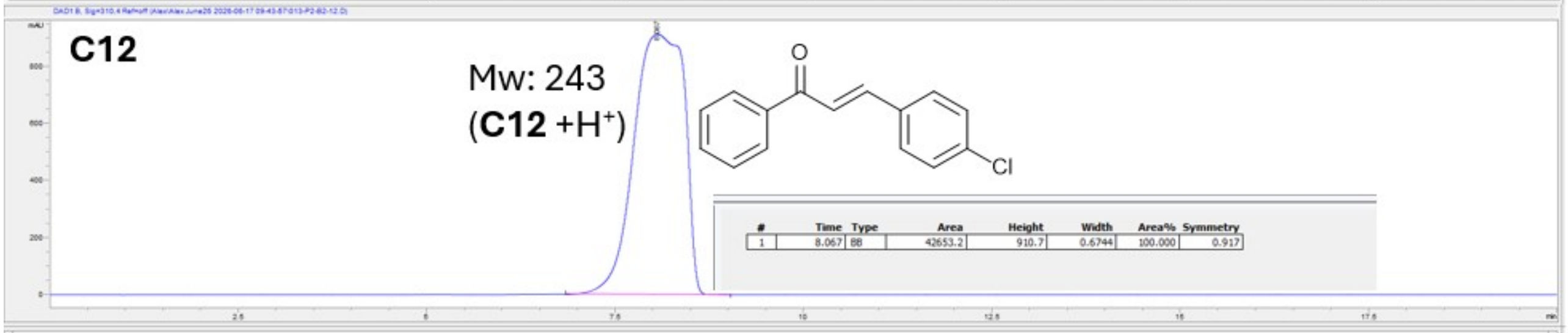

#	Time	Type	Area	Height	Width	Area%	Symmetry
1	8.067	BB	42653.2	910.7	0.6744	100.000	0.917

50% MeCN 50% H_2O (0.1% formic acid), 1mL/min, 310nm, 25uL injection ,

Poroshell C18 4um 4.6 x 100mm column

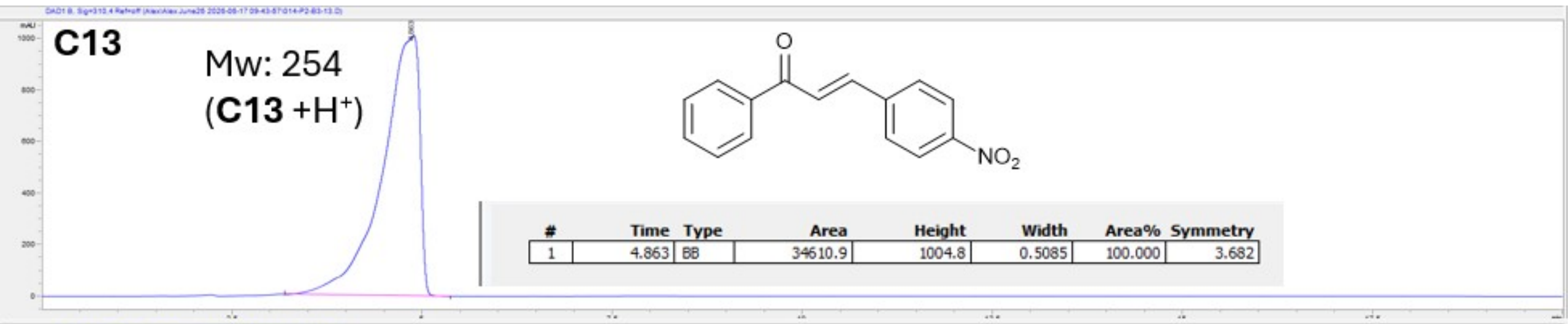

#	Time	Type	Area	Height	Width	Area%	Symmetry
1	4.863	BB	34610.9	1004.8	0.5085	100.000	3.682

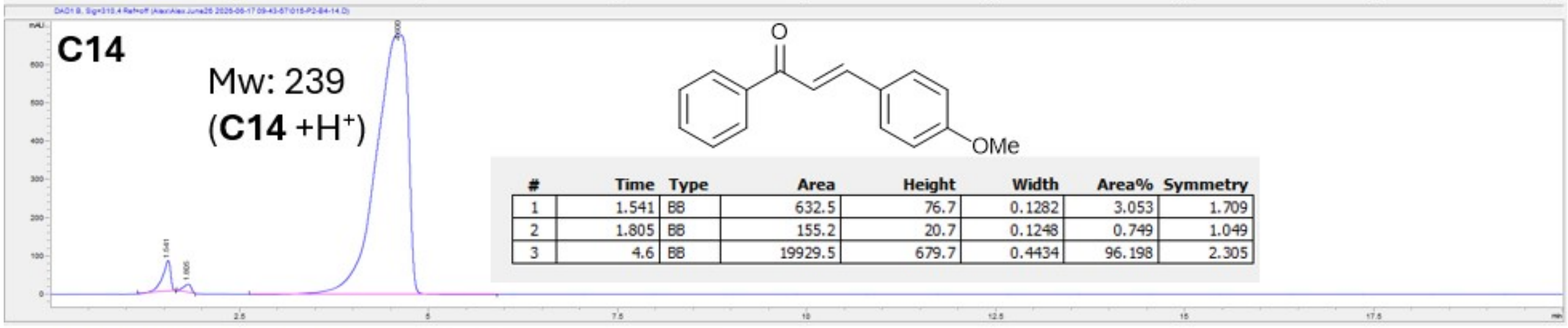

#	Time	Type	Area	Height	Width	Area%	Symmetry
1	1.541	BB	632.5	76.7	0.1282	3.053	1.709
2	1.805	BB	155.2	20.7	0.1248	0.749	1.049
3	4.6	BB	19929.5	679.7	0.4434	96.198	2.305

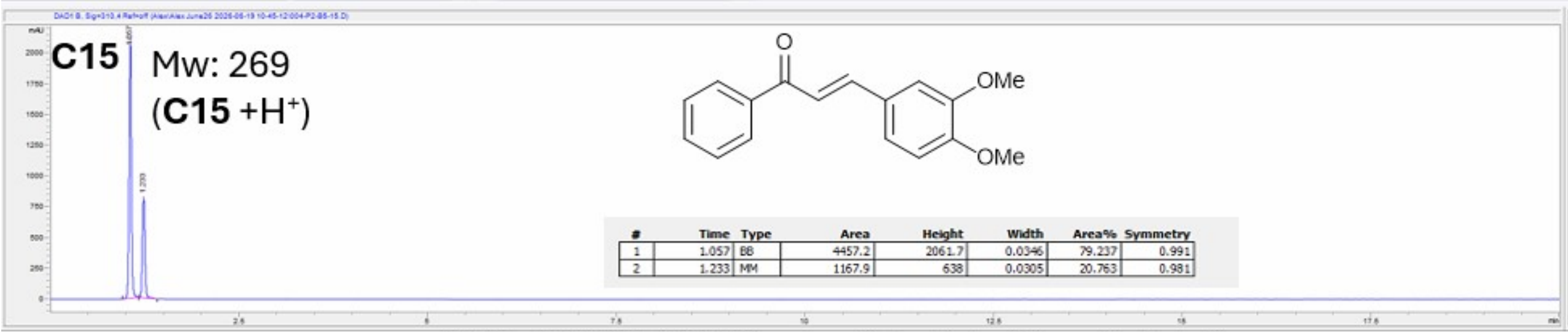

#	Time	Type	Area	Height	Width	Area%	Symmetry
1	1.057	BB	4457.2	2061.7	0.0346	79.237	0.991
2	1.233	MM	1167.9	638	0.0305	20.763	0.981

50% MeCN 50% H_2O (0.1% formic acid), 1mL/min, 310nm, 25uL injection ,

Poroshell C18 4um 4.6 x 100mm column

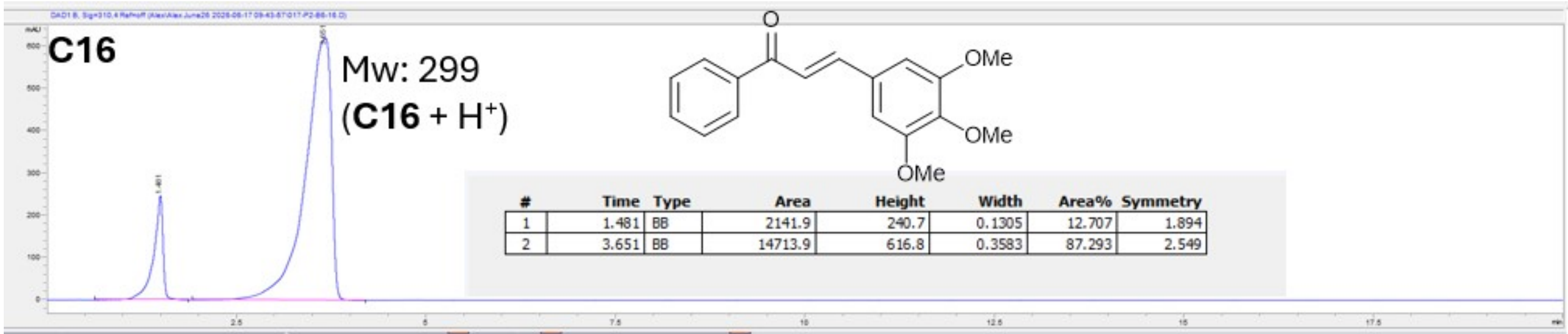

#	Time	Type	Area	Height	Width	Area%	Symmetry
1	1.481	BB	2141.9	240.7	0.1305	12.707	1.894
2	3.651	BB	14713.9	616.8	0.3583	87.293	2.549

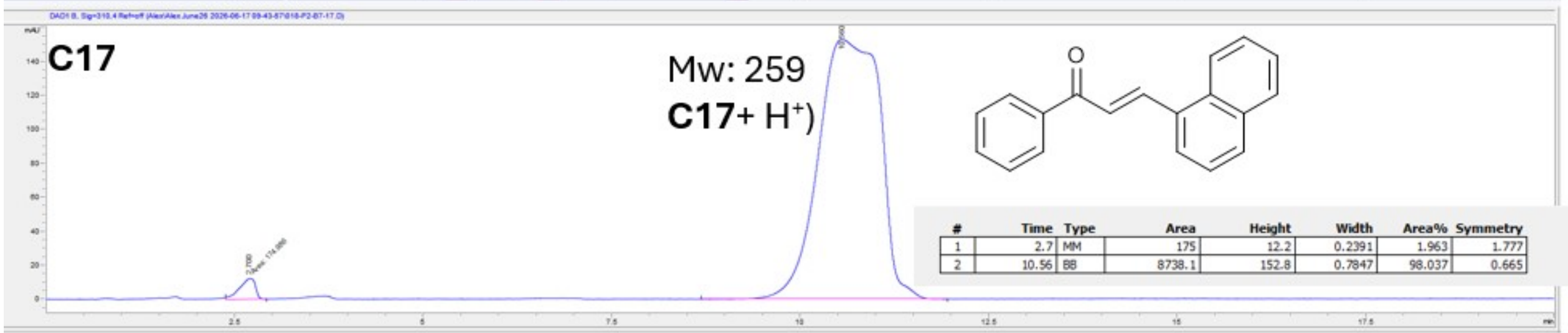

#	Time	Type	Area	Height	Width	Area%	Symmetry
1	2.7	MM	175	12.2	0.2391	1.963	1.777
2	10.56	BB	8738.1	152.8	0.7847	98.037	0.665

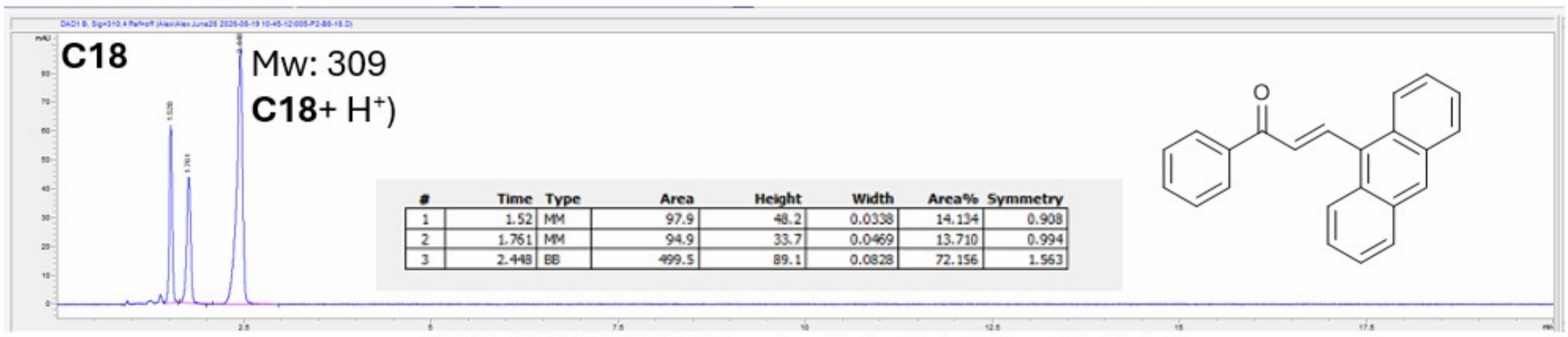

#	Time	Type	Area	Height	Width	Area%	Symmetry
1	1.52	MM	97.9	48.2	0.0338	14.134	0.908
2	1.761	MM	94.9	33.7	0.0469	13.710	0.994
3	2.448	BB	499.5	89.1	0.0828	72.156	1.563

50% MeCN 50% H_2O (0.1% formic acid), 1mL/min, 310nm, 25uL injection ,

Poroshell C18 4um 4.6 x 100mm column

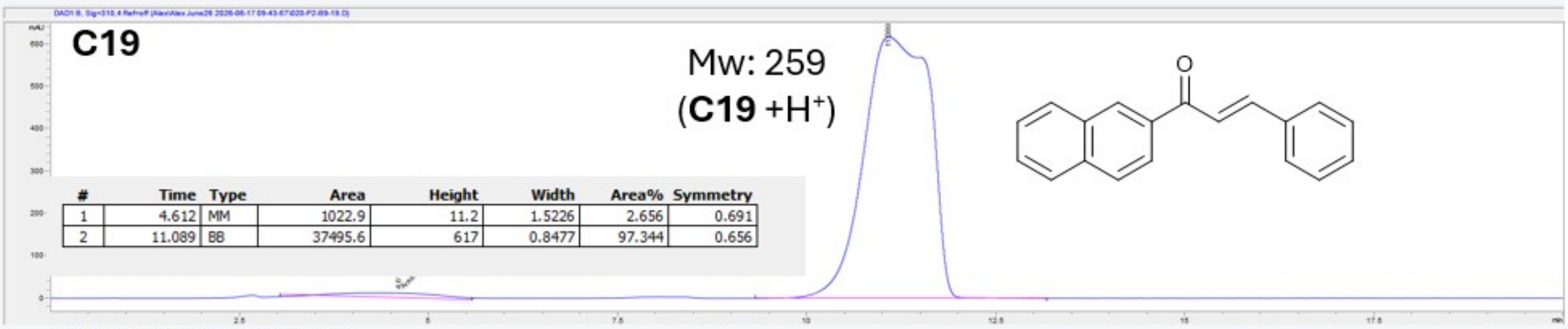

#	Time	Type	Area	Height	Width	Area%	Symmetry
1	4.612	MM	1022.9	11.2	1.5226	2.656	0.691
2	11.089	BB	37495.6	617	0.8477	97.344	0.656

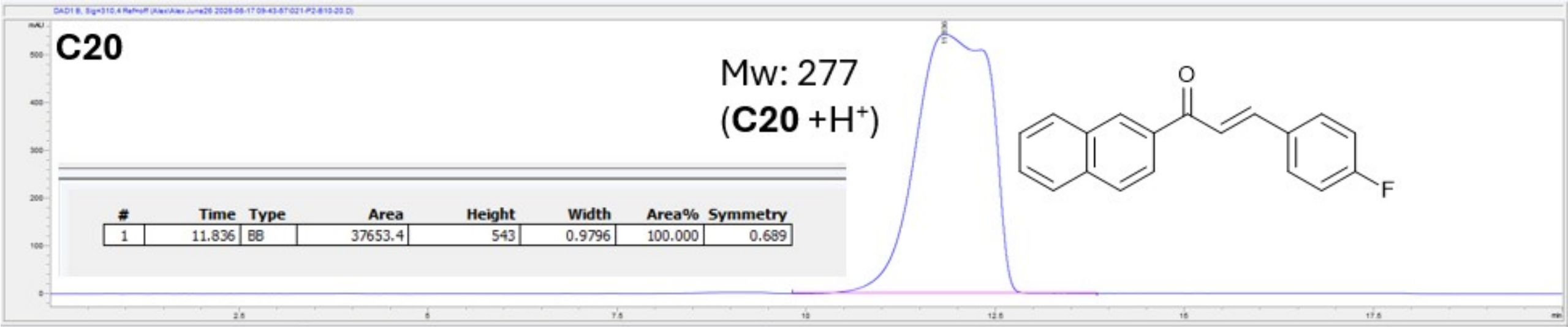

#	Time	Type	Area	Height	Width	Area%	Symmetry
1	11.836	BB	37653.4	543	0.9796	100.000	0.689

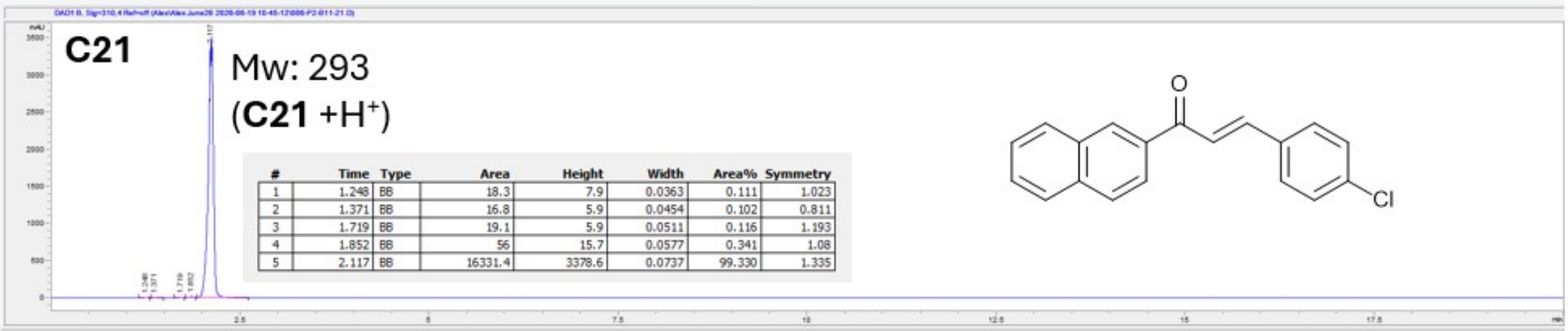

#	Time	Type	Area	Height	Width	Area%	Symmetry
1	1.248	BB	18.3	7.9	0.0363	0.111	1.023
2	1.371	BB	16.8	5.9	0.0454	0.102	0.811
3	1.719	BB	19.1	5.9	0.0511	0.116	1.193
4	1.852	BB	56	15.7	0.0577	0.341	1.08
5	2.117	BB	16331.4	3378.6	0.0737	99.330	1.335

50% MeCN 50% H_2O (0.1% formic acid), 1mL/min, 310nm, 25uL injection ,

Poroshell C18 4um 4.6 x 100mm column

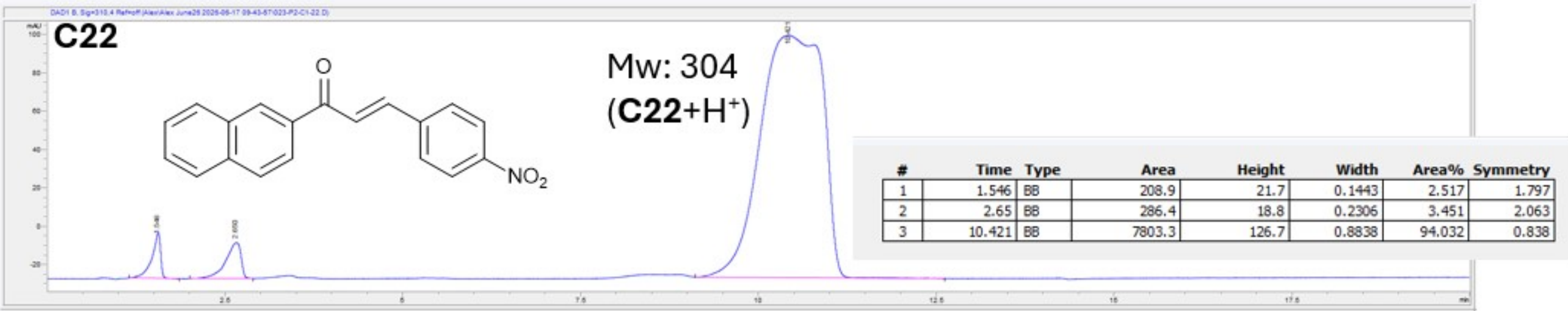

#	Time	Type	Area	Height	Width	Area%	Symmetry
1	1.546	BB	208.9	21.7	0.1443	2.517	1.797
2	2.65	BB	286.4	18.8	0.2306	3.451	2.063
3	10.421	BB	7803.3	126.7	0.8838	94.032	0.838

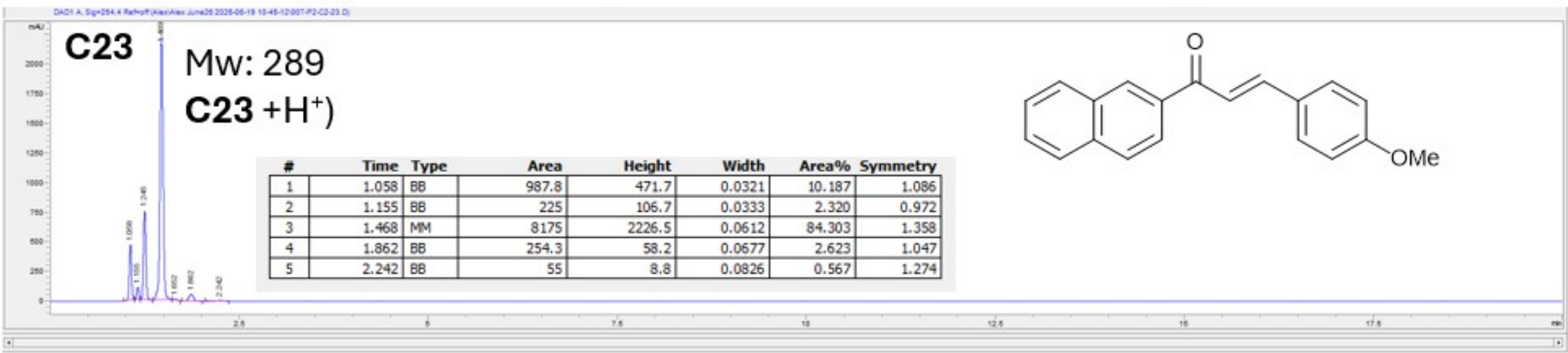

#	Time	Type	Area	Height	Width	Area%	Symmetry
1	1.058	BB	987.8	471.7	0.0321	10.187	1.086
2	1.155	BB	225	106.7	0.0333	2.320	0.972
3	1.468	MM	8175	2226.5	0.0612	84.303	1.358
4	1.862	BB	254.3	58.2	0.0677	2.623	1.047
5	2.242	BB	55	8.8	0.0826	0.567	1.274

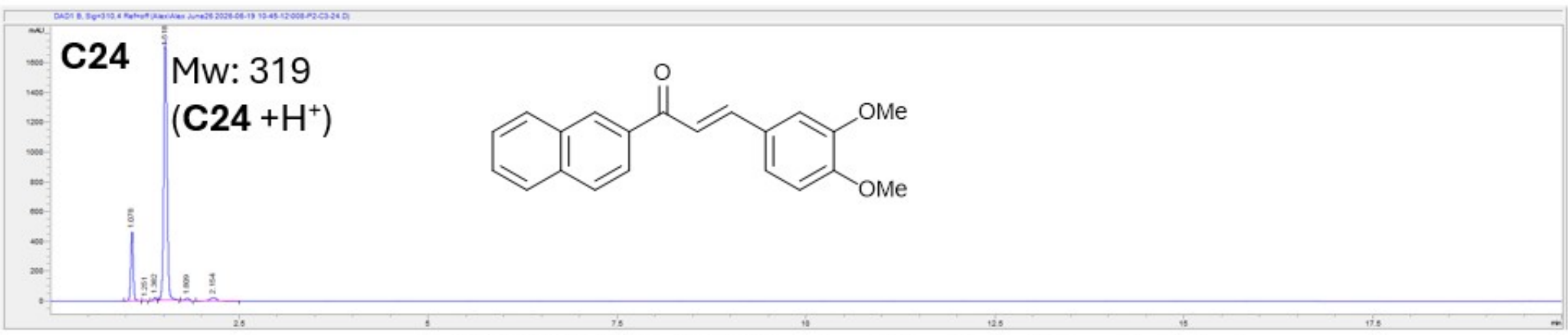

50% MeCN 50% H_2O (0.1% formic acid), 1mL/min, 310nm, 25uL injection ,

Poroshell C18 4um 4.6 x 100mm column

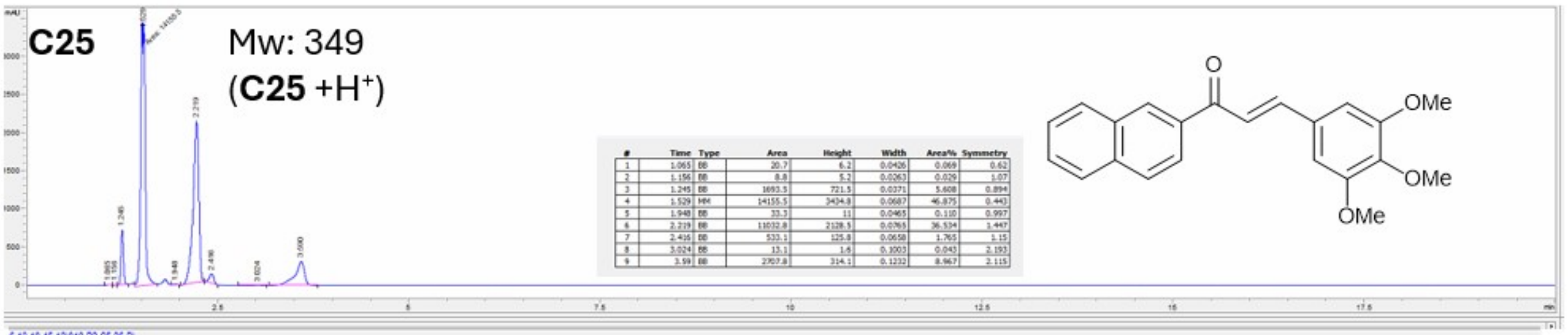

#	Time	Type	Area	Height	Width	Area%	Symmetry
1	1.065	BB	20.7	6.2	0.0426	0.069	0.62
2	1.156	BB	8.8	5.2	0.0263	0.029	1.07
3	1.245	BB	1693.5	721.5	0.0371	5.608	0.894
4	1.529	MM	14155.5	3434.8	0.0687	46.875	0.443
5	1.948	BB	33.3	11	0.0465	0.110	0.997
6	2.219	BB	11032.8	2128.5	0.0765	36.534	1.447
7	2.416	BB	533.1	125.8	0.0658	1.765	1.15
8	3.024	BB	13.1	1.6	0.1003	0.043	2.193
9	3.59	BB	2707.8	314.1	0.1232	8.967	2.115

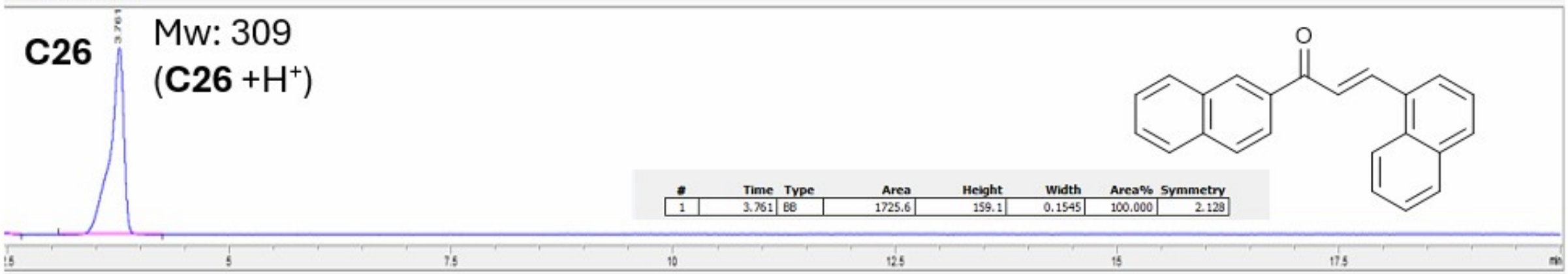

#	Time	Type	Area	Height	Width	Area%	Symmetry
1	3.761	BB	1725.6	159.1	0.1545	100.000	2.128

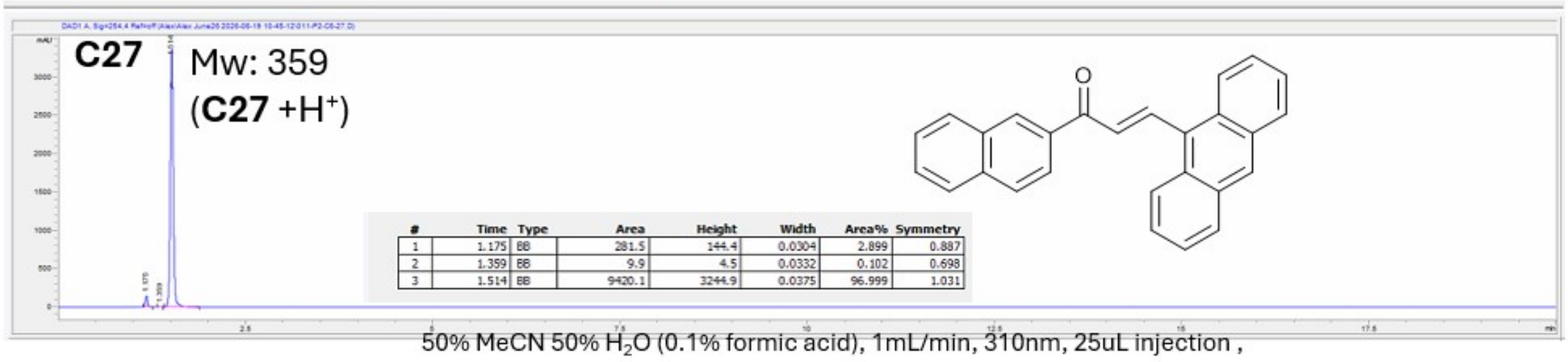

#	Time	Type	Area	Height	Width	Area%	Symmetry
1	1.175	BB	281.5	144.4	0.0304	2.899	0.887
2	1.359	BB	9.9	4.5	0.0332	0.102	0.698
3	1.514	BB	9420.1	3244.9	0.0375	96.999	1.031

50% MeCN 50% H_2O (0.1% formic acid), 1mL/min, 310nm, 25uL injection ,

Poroshell C18 4um 4.6 x 100mm column

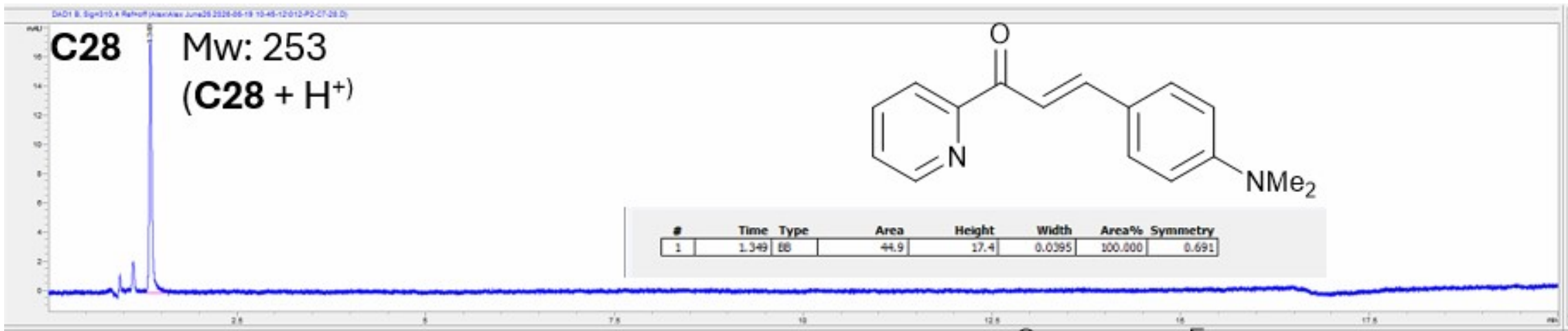

#	Time	Type	Area	Height	Width	Area%	Symmetry
1	1.349	BB	44.9	17.4	0.0395	100.000	0.691

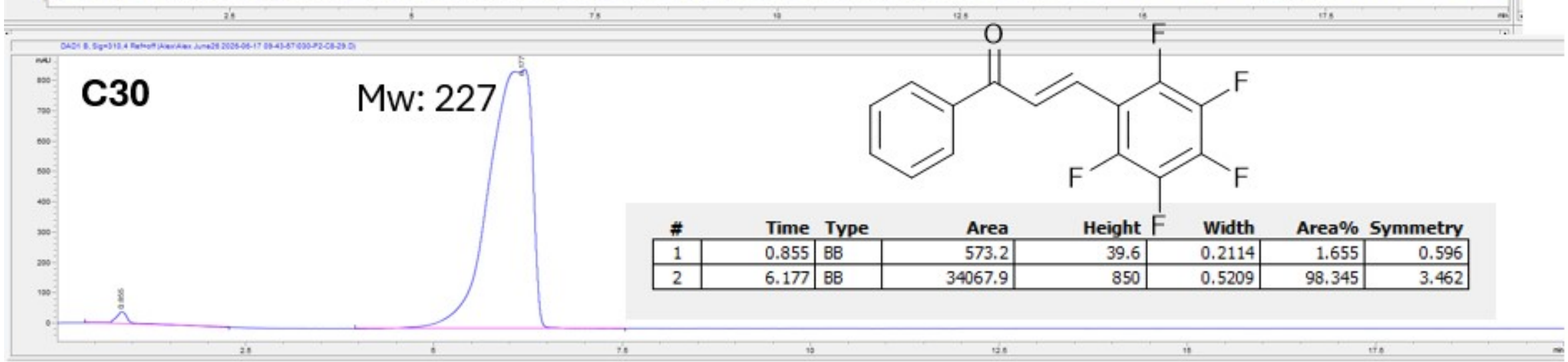

#	Time	Type	Area	Height	Width	Area%	Symmetry
1	0.855	BB	573.2	39.6	0.2114	1.655	0.596
2	6.177	BB	34067.9	850	0.5209	98.345	3.462

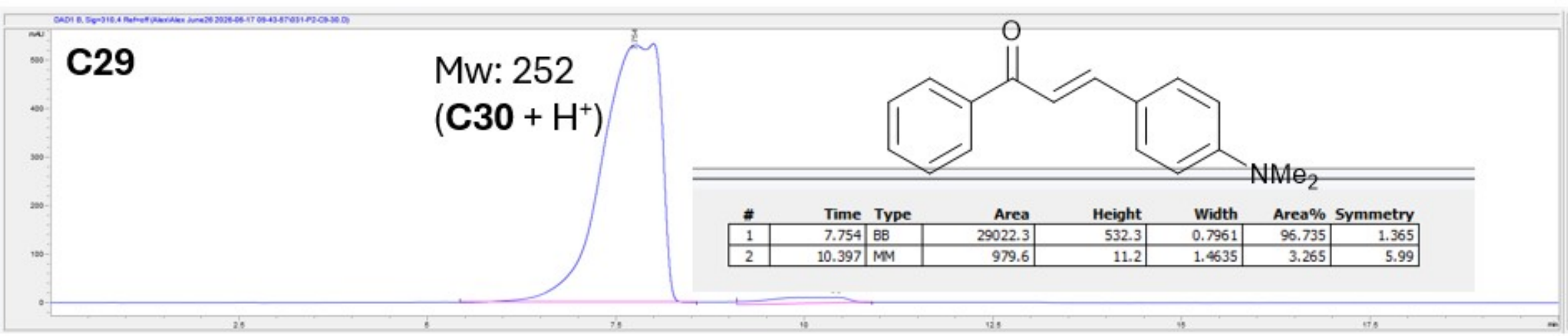

#	Time	Type	Area	Height	Width	Area%	Symmetry
1	7.754	BB	29022.3	532.3	0.7961	96.735	1.365
2	10.397	MM	979.6	11.2	1.4635	3.265	5.99

50% MeCN 50% H_2O (0.1% formic acid), 1mL/min, 310nm, 25uL injection ,

Poroshell C18 4um 4.6 x 100mm column

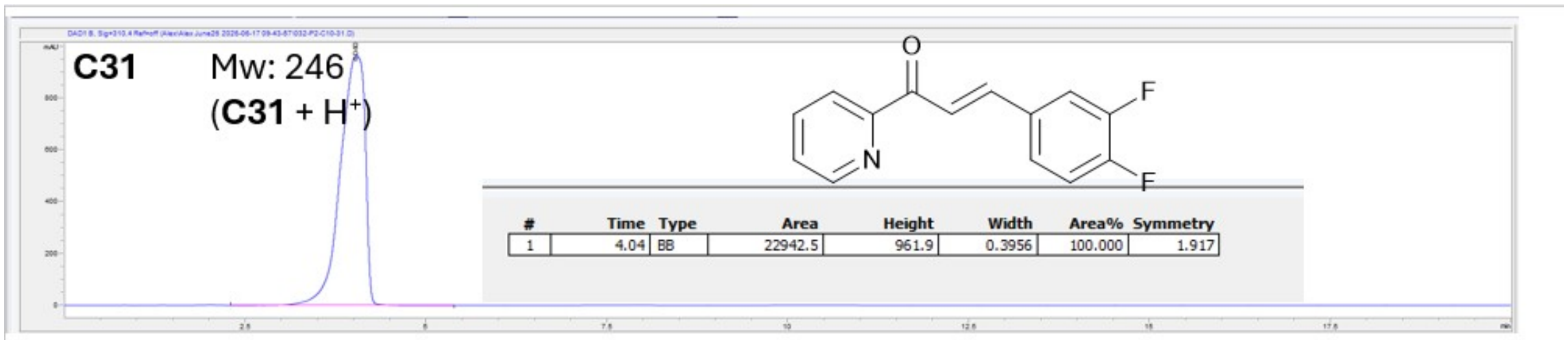

#	Time	Type	Area	Height	Width	Area%	Symmetry
1	4.04	BB	22942.5	961.9	0.3956	100.000	1.917

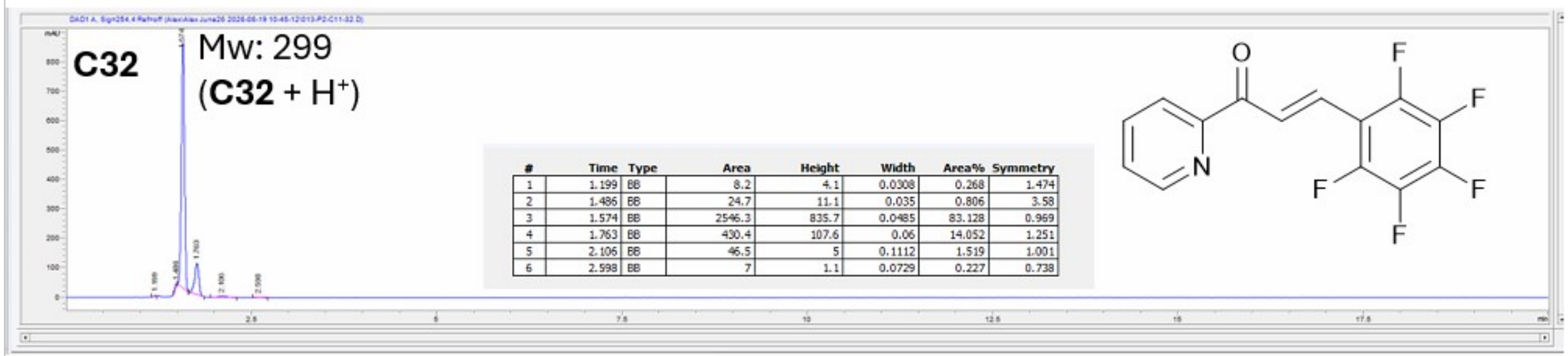

#	Time	Type	Area	Height	Width	Area%	Symmetry
1	1.199	BB	8.2	4.1	0.0308	0.268	1.474
2	1.486	BB	24.7	11.1	0.035	0.806	3.58
3	1.574	BB	2546.3	835.7	0.0485	83.128	0.969
4	1.763	BB	430.4	107.6	0.06	14.052	1.251
5	2.106	BB	46.5	5	0.1112	1.519	1.001
6	2.598	BB	7	1.1	0.0729	0.227	0.738

50% MeCN 50% H_2O (0.1% formic acid), 1mL/min, 310nm, 25uL injection ,

Poroshell C18 4um 4.6 x 100mm column

Initial Fluorescence Study (ESI S4)

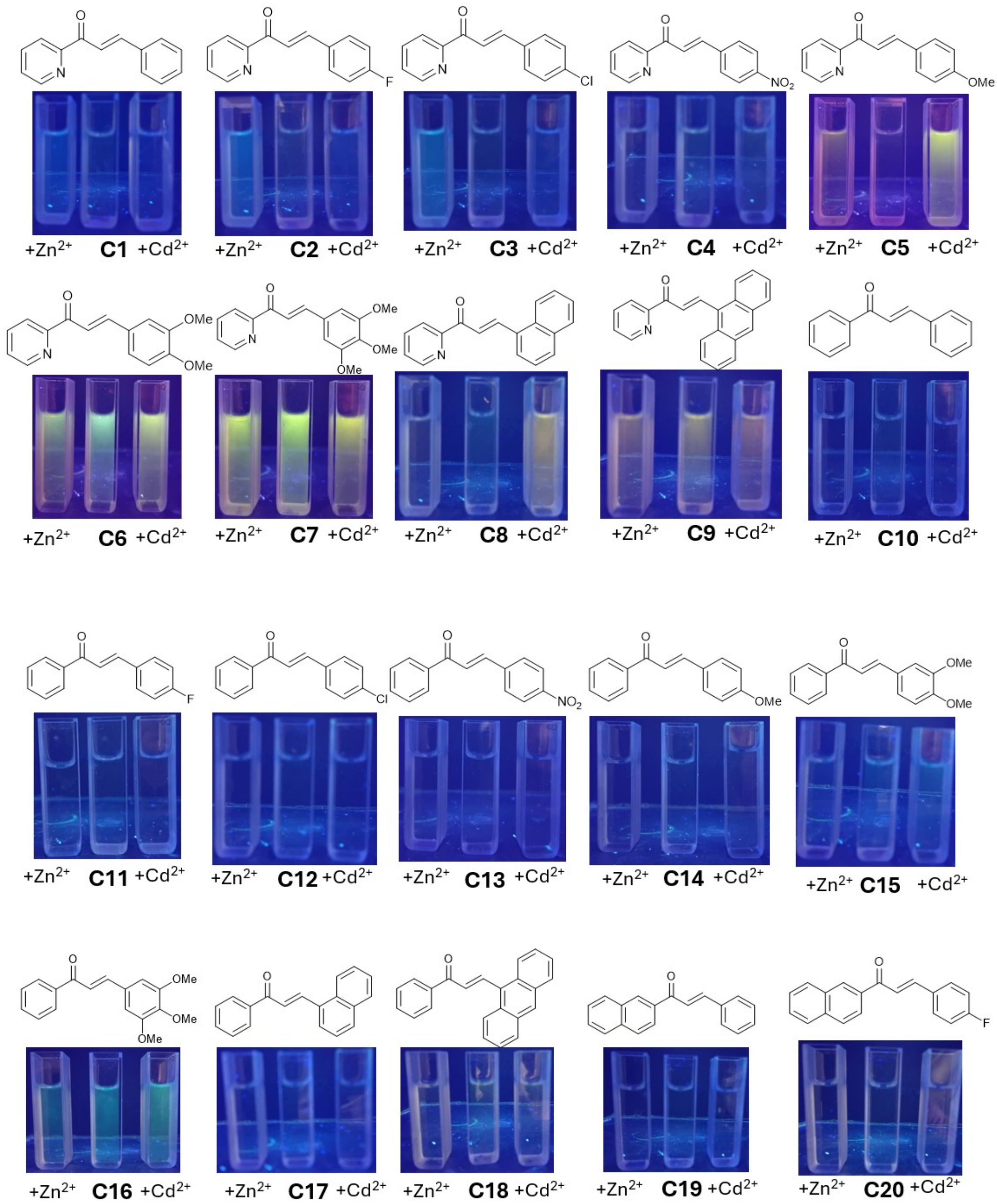

+Zn2+ C1 +Cd2+
+Zn2+ C2 +Cd2+
+Zn2+ C3 +Cd2+
+Zn2+ C4 +Cd2+
+Zn2+ C5 +Cd2+
+Zn2+ C6 +Cd2+
+Zn2+ C7 +Cd2+
+Zn2+ C8 +Cd2+
+Zn2+ C9 +Cd2+
+Zn2+ C10 +Cd2+
+Zn2+ C11 +Cd2+
+Zn2+ C12 +Cd2+
+Zn2+ C13 +Cd2+
+Zn2+ C14 +Cd2+
+Zn2+ C15 +Cd2+
+Zn2+ C16 +Cd2+
+Zn2+ C17 +Cd2+
+Zn2+ C18 +Cd2+
+Zn2+ C19 +Cd2+
+Zn2+ C20 +Cd2+

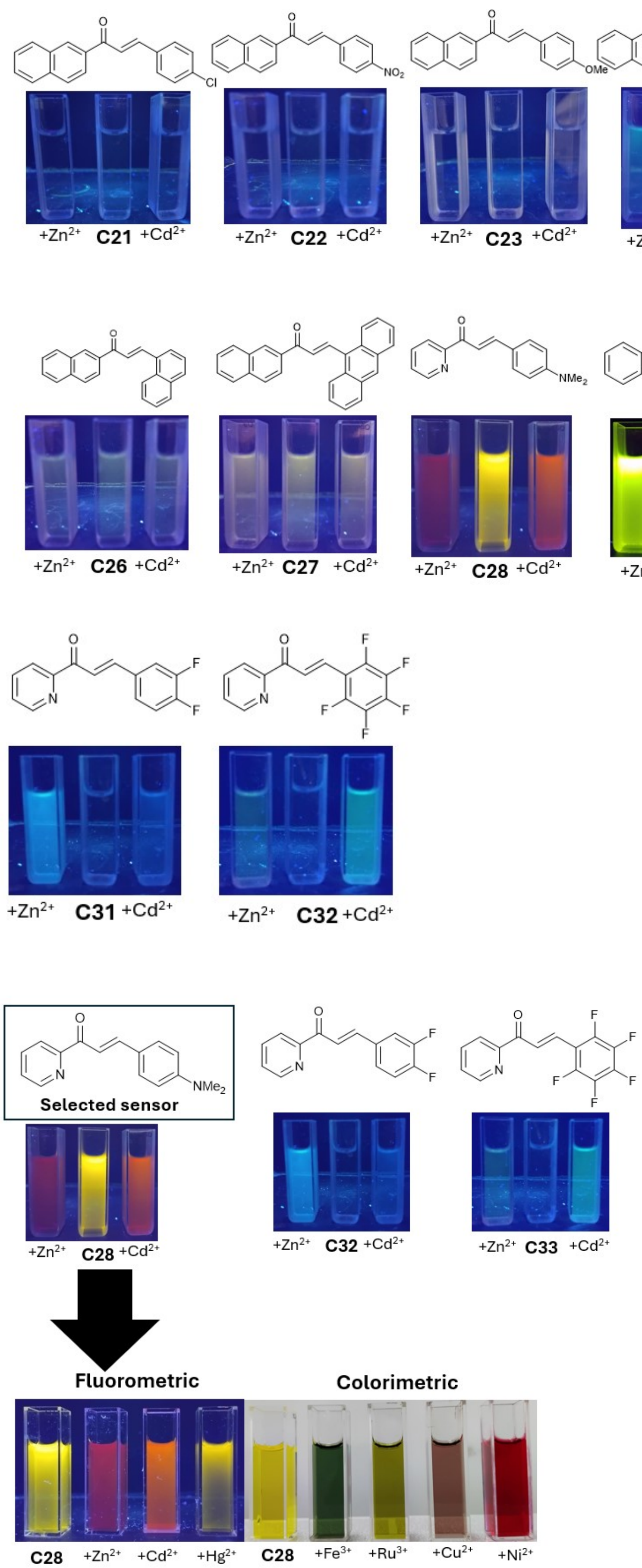

+Zn2+ C21 +Cd2+
+Zn2+ C22 +Cd2+
+Zn2+ C23 +Cd2+
+Zn2+ C24 +Cd2+
+Zn2+ C25 +Cd2+
+Zn2+ C26 +Cd2+
+Zn2+ C27 +Cd2+
+Zn2+ C28 +Cd2+
+Zn2+ C29 +Cd2+
+Zn2+ C30 +Cd2+
+Zn2+ C31 +Cd2+
+Zn2+ C32 +Cd2+
Selected sensor
+Zn2+ C28 +Cd2+
+Zn2+ C32 +Cd2+
+Zn2+ C33 +Cd2+
Fluorometric
Colorimetric
C28 +Zn2+ +Cd2+ +Hg2+
C28 +Fe3+ +Ru3+ +Cu2+ +Ni2+

High throughput Fluorescence Screening (ESI S5)

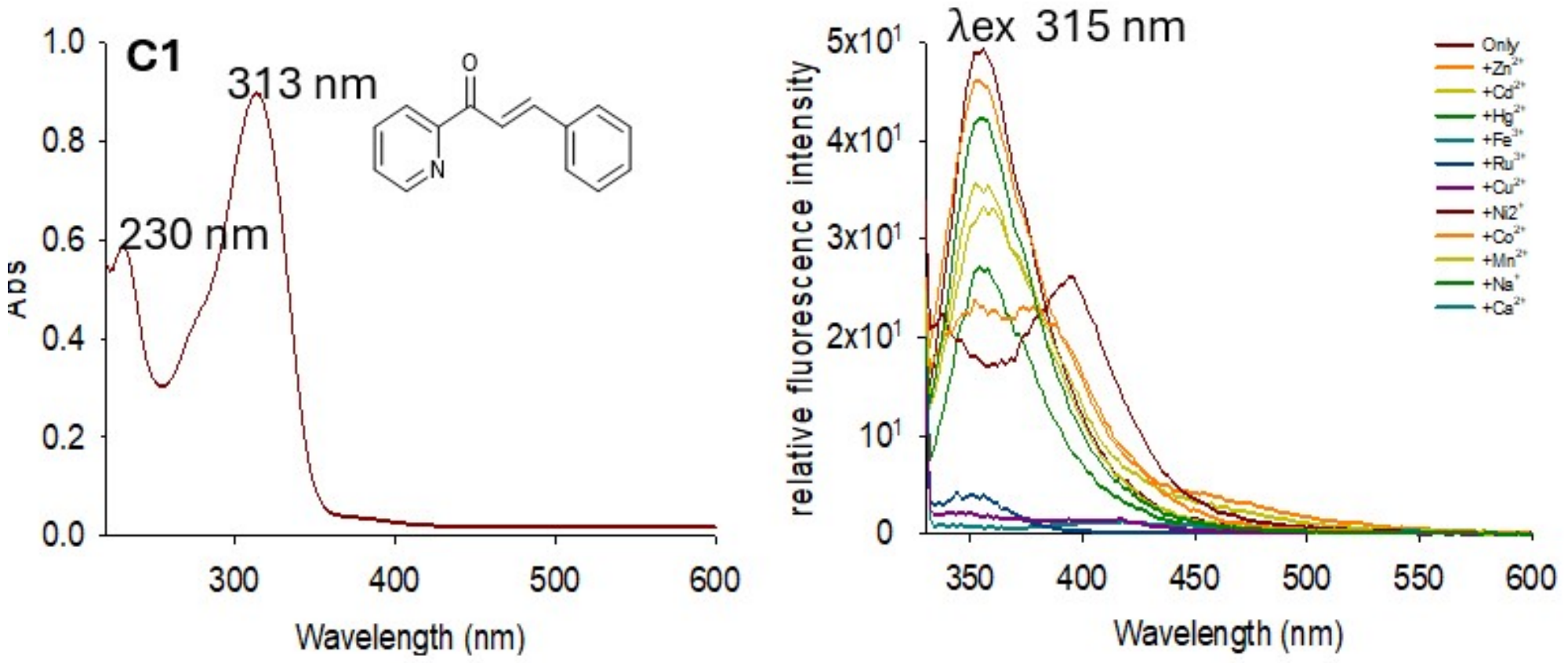

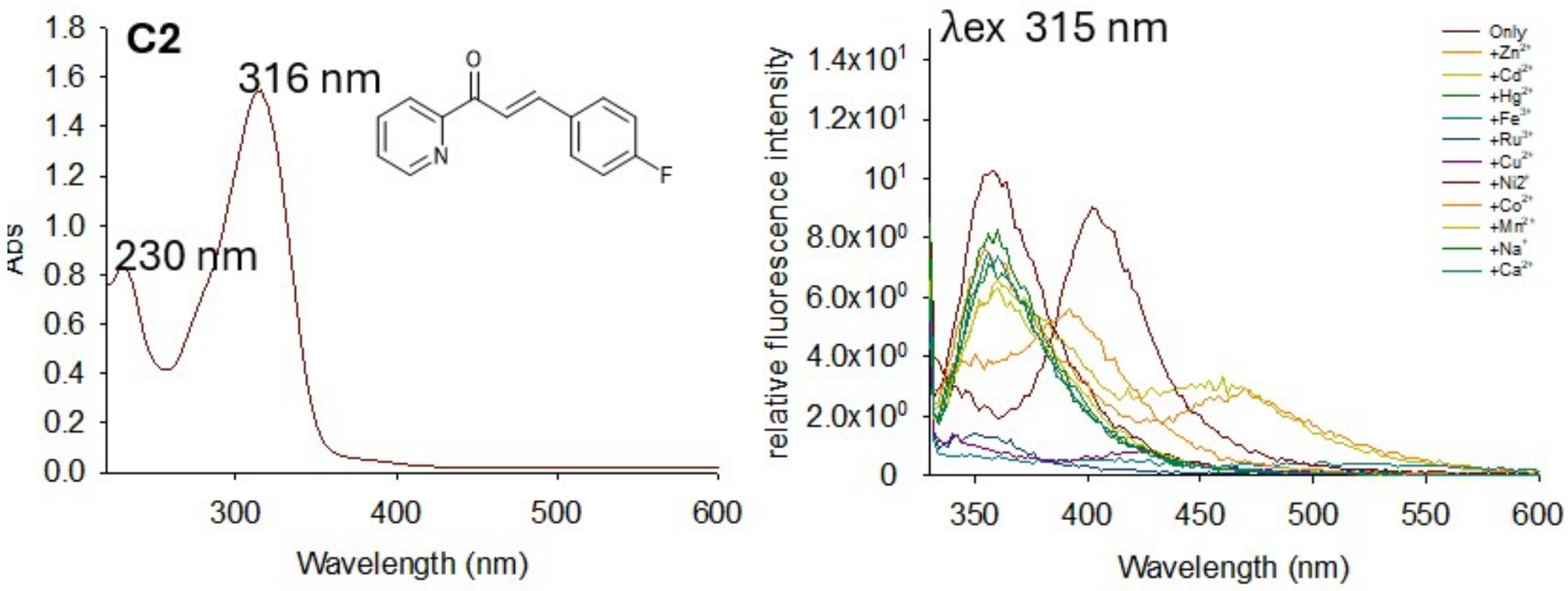

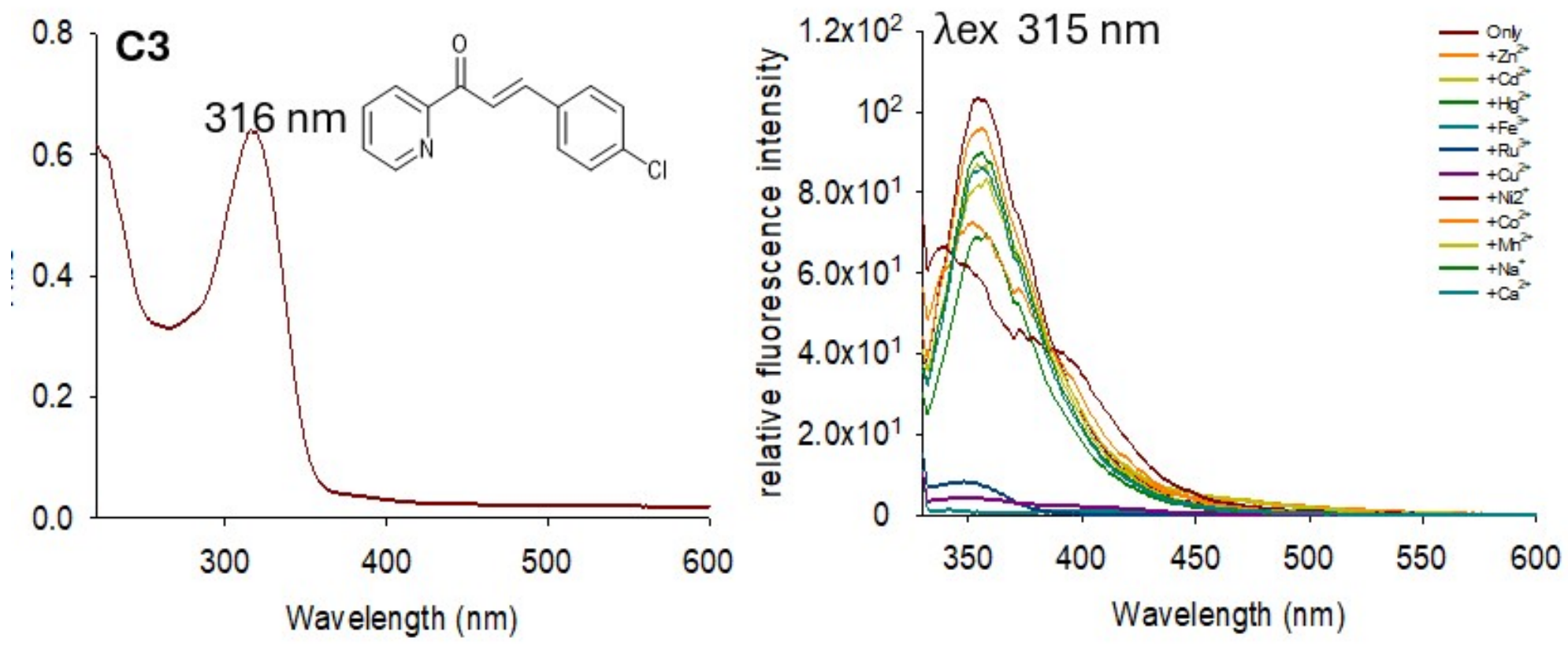

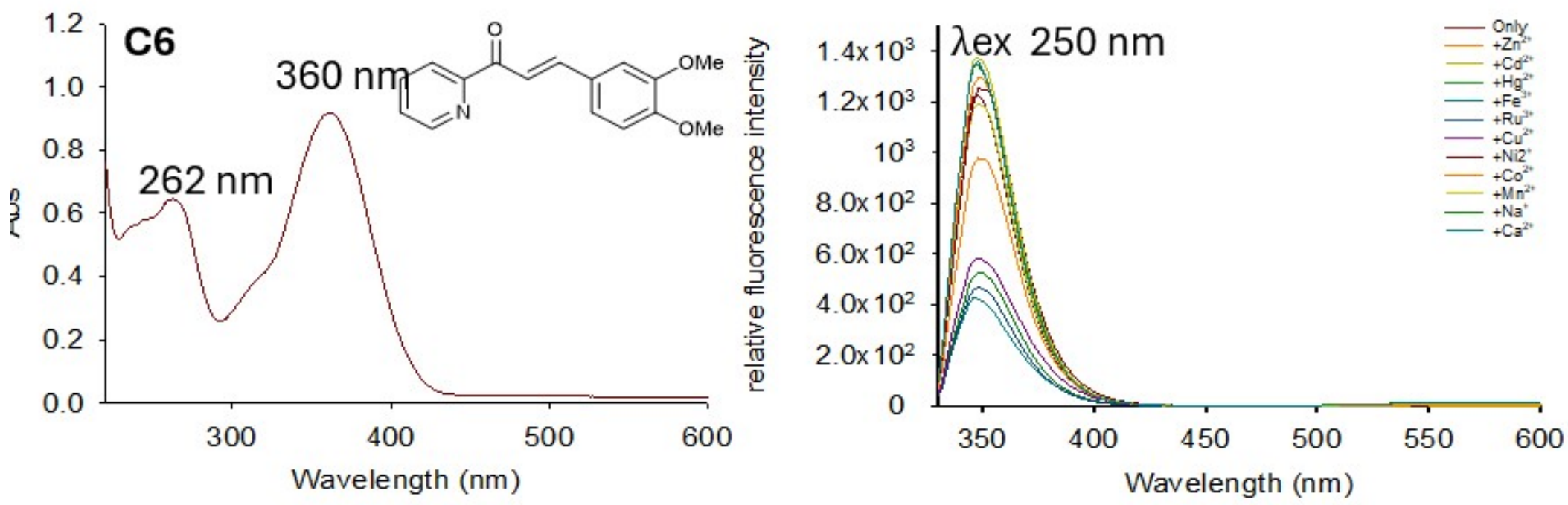

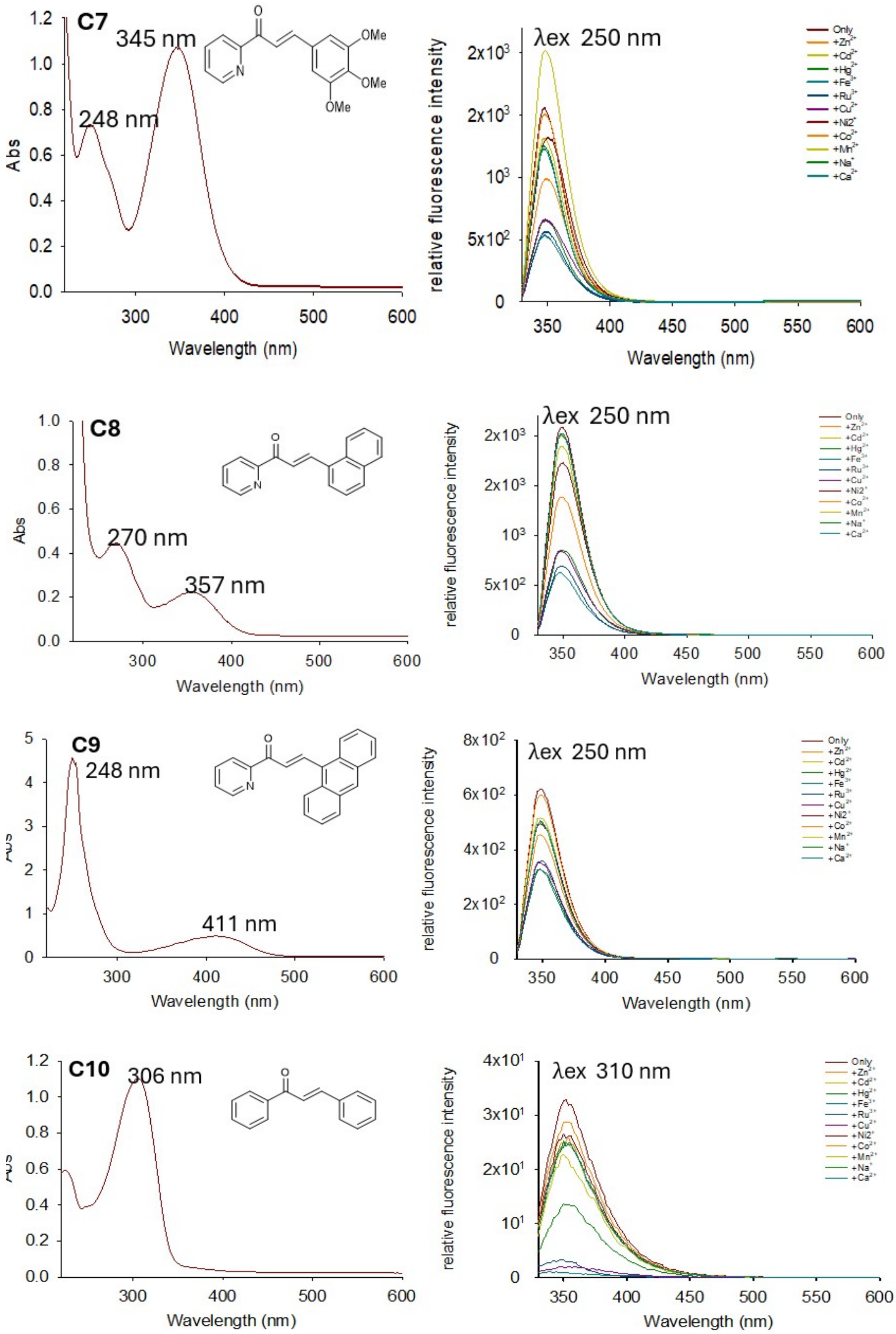

C7
345 nm
248 nm
OMe
OMe
OMe
N
O
Abs
Wavelength (nm)
λex 250 nm
relative fluorescence intensity
Only
+Zn2+
+Cd2+
+Hg2+
+Fe3+
+Ru3+
+Cu2+
+Ni2+
+Co2+
+Mn2+
+Na+
+Ca2+
C8
270 nm
357 nm
λex 250 nm
C9
248 nm
411 nm
λex 250 nm
C10
306 nm
λex 310 nm

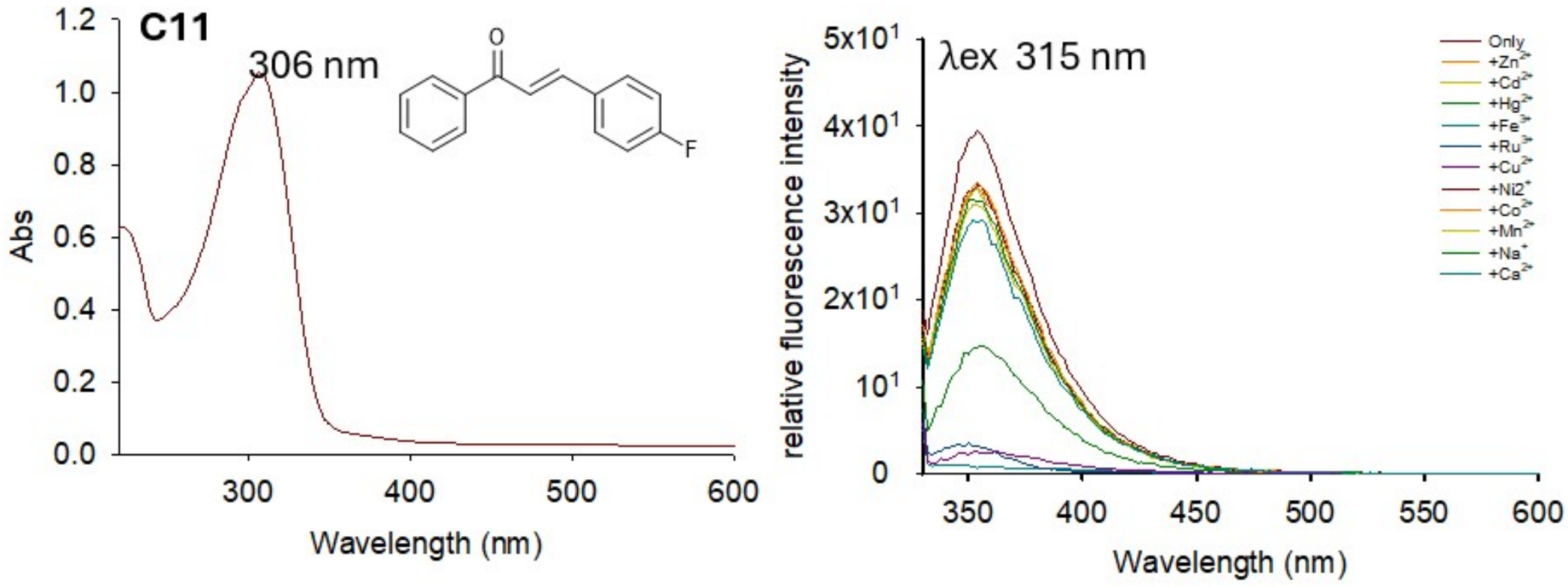
C11
306 nm
Abs
Wavelength (nm)
λex 315 nm
relative fluorescence intensity
Wavelength (nm)
Only
+Zn2+
+Cd2+
+Hg2+
+Fe3+
+Ru3+
+Cu2+
+Ni2+
+Co2+
+Mn2+
+Na+
+Ca2+

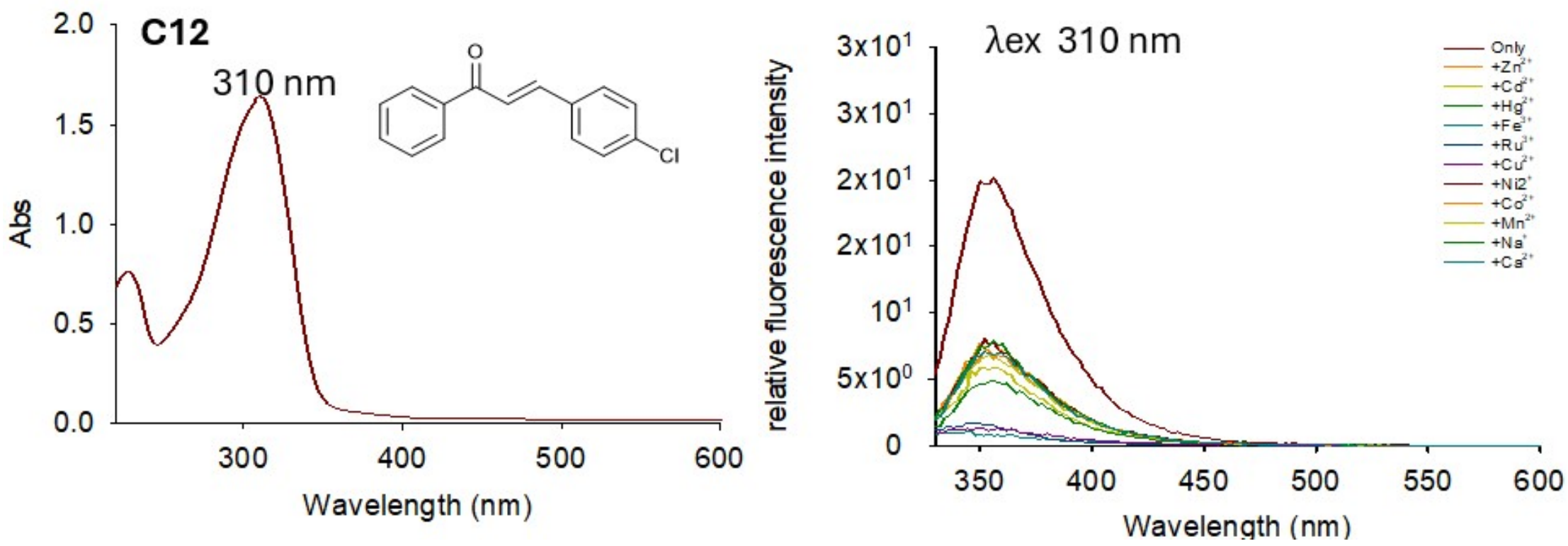
C12
310 nm
Abs
Wavelength (nm)
λex 310 nm
relative fluorescence intensity
Wavelength (nm)
Only
+Zn2+
+Cd2+
+Hg2+
+Fe3+
+Ru3+
+Cu2+
+Ni2+
+Co2+
+Mn2+
+Na+
+Ca2+

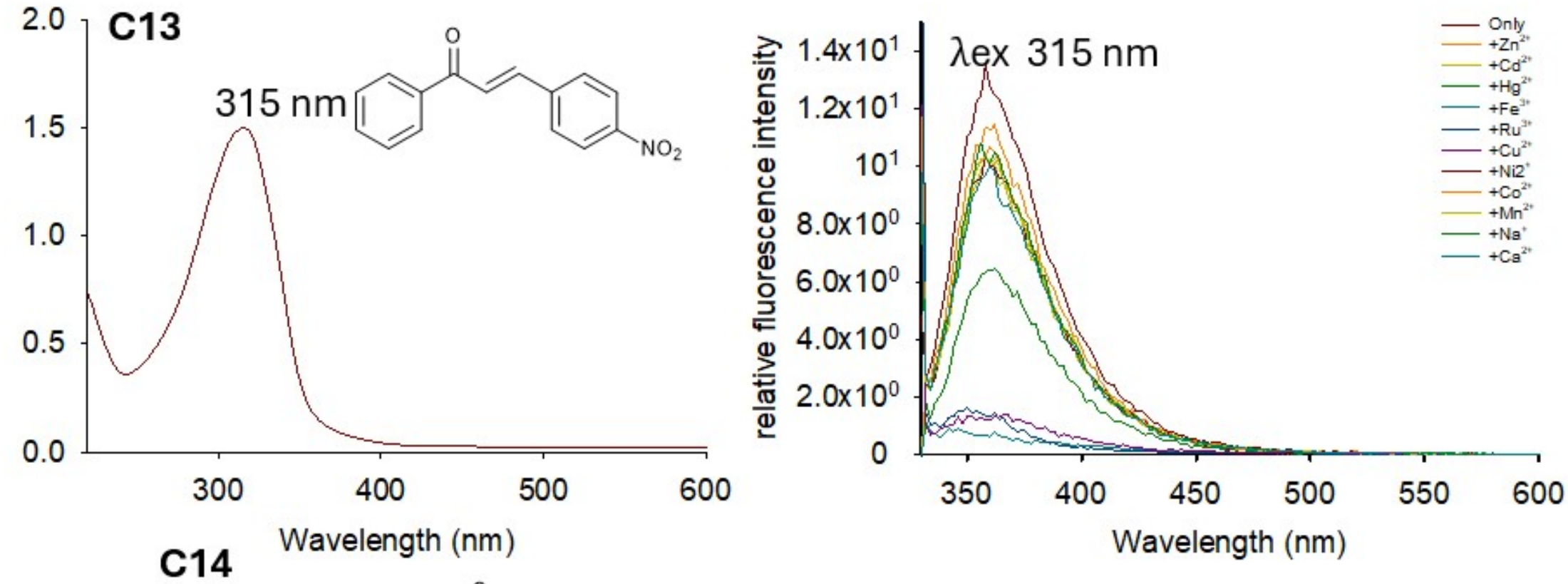
C13
315 nm
Wavelength (nm)
λex 315 nm
relative fluorescence intensity
Wavelength (nm)
Only
+Zn2+
+Cd2+
+Hg2+
+Fe3+
+Ru3+
+Cu2+
+Ni2+
+Co2+
+Mn2+
+Na+
+Ca2+

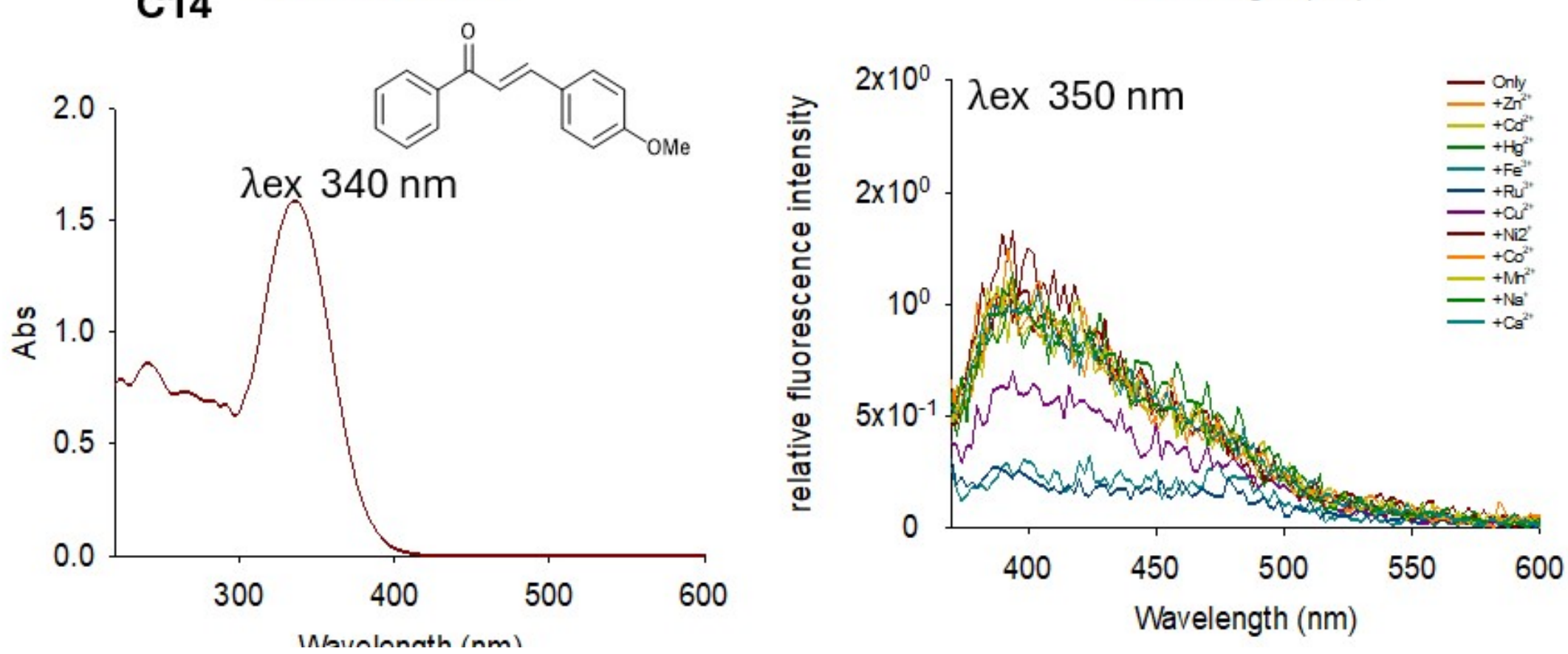
C14
λex 340 nm
Abs
λex 350 nm
relative fluorescence intensity
Wavelength (nm)
Only
+Zn2+
+Cd2+
+Hg2+
+Fe3+
+Ru3+
+Cu2+
+Ni2+
+Co2+
+Mn2+
+Na+
+Ca2+

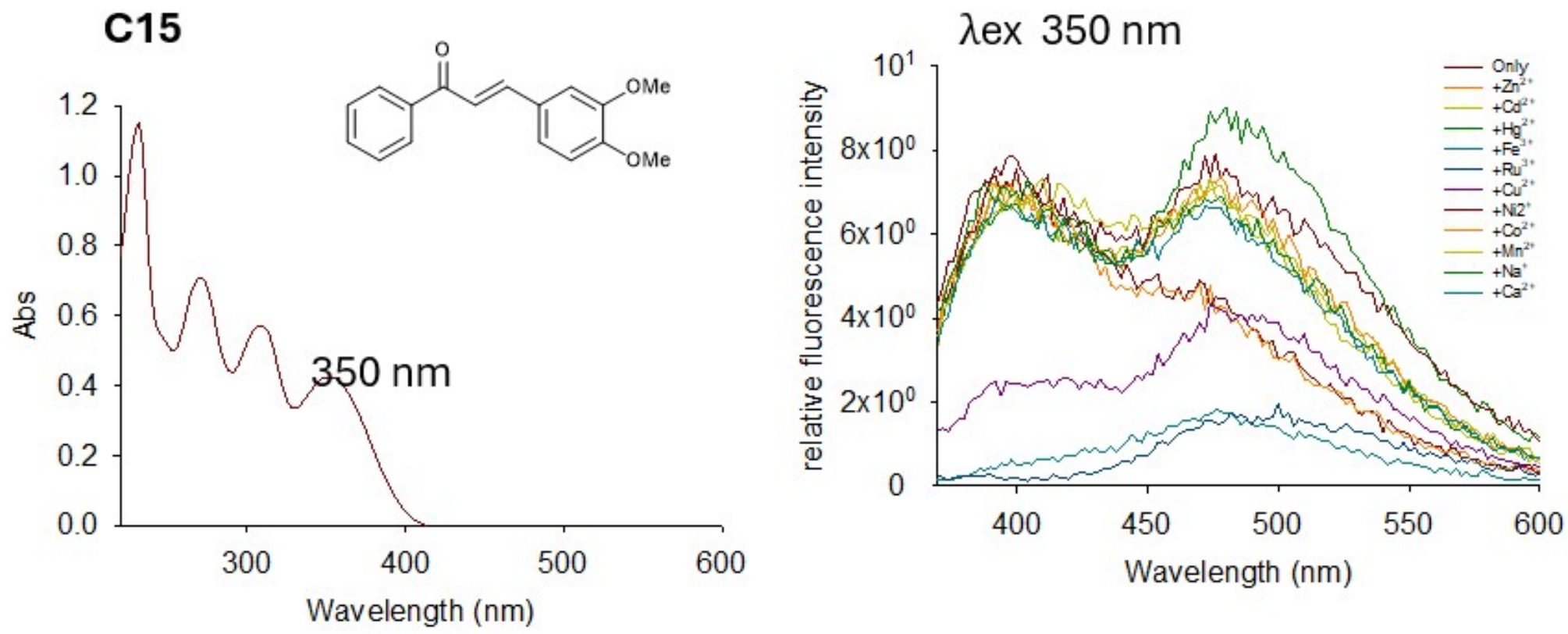
C15
OMe
OMe
350 nm
Abs
Wavelength (nm)
λex 350 nm
relative fluorescence intensity
Wavelength (nm)
Only
+Zn2+
+Cd2+
+Hg2+
+Fe3+
+Ru3+
+Cu2+
+Ni2+
+Co2+
+Mn2+
+Na+
+Ca2+

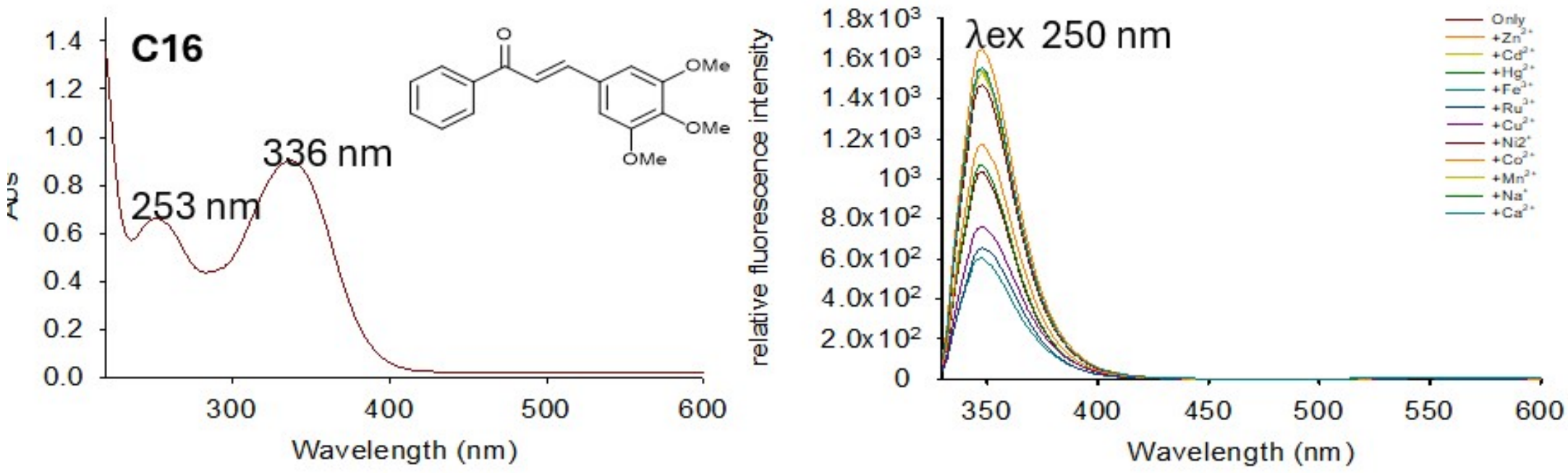
C16
OMe
OMe
OMe
253 nm
336 nm
Wavelength (nm)
λex 250 nm
relative fluorescence intensity
Wavelength (nm)
Only
+Zn2+
+Cd2+
+Hg2+
+Fe3+
+Ru3+
+Cu2+
+Ni2+
+Co2+
+Mn2+
+Na+
+Ca2+

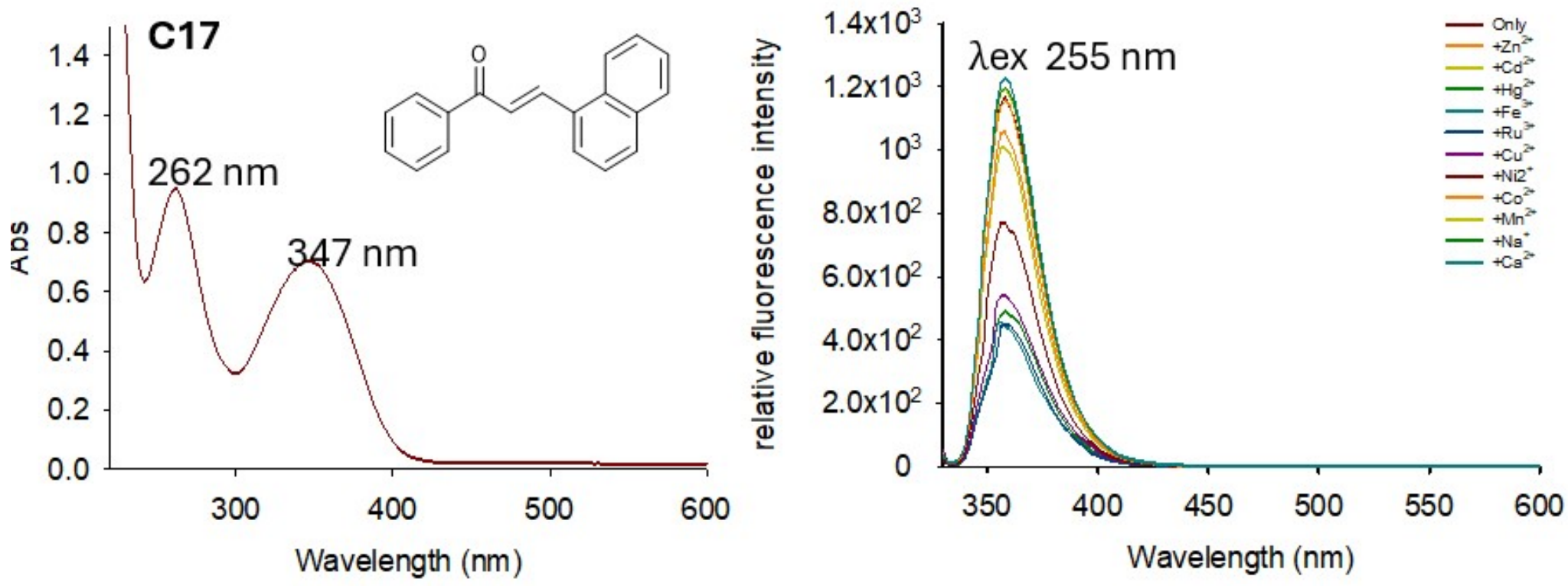
C17
262 nm
347 nm
Abs
Wavelength (nm)
λex 255 nm
relative fluorescence intensity
Wavelength (nm)
Only
+Zn2+
+Cd2+
+Hg2+
+Fe3+
+Ru3+
+Cu2+
+Ni2+
+Co2+
+Mn2+
+Na+
+Ca2+

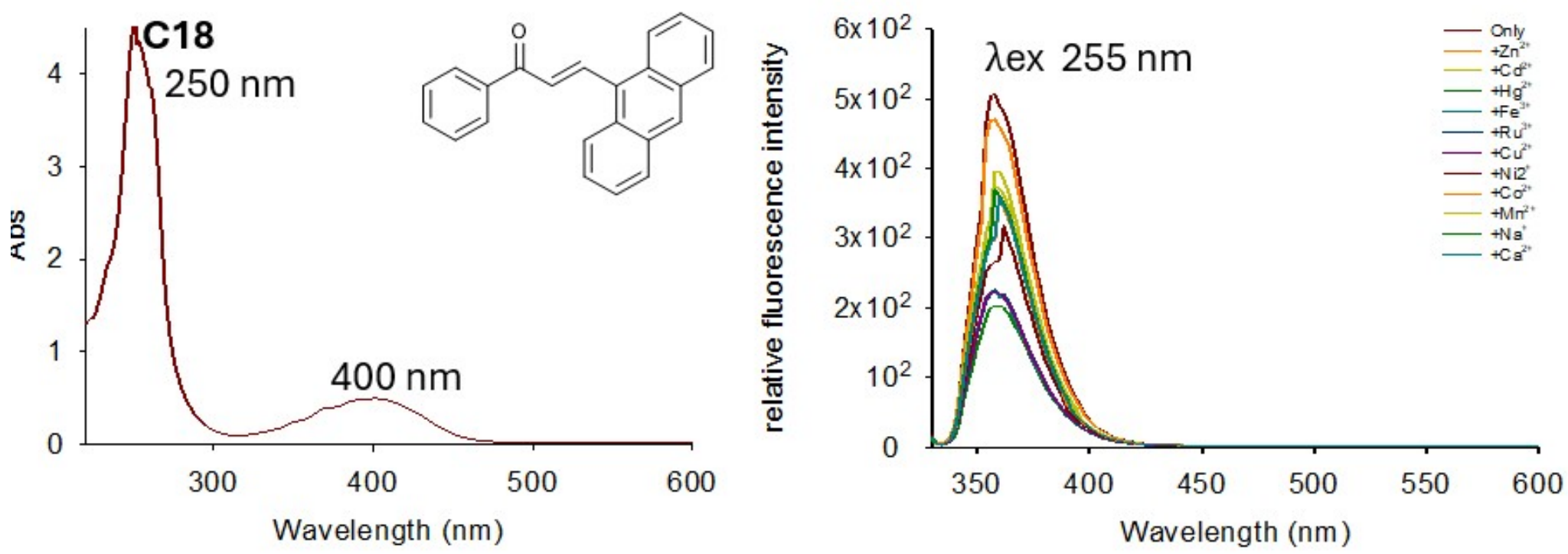
C18
250 nm
400 nm
Abs
Wavelength (nm)
λex 255 nm
relative fluorescence intensity
Wavelength (nm)
Only
+Zn2+
+Cd2+
+Hg2+
+Fe3+
+Ru3+
+Cu2+
+Ni2+
+Co2+
+Mn2+
+Na+
+Ca2+

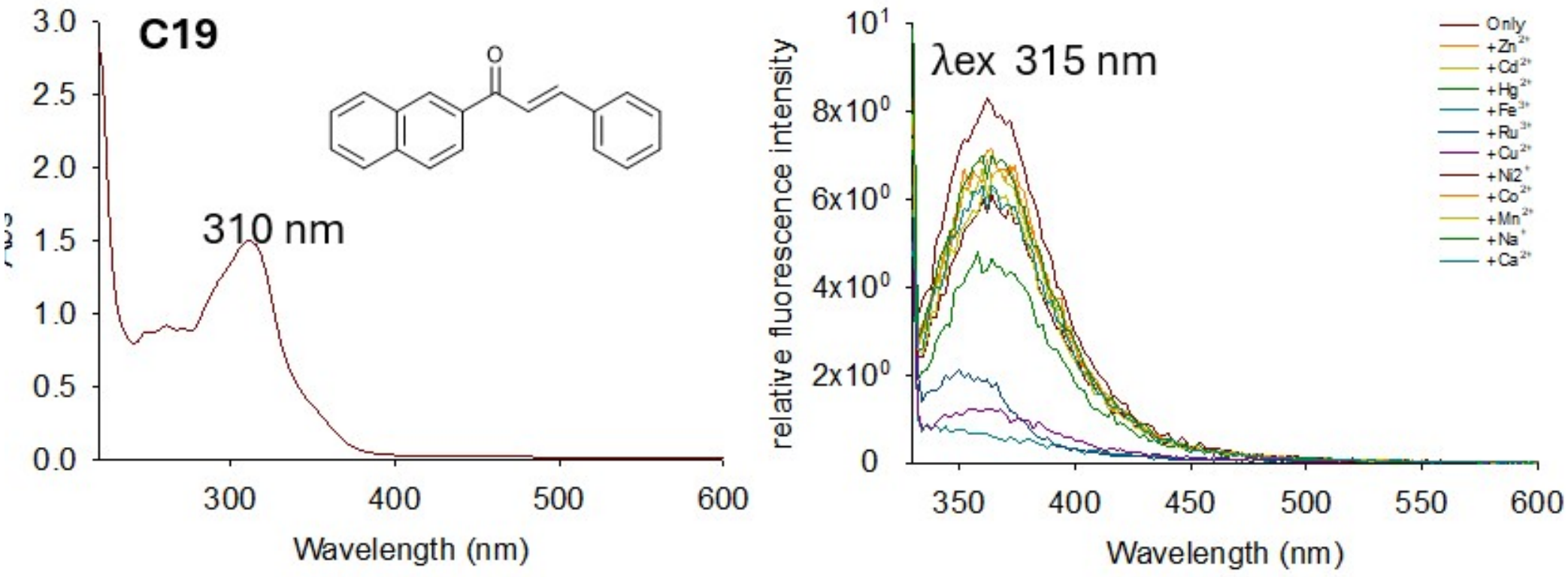
C19
310 nm
Wavelength (nm)
λex 315 nm
relative fluorescence intensity
Wavelength (nm)

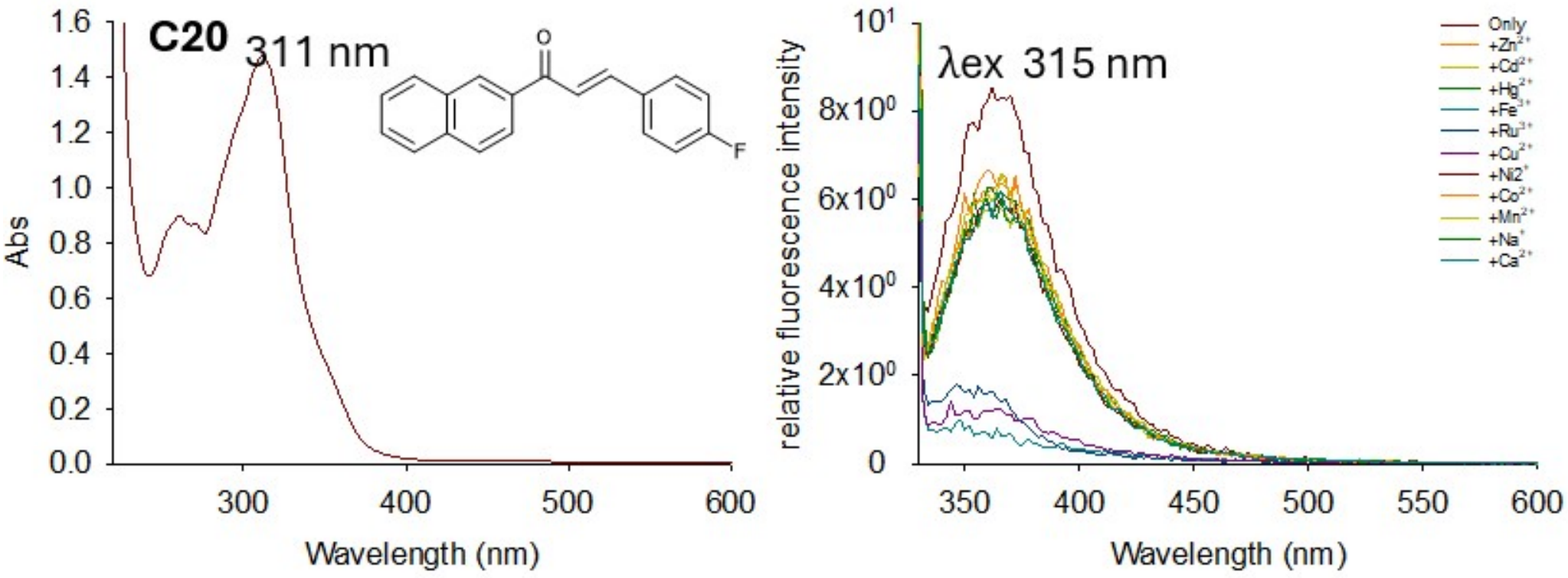
C20
311 nm
Abs
Wavelength (nm)
λex 315 nm
relative fluorescence intensity
Wavelength (nm)

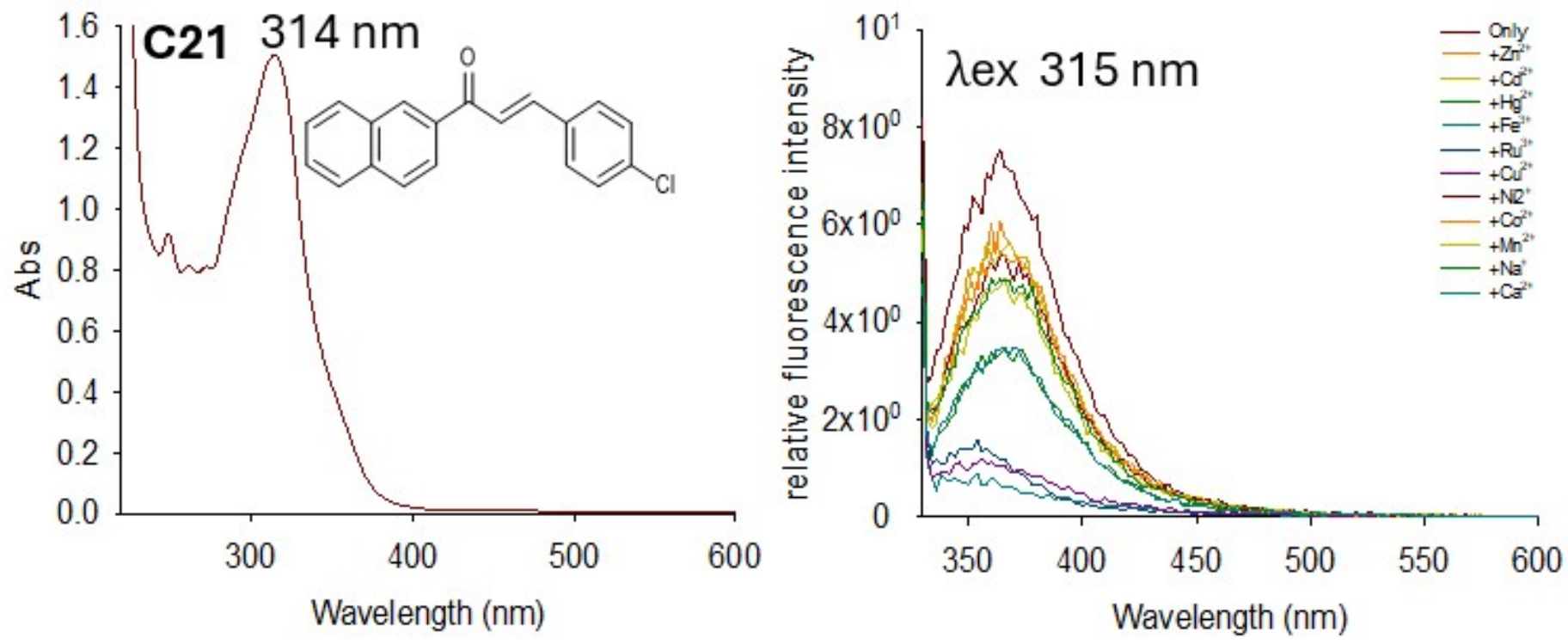
C21
314 nm
Abs
Wavelength (nm)
λex 315 nm
relative fluorescence intensity
Wavelength (nm)

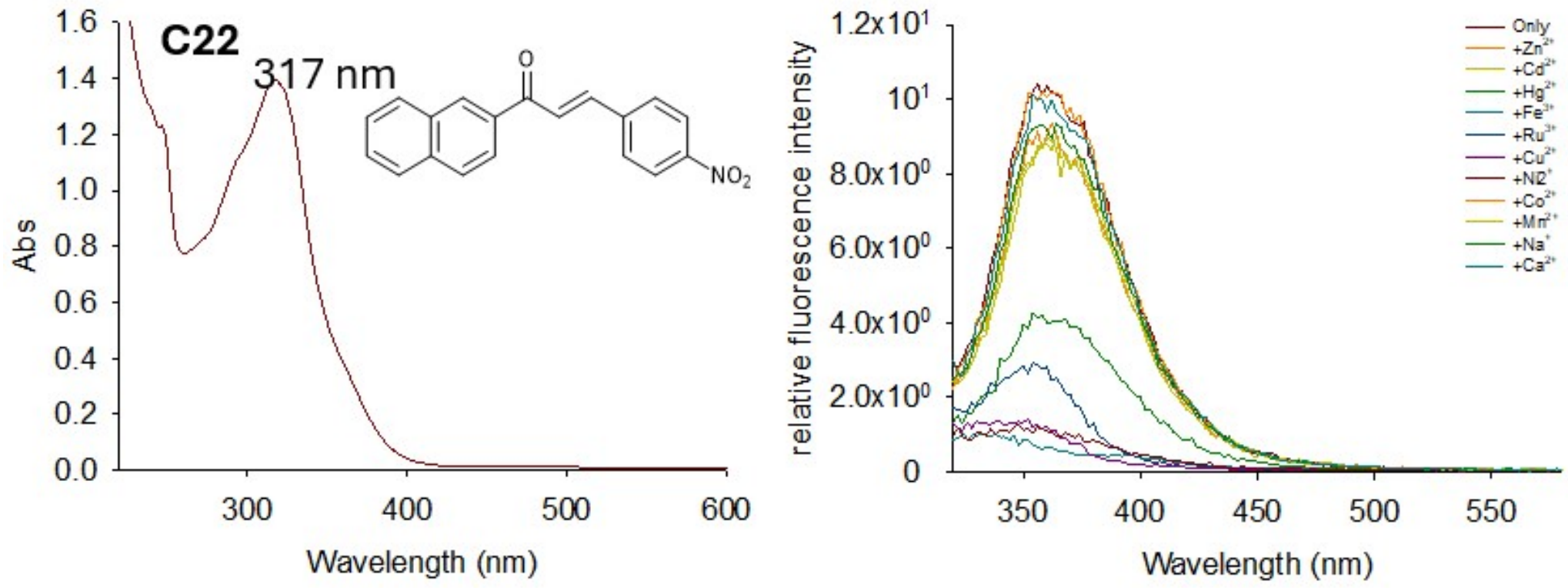
C22
317 nm
Abs
Wavelength (nm)
relative fluorescence intensity
Wavelength (nm)

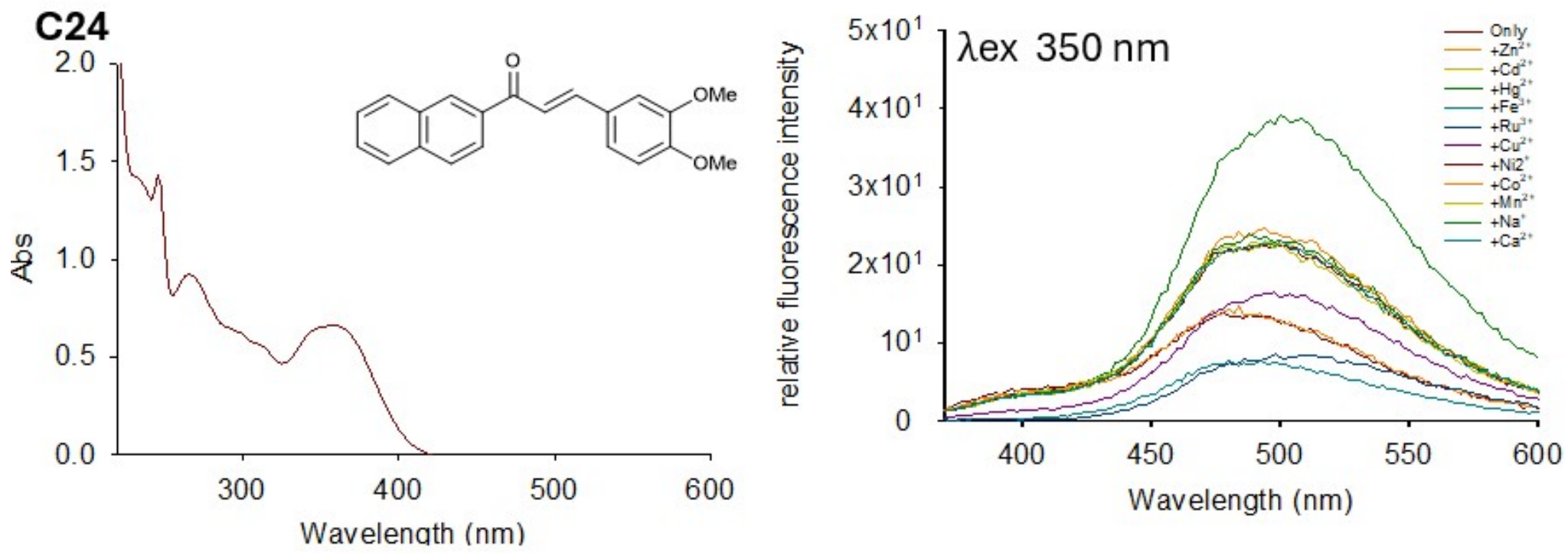
C24
Abs
Wavelength (nm)
OMe
OMe
λex 350 nm
relative fluorescence intensity
Wavelength (nm)
Only
+Zn2+
+Cd2+
+Hg2+
+Fe3+
+Ru3+
+Cu2+
+Ni2+
+Co2+
+Mn2+
+Na+
+Ca2+

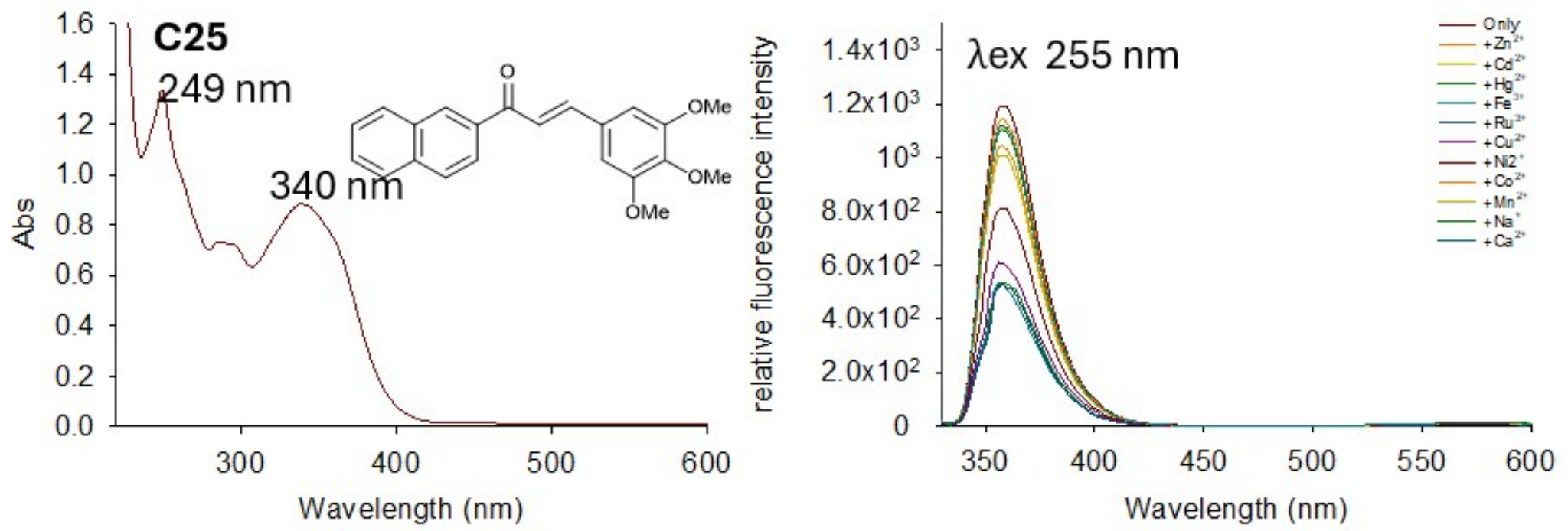
C25
249 nm
340 nm
Abs
Wavelength (nm)
OMe
OMe
OMe
λex 255 nm
relative fluorescence intensity
Wavelength (nm)

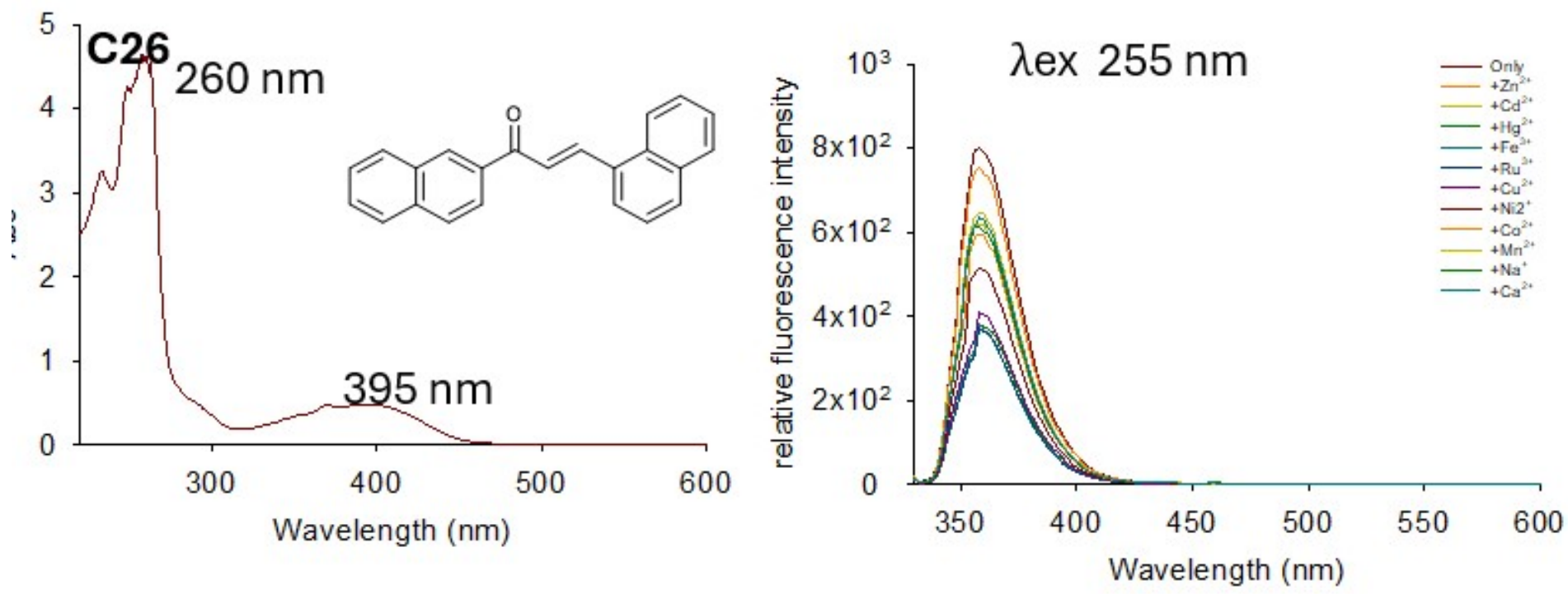
C26
260 nm
395 nm
Wavelength (nm)
λex 255 nm
relative fluorescence intensity
Wavelength (nm)

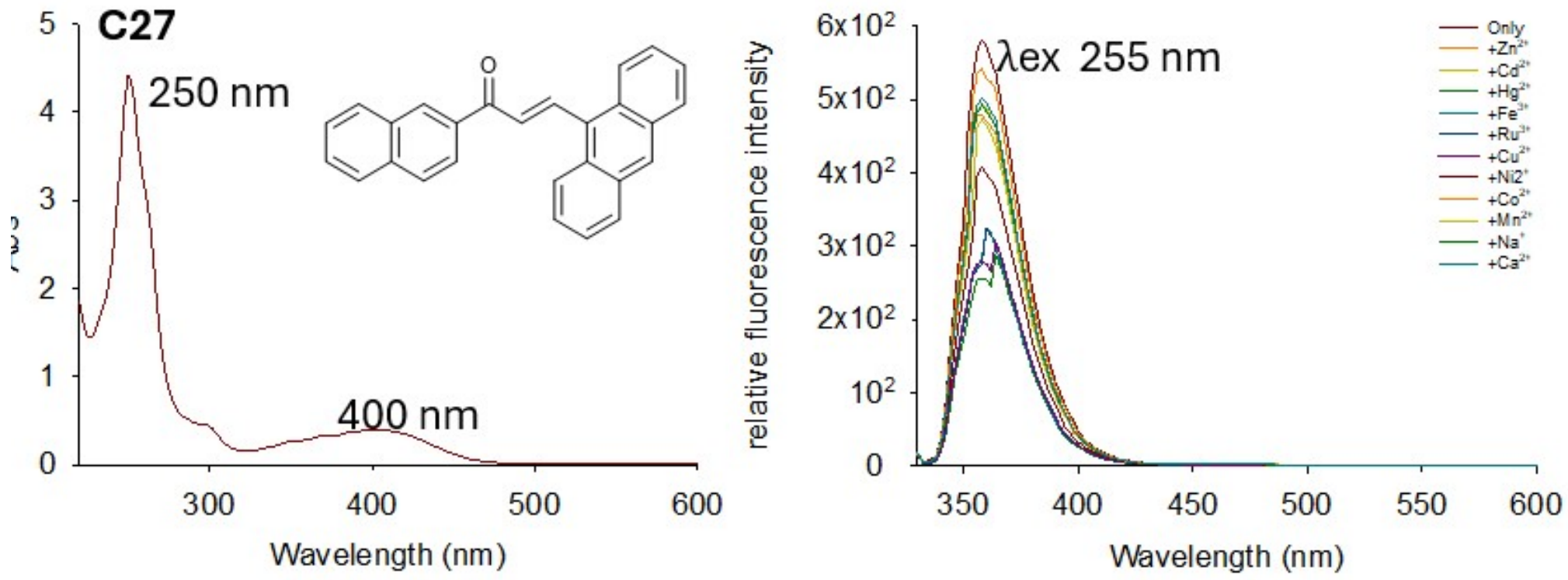
C27
250 nm
400 nm
Wavelength (nm)
λex 255 nm
relative fluorescence intensity
Wavelength (nm)

C28

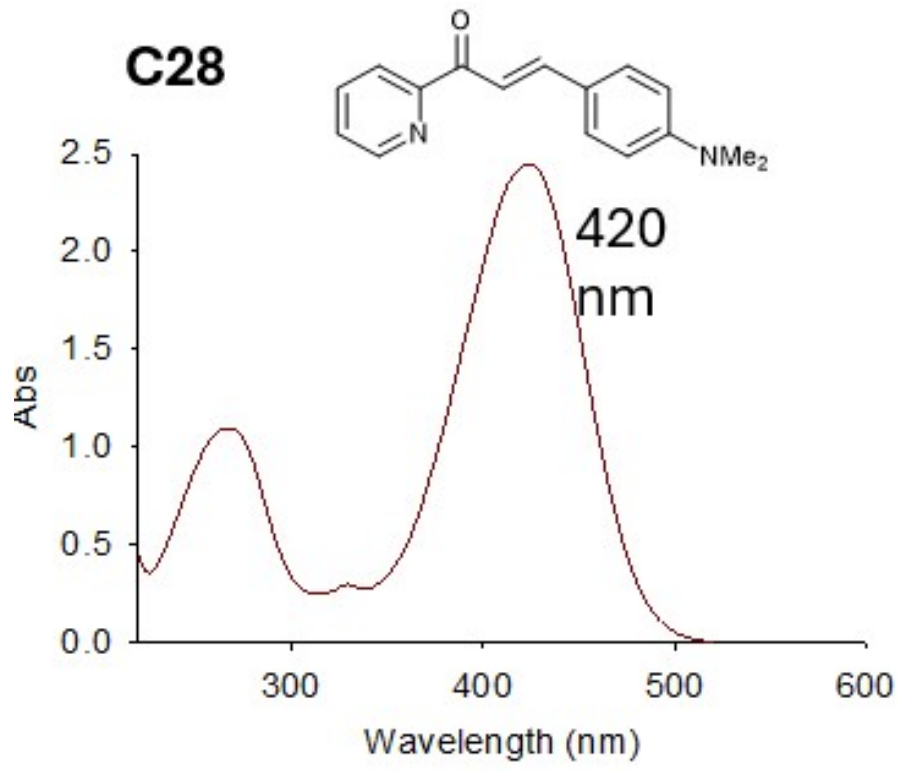

NMe$_2$
420 nm
Abs
Wavelength (nm)

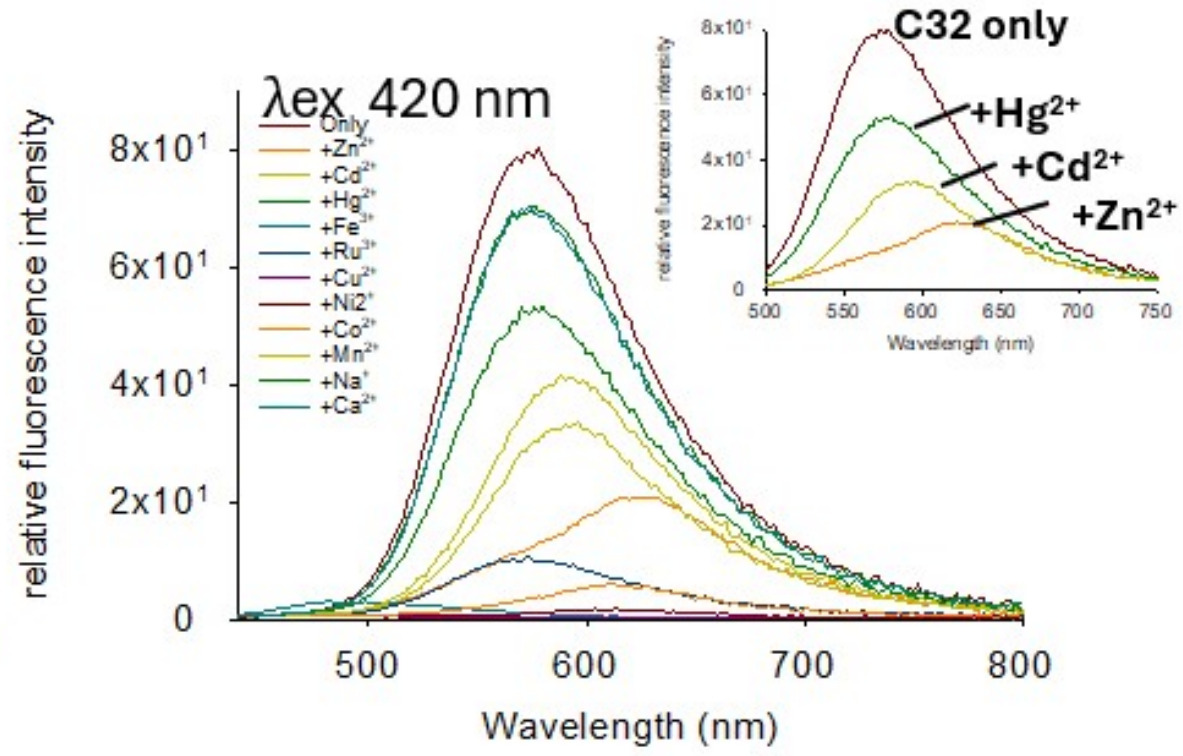

λex 420 nm
C32 only
+Hg^{2+}
+Cd^{2+}
+Zn^{2+}
relative fluorescence intensity
Wavelength (nm)

C29

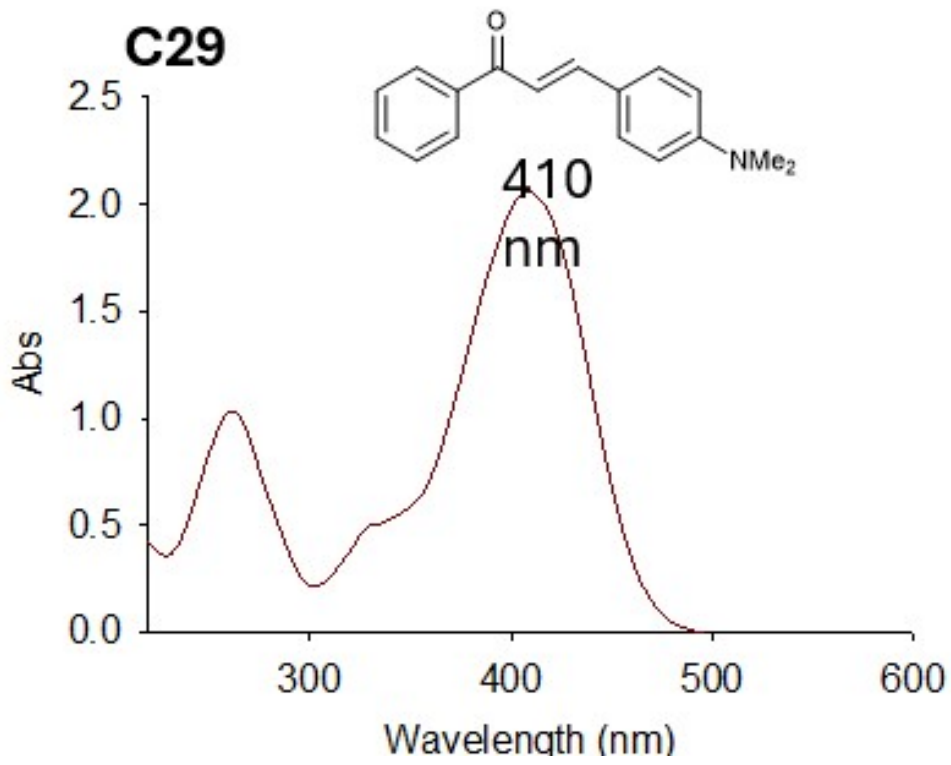

NMe$_2$
410 nm
Abs
Wavelength (nm)

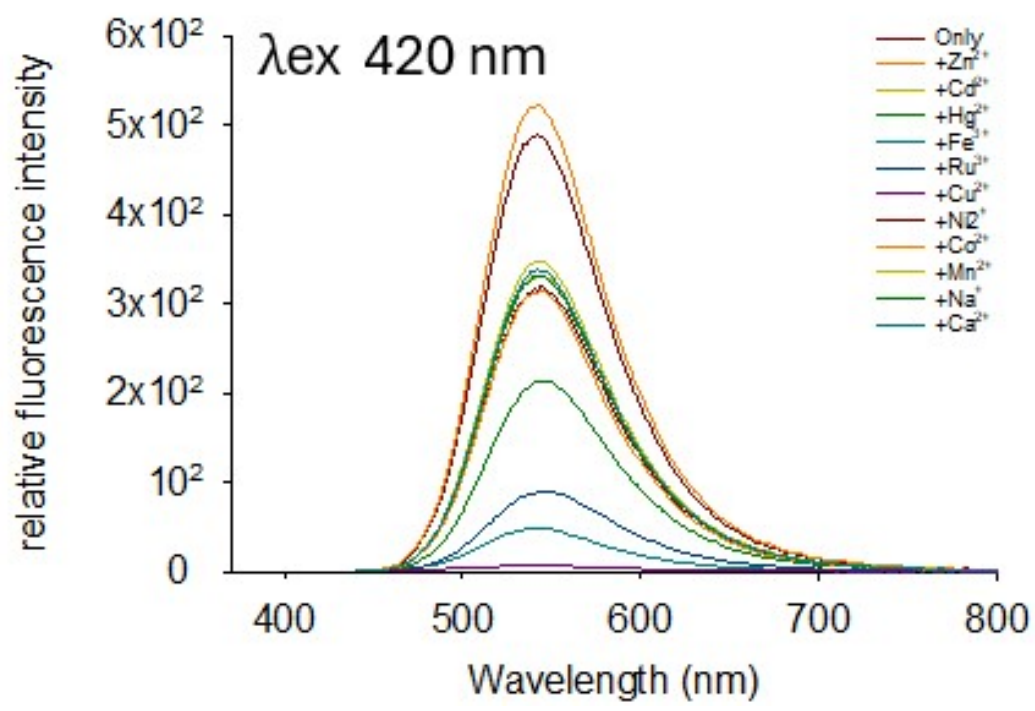

λex 420 nm
relative fluorescence intensity
Wavelength (nm)

C30

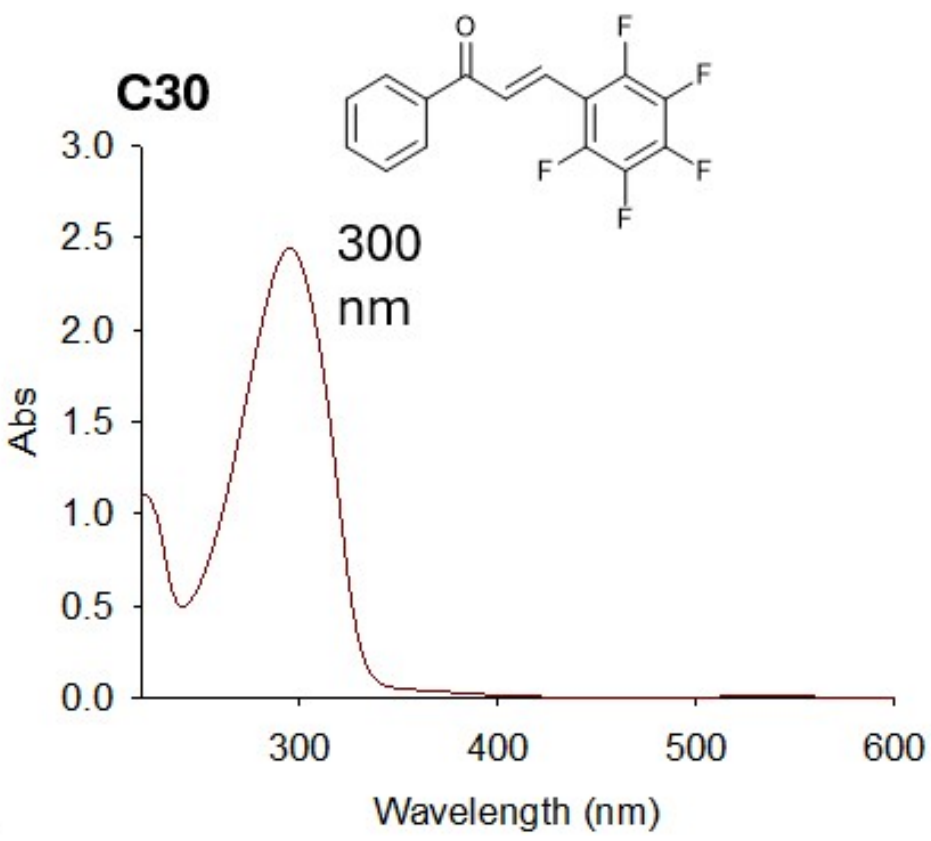

300 nm
Abs
Wavelength (nm)

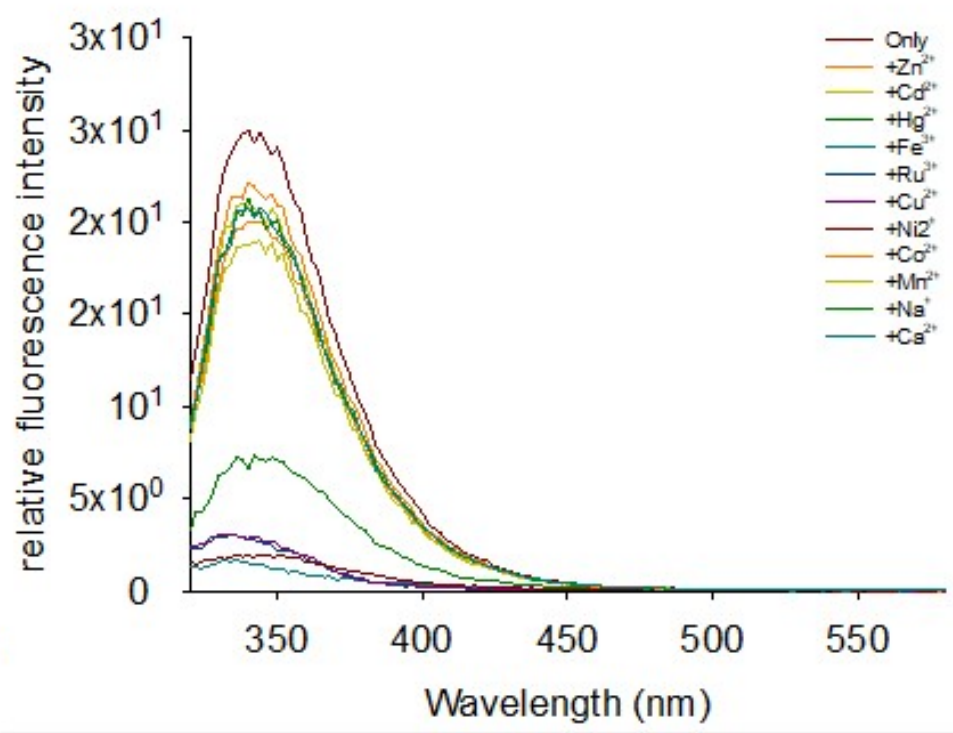

relative fluorescence intensity
Wavelength (nm)

C32

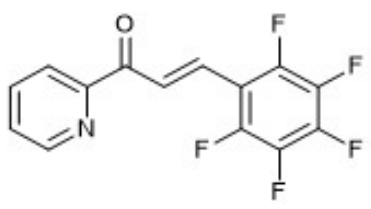

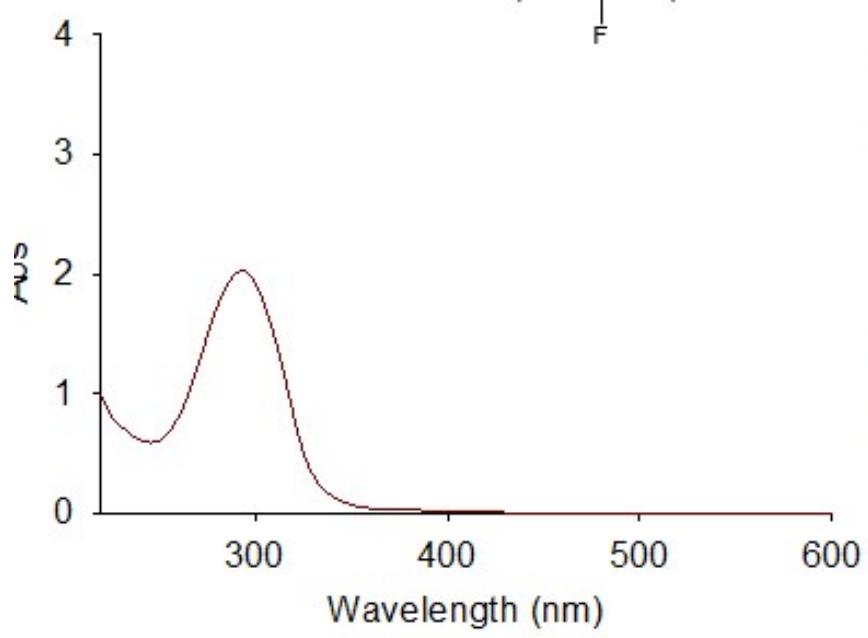

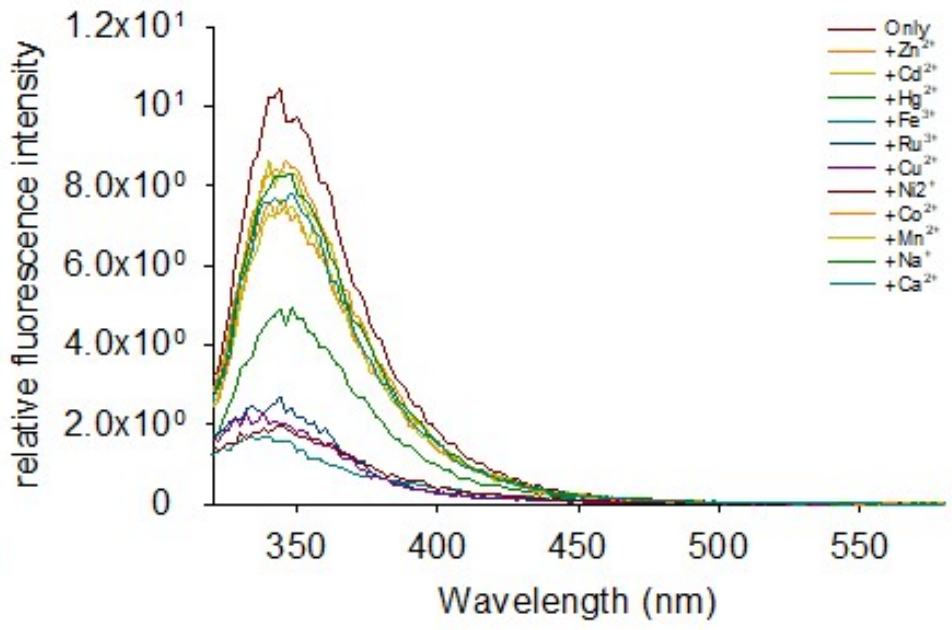

C31

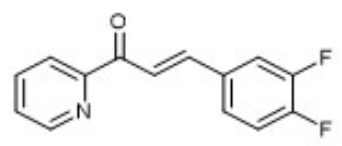

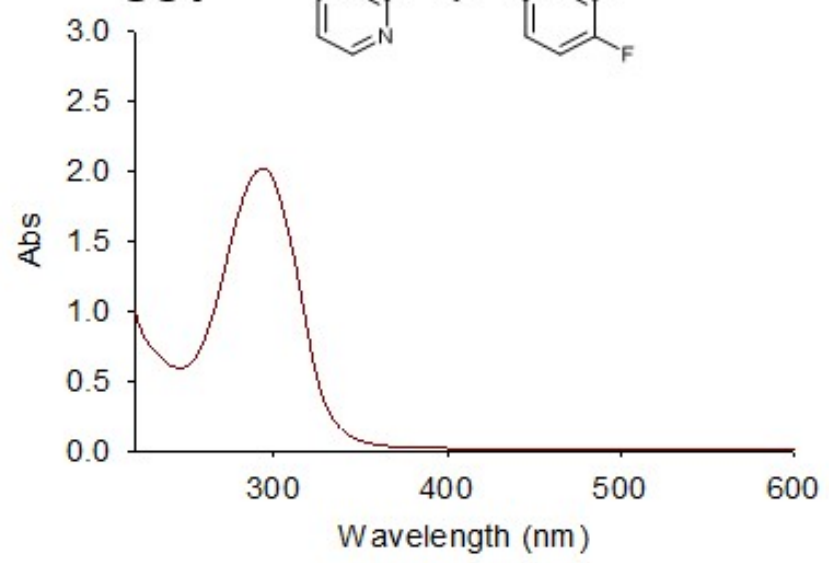

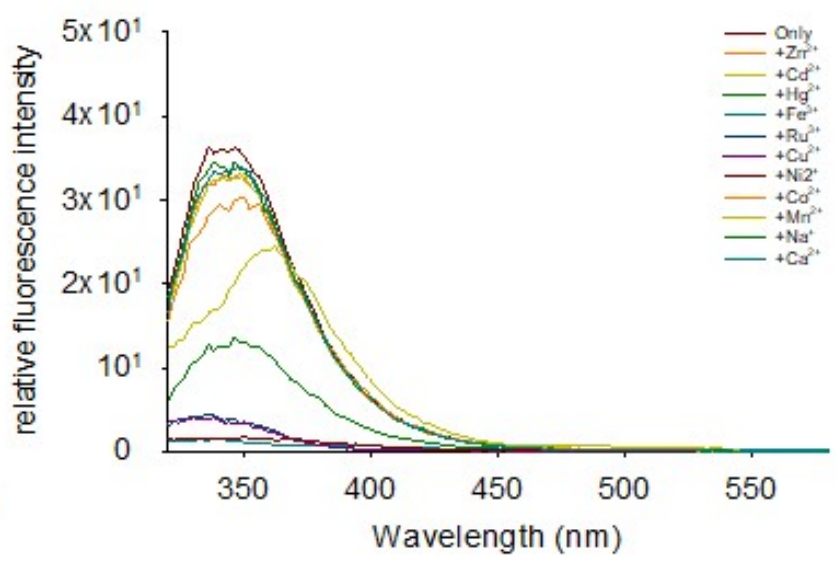

Limit of Detection Studies (ESI S6)

Fluorescence spectroscopy, 100% MeCN, C28 concentration: 50μM, λ_{ex} 420 nm

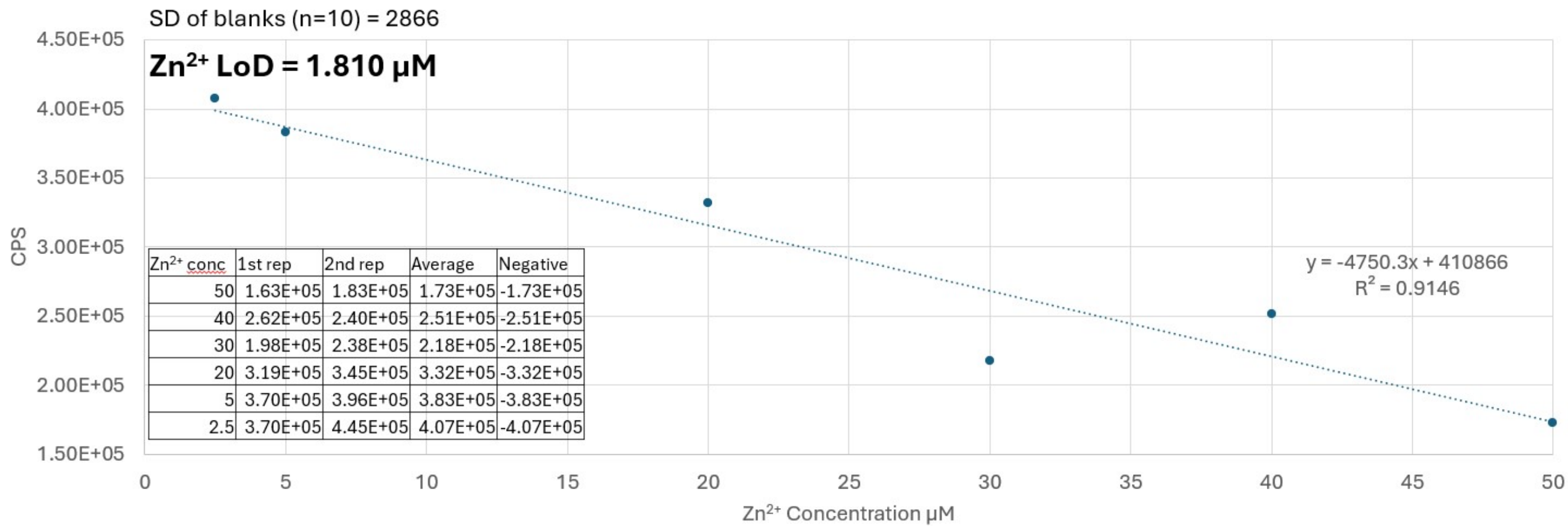

Zn^{2+} conc	1st rep	2nd rep	Average	Negative
50	1.63E+05	1.83E+05	1.73E+05	-1.73E+05
40	2.62E+05	2.40E+05	2.51E+05	-2.51E+05
30	1.98E+05	2.38E+05	2.18E+05	-2.18E+05
20	3.19E+05	3.45E+05	3.32E+05	-3.32E+05
5	3.70E+05	3.96E+05	3.83E+05	-3.83E+05
2.5	3.70E+05	4.45E+05	4.07E+05	-4.07E+05

Fluorescence spectroscopy, 100% MeCN, C28 concentration: 50μM, λ_{ex} 420 nm

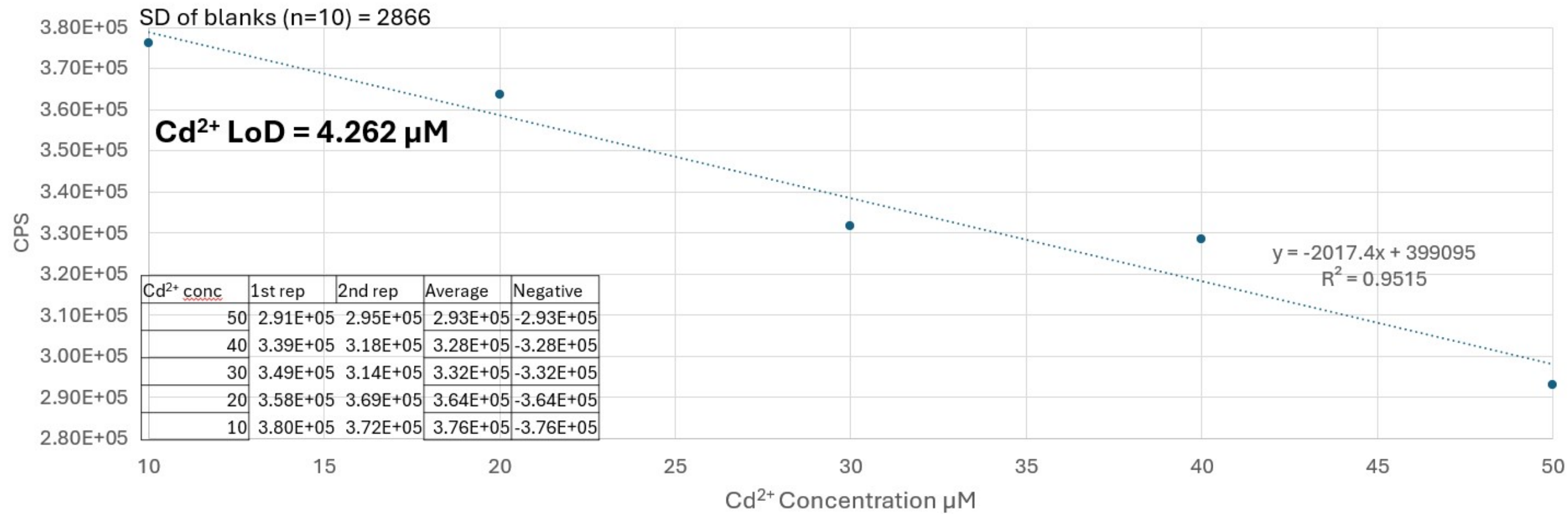

Cd^{2+} conc	1st rep	2nd rep	Average	Negative
50	2.91E+05	2.95E+05	2.93E+05	-2.93E+05
40	3.39E+05	3.18E+05	3.28E+05	-3.28E+05
30	3.49E+05	3.14E+05	3.32E+05	-3.32E+05
20	3.58E+05	3.69E+05	3.64E+05	-3.64E+05
10	3.80E+05	3.72E+05	3.76E+05	-3.76E+05

Fluorescence spectroscopy, 100% MeCN, C28 concentration: 50μM, λ_{ex} 420 nm

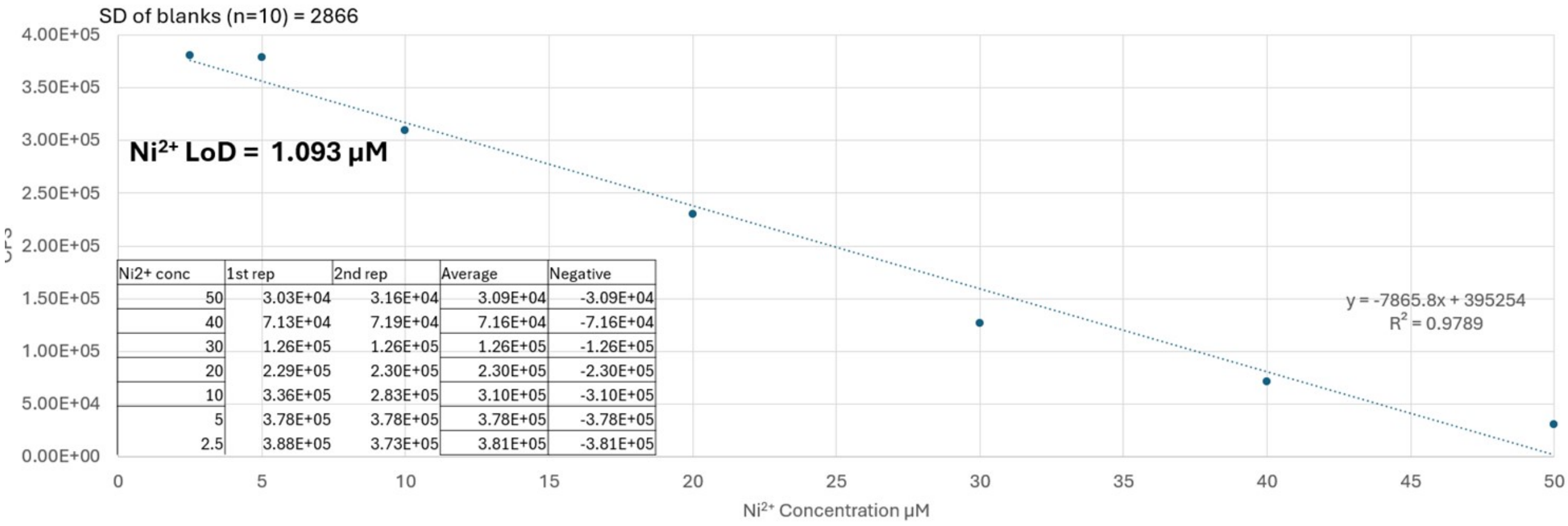

Ni2+ conc	1st rep	2nd rep	Average	Negative
50	3.03E+04	3.16E+04	3.09E+04	-3.09E+04
40	7.13E+04	7.19E+04	7.16E+04	-7.16E+04
30	1.26E+05	1.26E+05	1.26E+05	-1.26E+05
20	2.29E+05	2.30E+05	2.30E+05	-2.30E+05
10	3.36E+05	2.83E+05	3.10E+05	-3.10E+05
5	3.78E+05	3.78E+05	3.78E+05	-3.78E+05
2.5	3.88E+05	3.73E+05	3.81E+05	-3.81E+05

UV/Vis spectroscopy, 100% MeCN, C28 concentration: 50μM, Abs at 555 nm

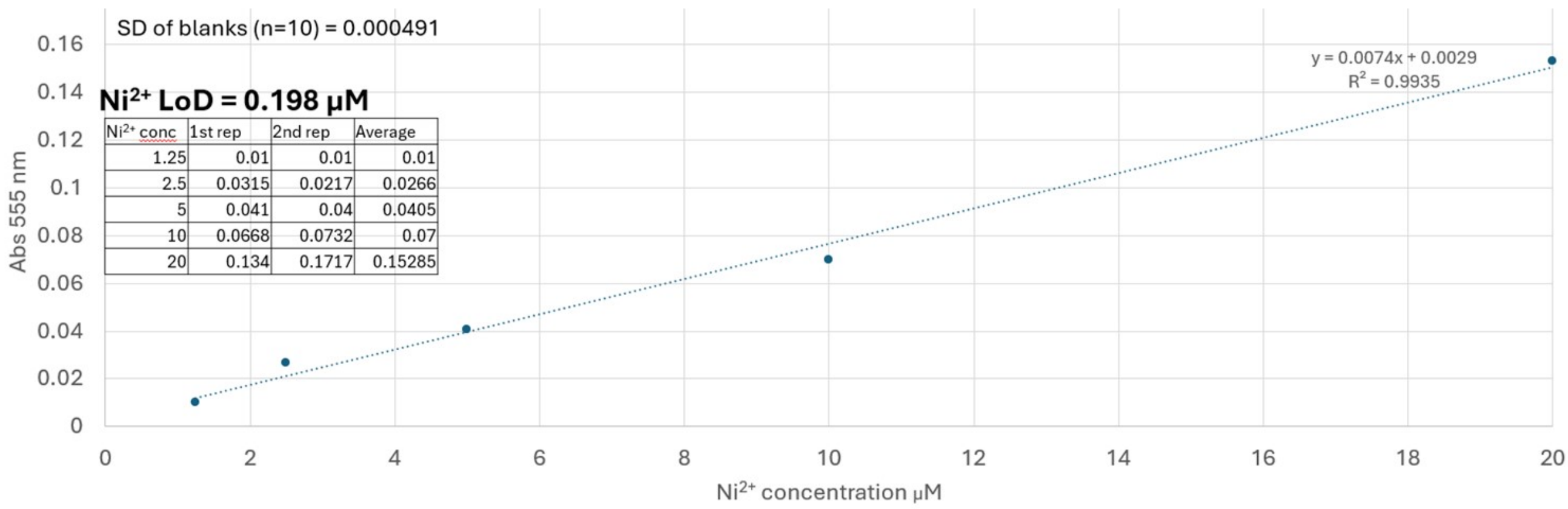

Ni^{2+} conc	1st rep	2nd rep	Average
1.25	0.01	0.01	0.01
2.5	0.0315	0.0217	0.0266
5	0.041	0.04	0.0405
10	0.0668	0.0732	0.07
20	0.134	0.1717	0.15285

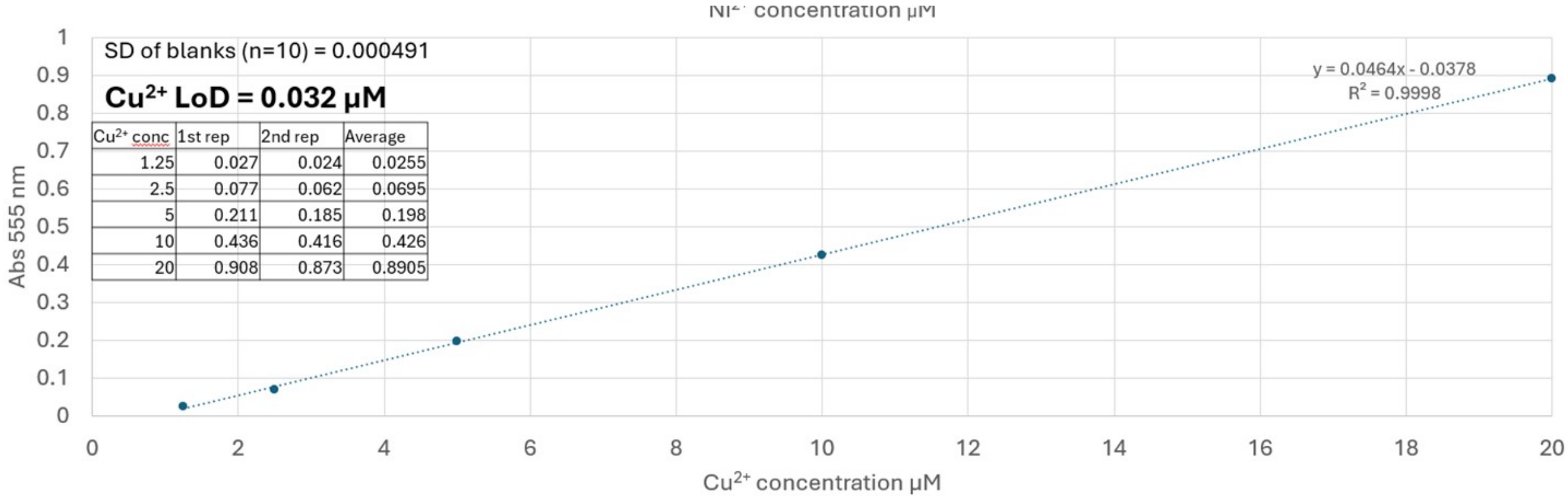

Cu^{2+} conc	1st rep	2nd rep	Average
1.25	0.027	0.024	0.0255
2.5	0.077	0.062	0.0695
5	0.211	0.185	0.198
10	0.436	0.416	0.426
20	0.908	0.873	0.8905

Job Plot (ESI S7)

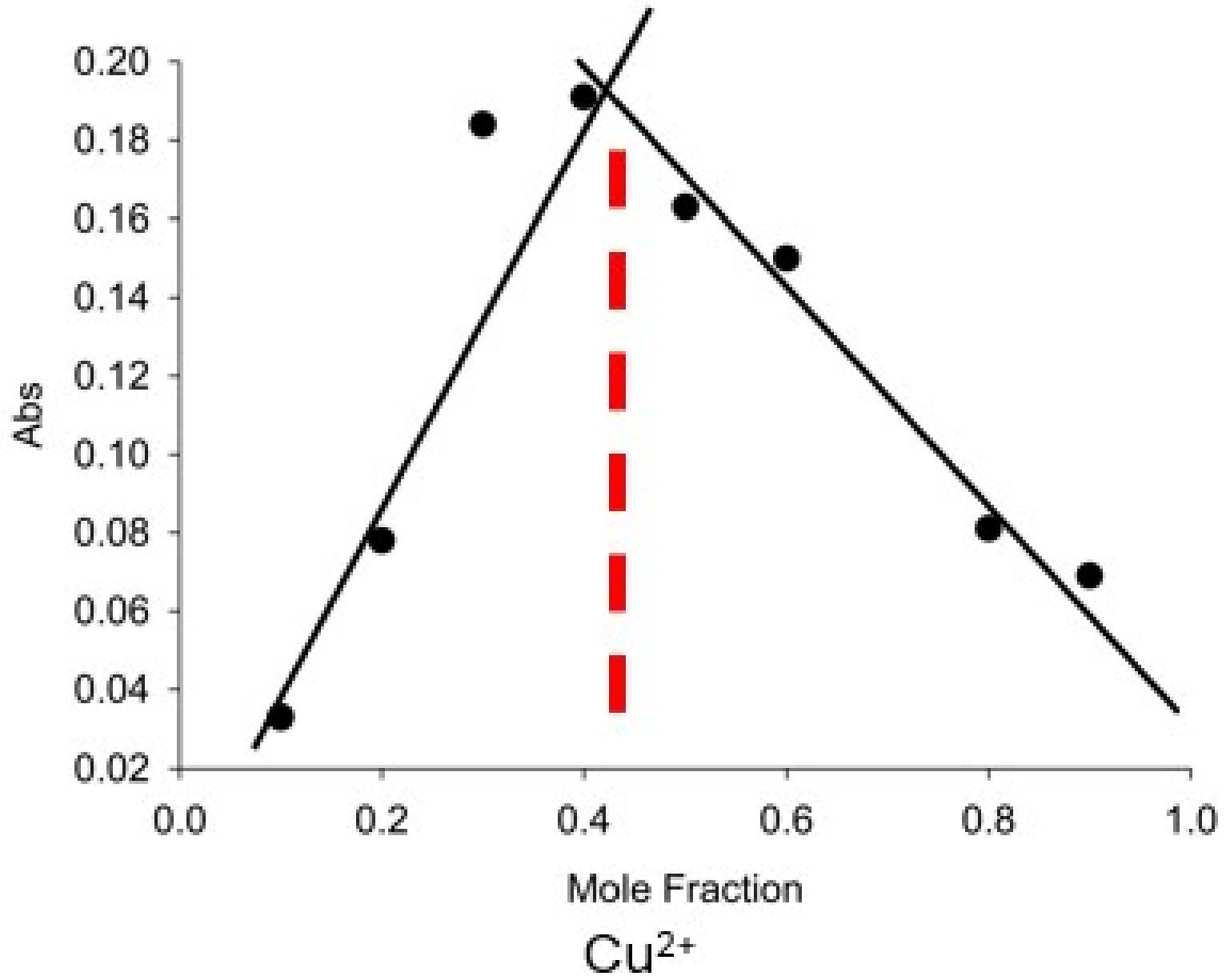

1:1 MeCN: H_2O, total concentration 50 µM

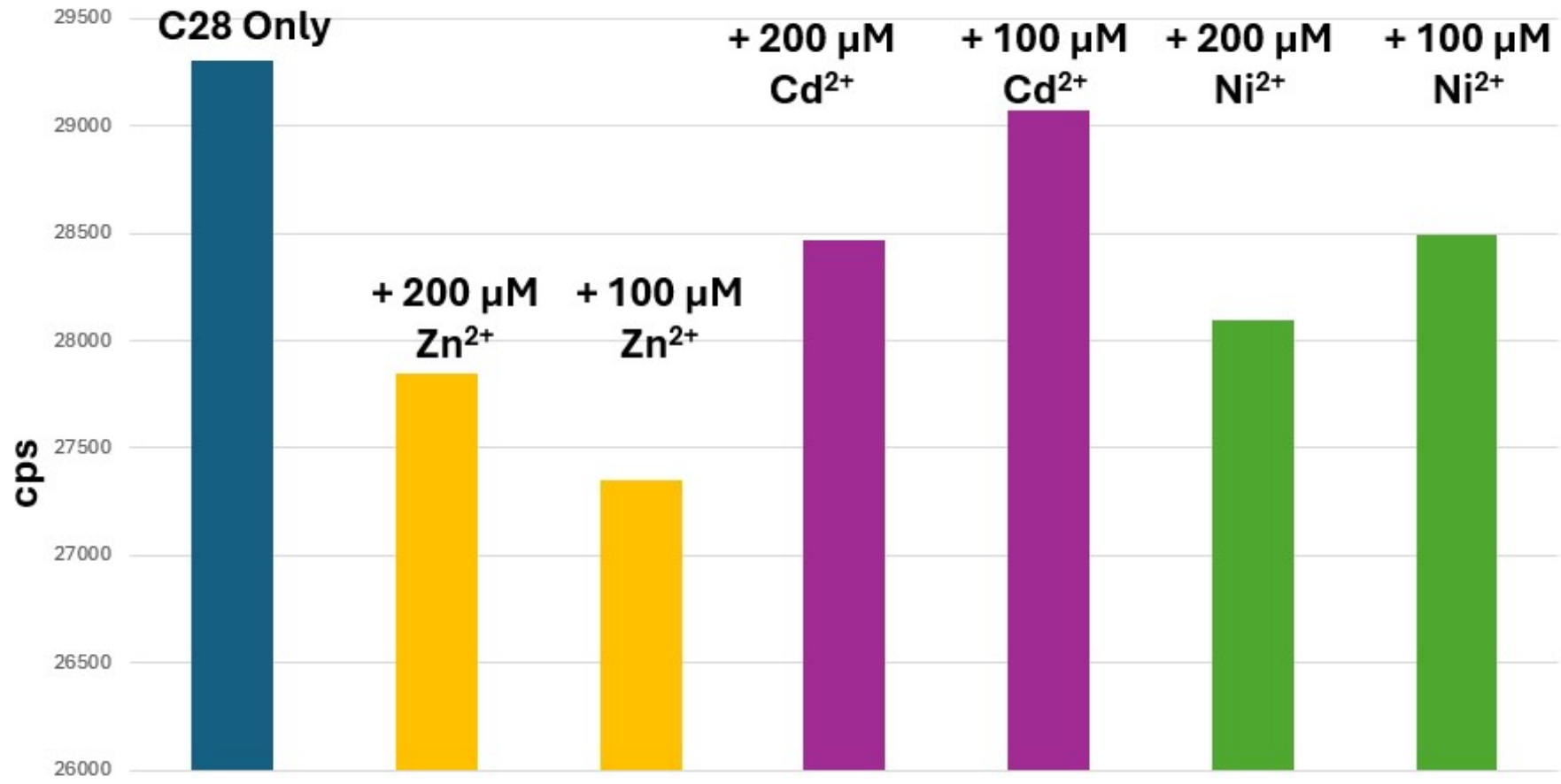

Fluorescence analysis of **C28** (50 μM) in 1:1 MeCN: H_2O with 100 μM and 200 μM Zn^{2+} indicated metal

Qualitative Limit of Detection Study for the Dipstick method

+Zn^{2+} +Cd^{2+} +Hg^{2+} +Fe^{3+} +Ru^{3+} +Pb^{2+} +Co^{2+} +Cu^{2+} +Ni^{2+}

50μM

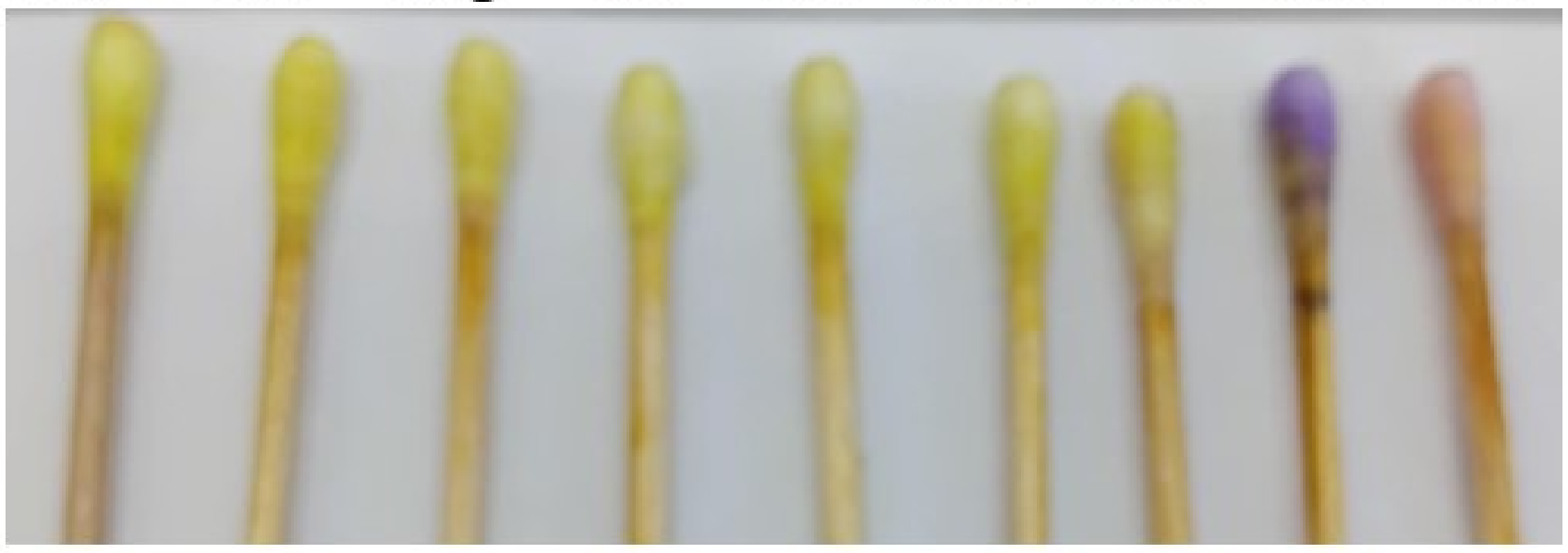

+Zn^{2+} +Cd^{2+} +Hg^{2+} +Fe^{3+} +Ru^{3+} +Pb^{2+} +Co^{2+} +Cu^{2+} +Ni^{2+}

25μM

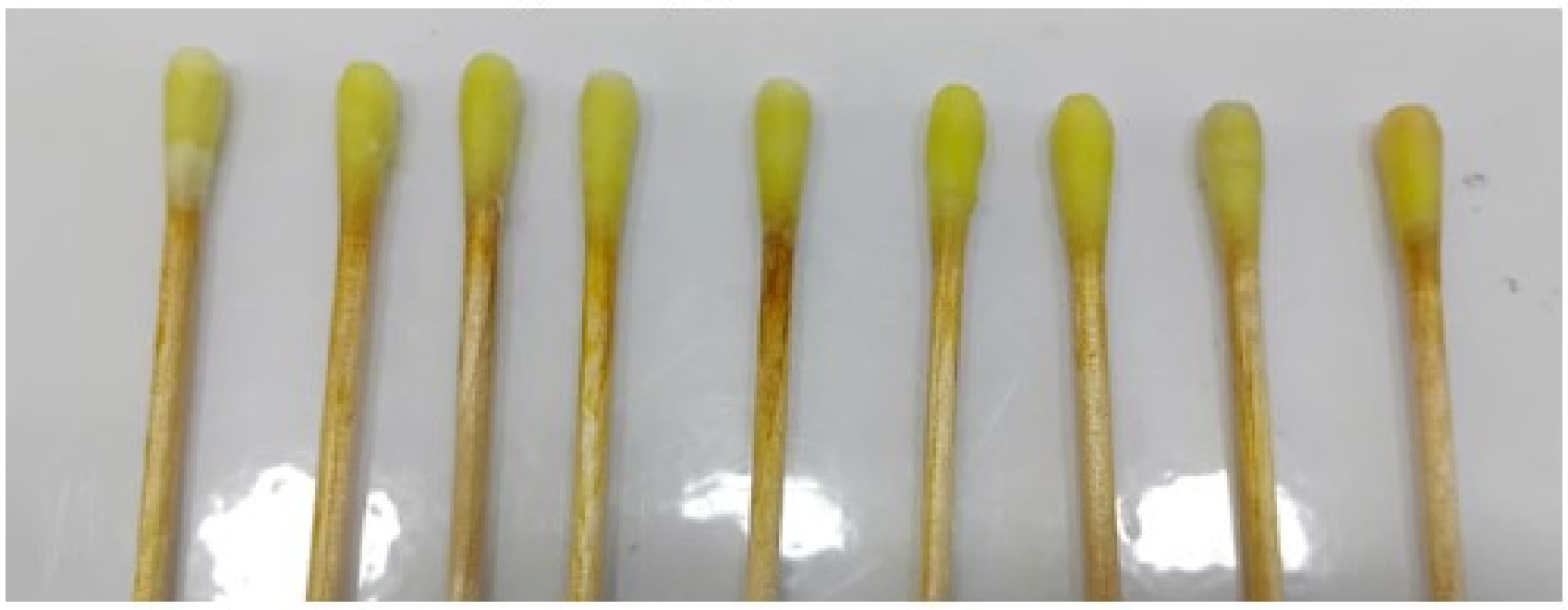

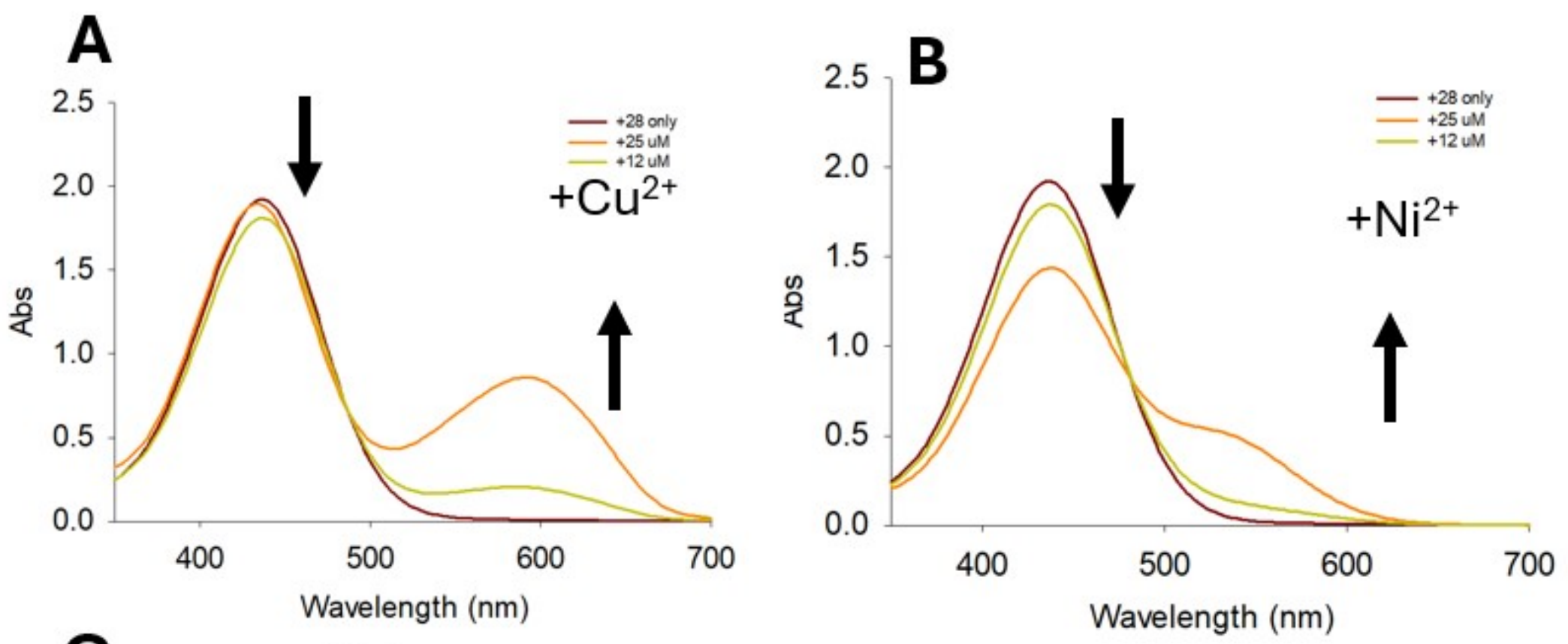

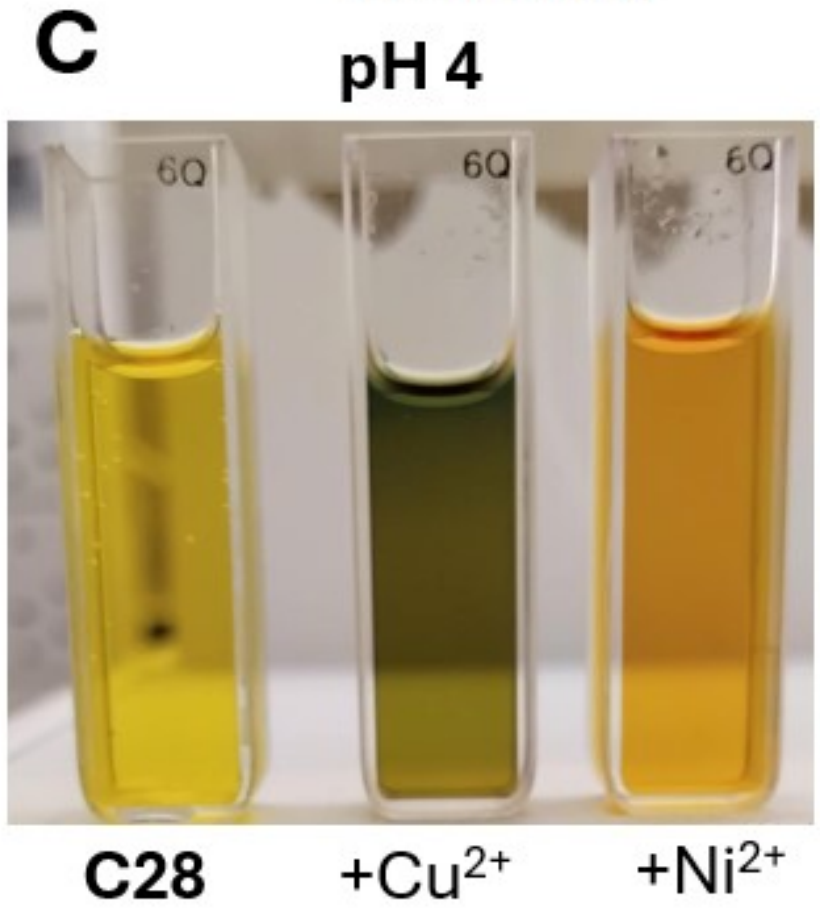

1:1 MeCN: H_2O (pH 4)
Study for C28

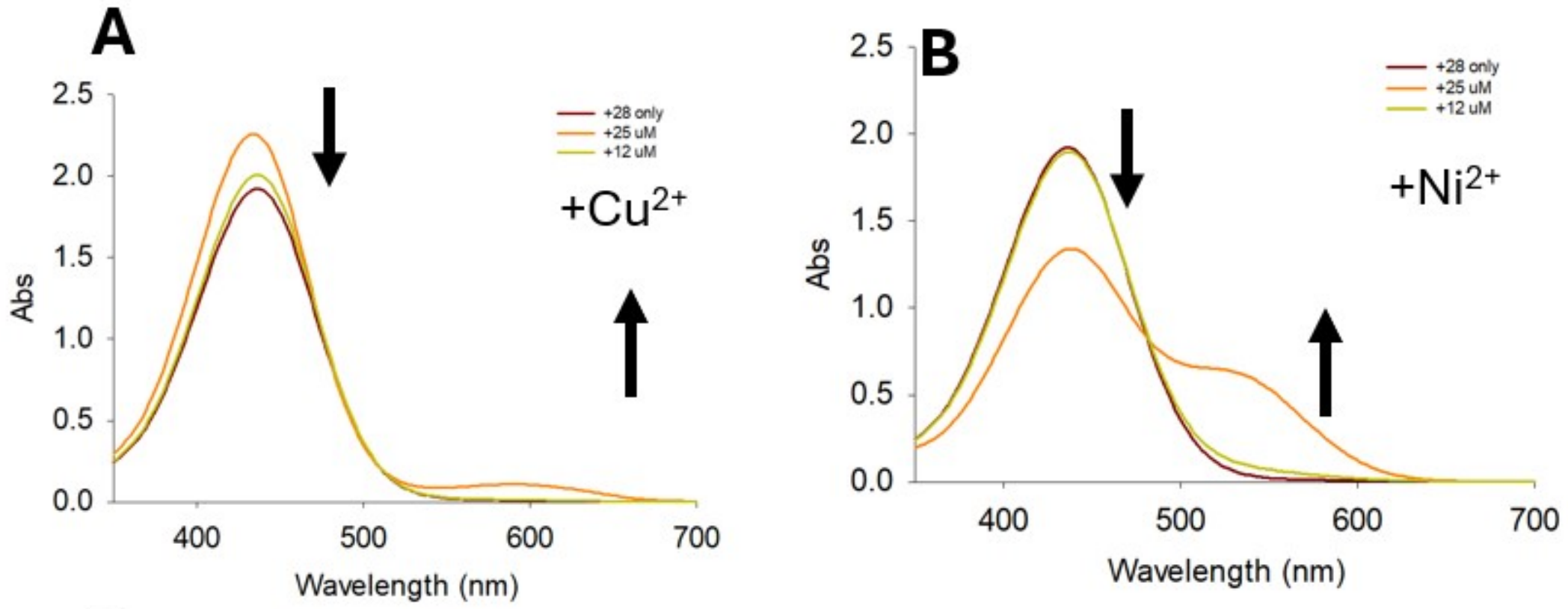

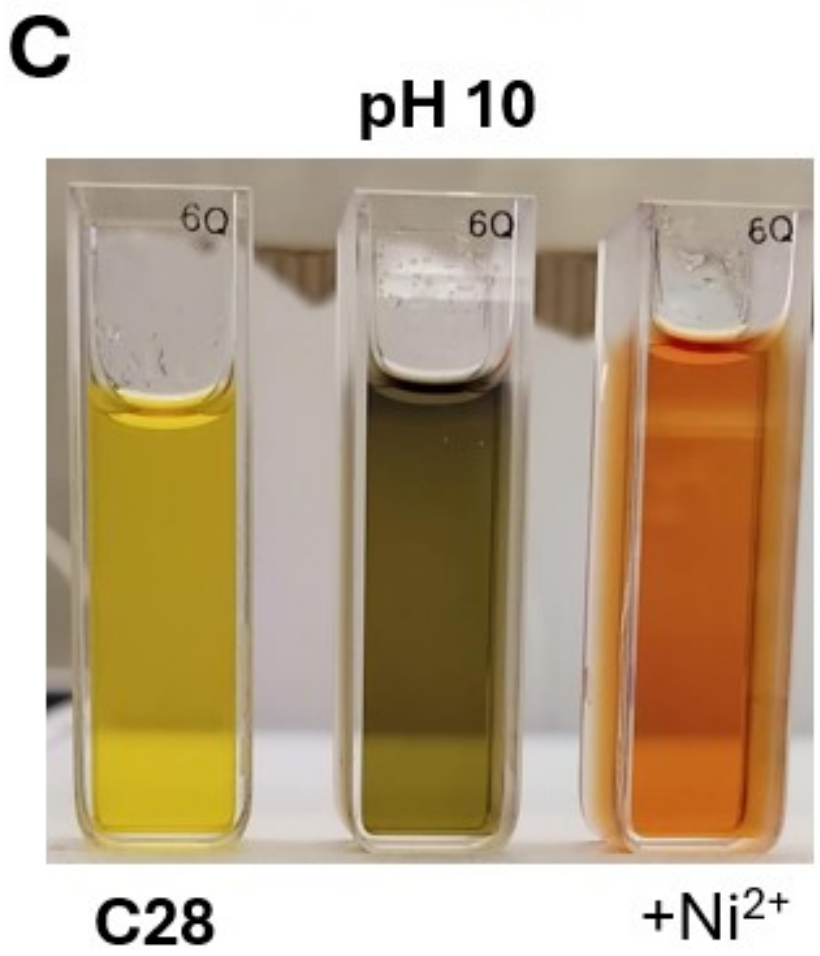

1:1 MeCN: H_2O (pH 10)
Study for C28
